# Effects of minimally invasive and traditional surgeries on the quality of life of children with congenital heart disease: a retrospective propensity score-matched study

**DOI:** 10.1186/s12887-021-02978-5

**Published:** 2021-11-24

**Authors:** Hui Tan, Erjia Huang, Xicheng Deng, Dongping Li, Shayuan Ouyang

**Affiliations:** 1 Department of Cardiovascular Surgery, The Second Xiangya Hospital, Central South University, Changsha, China; 2 Department of Cardiovascular Surgery and Clinical Nursing Teaching and Research Section, The Second Xiangya Hospital Central South University, 139 Middle Renmin Road, Changsha, 410011 China; 3 Heart Center, Hunan Children’s Hospital, Changsha, 410007 China

**Keywords:** Congenital heart disease, Minimally invasive operation, Traditional surgery, Children, Quality of life

## Abstract

**Background:**

The focus of clinical care after treating congenital heart disease (CHD) has shifted from saving patients’ lives to improving their quality of life. This study aimed to examine the influence of minimally invasive and traditional surgeries on the quality of life of children with CHD.

**Methods:**

This was a retrospective cross-sectional study. A total of 459 children aged 2–18 years with CHD treated at Second Xiangya Hospital of Central South University from July 2016 to June 2017 were enrolled, among whom 219 underwent minimally invasive surgery and 240 traditional surgery. The quality of life of children with CHD after surgery was reported by the patients’ parents. We applied propensity score matching to correct for confounding factors and conducted multiple linear regression analysis to examine the related effects of minimally invasive and traditional surgeries on the quality of life of children with CHD.

**Results:**

The scores of problems related to perceived physical appearance in children undergoing minimally invasive surgery was higher than those in those undergoing traditional surgery (*p* = 0.004). Different treatment modes were independent influencing factors for problems related to perceived physical appearance in children with CHD. There was no significant difference in average treatment effect scores of children undergoing different surgical procedures in other quality of life dimensions (problems related to cardiac symptoms and their treatment, drug treatment, anxiety regarding treatment, cognitive psychology, and communication), suggesting that different operation modes were not independent influencing factors for these related problems.

**Conclusion:**

Compared with traditional surgery, minimally invasive surgery can significantly improve the physical appearance perception scores of children with CHD after surgery. Therefore, minimally invasive surgery can improve the quality of life of children with CHD.

**Supplementary Information:**

The online version contains supplementary material available at 10.1186/s12887-021-02978-5.

## Background

The prevalence of congenital heart disease (CHD) has been increasing with approximately 1.35 million newborns suffering from CHD globally each year [1] and 150 thousand children in China. The overall prevalence rate of CHD in neonates is approximately 0.91%, the most common heart disease in this population [2]. With continuous progress in early diagnosis and surgical techniques, the life expectancy of children with CHD has shown considerable improvement [1]. Currently, 90% of patients with CHD who underwent heart surgery during childhood enter adulthood, indicating that there may be more adult than pediatric patients with CHD [3–5]. Traditional indicators for treatment outcomes, such as survival and mortality rates, reflect the treatment outcomes of children with CHD from the medical perspective. With the changes in health concepts and medical care modes, the goal of medical care is not only to maintain life and improve organ function but also to improve the quality of life (QOL), which has become another recognized evaluation index of treatment success [1, 6]. Under such circumstances, the health-related QOL of children with CHD has attracted widespread attention from scholars. Some research shows that children and adolescents with CHD have a lower QOL than do healthy individuals [7]. Research has shown that children and adolescents with CHD exhibit more behavioral and emotional problems than children from the general population, irrespective of the severity of the disease [8–11]. Landolt and Spijkerboer et al. found that children with CHD had worse motor cognition, and social and emotional functions than did healthy children [12–14]. However, the findings of Teixeira and Culbert et al. contradict those of previous studies [15, 16]. Their research revealed that the QOL of adolescents with CHD was higher than that of the general population, especially regarding environmental and social relations. The difference in these research results may be attributed to differences in methods or country of residence [17]. At present, the research on the influences of different surgical procedures on the QOL of children with CHD is still very limited. This study aimed to examine the influences of minimally invasive and traditional surgeries on the postoperative QOL of children with CHD.

## Methods

### Design

This was a cross-sectional study that examined the influences of minimally invasive and traditional surgeries on the postoperative QOL of children with CHD.

### Data and sample collection

From July 2016 to June 2017, all pediatric patients who underwent minimally invasive or traditional surgical treatment in the Department of Pediatric Cardiovascular Surgery that satisfied the inclusion criteria were invited to participate in this study. The inclusion criteria were as follows: left-to-right shunting without complications as confirmed by echocardiography and diagnosed by a doctor; patients were aged 2–18 years; patients reported no neurodevelopmental complications according to past medical history; patients received minimally invasive or traditional surgical treatment; and informed consent had been obtained from the parents. If either the child or parent had impediments in reading or understanding, or had inadequate communication skills, they were excluded. The parents of 459 children aged 2–18 years were followed up with a questionnaire (Supplement 1, 2, 3, 4, 5, 6, 7). Of them, 230 (50.1%) filled out.

questionnaires on-site at the Department of Pediatric Cardiovascular Surgery of the Second Xiangya Hospital of Central South University, and the remaining 229 questionnaires were completed through telephone follow-up (49.9%). Forty-three children were lost to follow-up because their parents could not be reached by telephone, whereas the parents of 16 children refused to complete the questionnaire survey because they were working away from home and did not know the condition of their child (*n* = 9) or because of time constraints (*n* = 7). The final analysis included a total of 400 participants, including 183 cases in the minimally invasive surgery group and 217 in the traditional surgery group. Consent to this study was obtained from the parents, and the questionnaire results were obtained from all subjects. Medical diagnoses were transcribed from inpatient medical records.

### Measurements

The parents of children with CHD completed a general information questionnaire that included sex, age, disease type, and time since the operation.

The QOL of children was investigated using the parents’ report section of the Pediatric Quality of Life Inventory Measurement Models, Cardiac Module Version 3.0 (PedsQL™ 3.0) [18–20]. The use of this scale was authorized by Mapi Research Trust. The parents’ report scale corresponded to the contents of the self-assessment questionnaire for children of corresponding age groups (2–4 years, 5–7 years, 8–12 years, and 13–18 years) and was expressed in third-person. There were 22 items in the scale that were divided into five dimensions: heart problems and symptoms, perception of physical appearance, anxiety regarding treatment, cognitive psychology, and communication problems. In addition, problems related to drug treatment were added for children who were taking medication.

The development of the PedsQL was initiated by Professor James W. Varni of the United States in 1987 and includes a universal module and disease-specific module with a mature scale system, and PedsQL has been applied in many countries and regions [21]. The disease-specific module, PedsQL 3.0, is a commonly used scale for measuring the QOL of children with heart disease in various countries [22–25]. It has been repeatedly evaluated and applied, and has been proven to have good reliability and validity. Every item in the PedsQL 3.0 scale questions the frequency of events in the previous month. The PedsQL 3.0 is scored on a 5-point scale (0–4). The total score and scores in all aspects are between 0 and 100, and higher scores indicate a higher QOL (Supplement 8).

### Data analysis

**Fig. 1:**
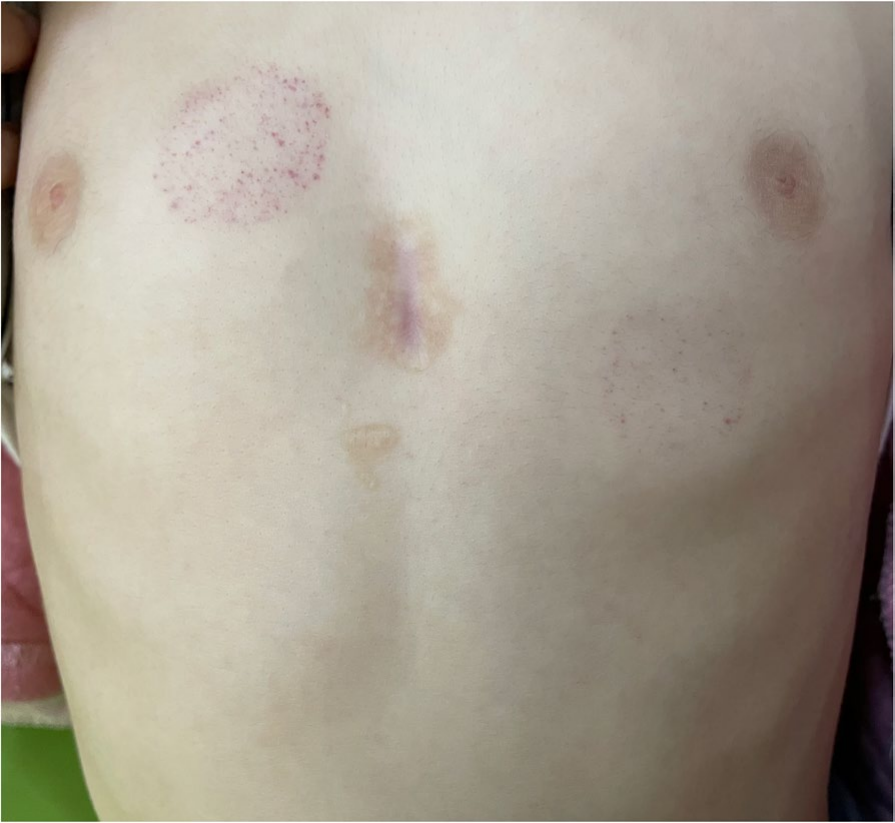
Postoperative scar of a patient who received minimally invasive surgery

**Fig. 2:**
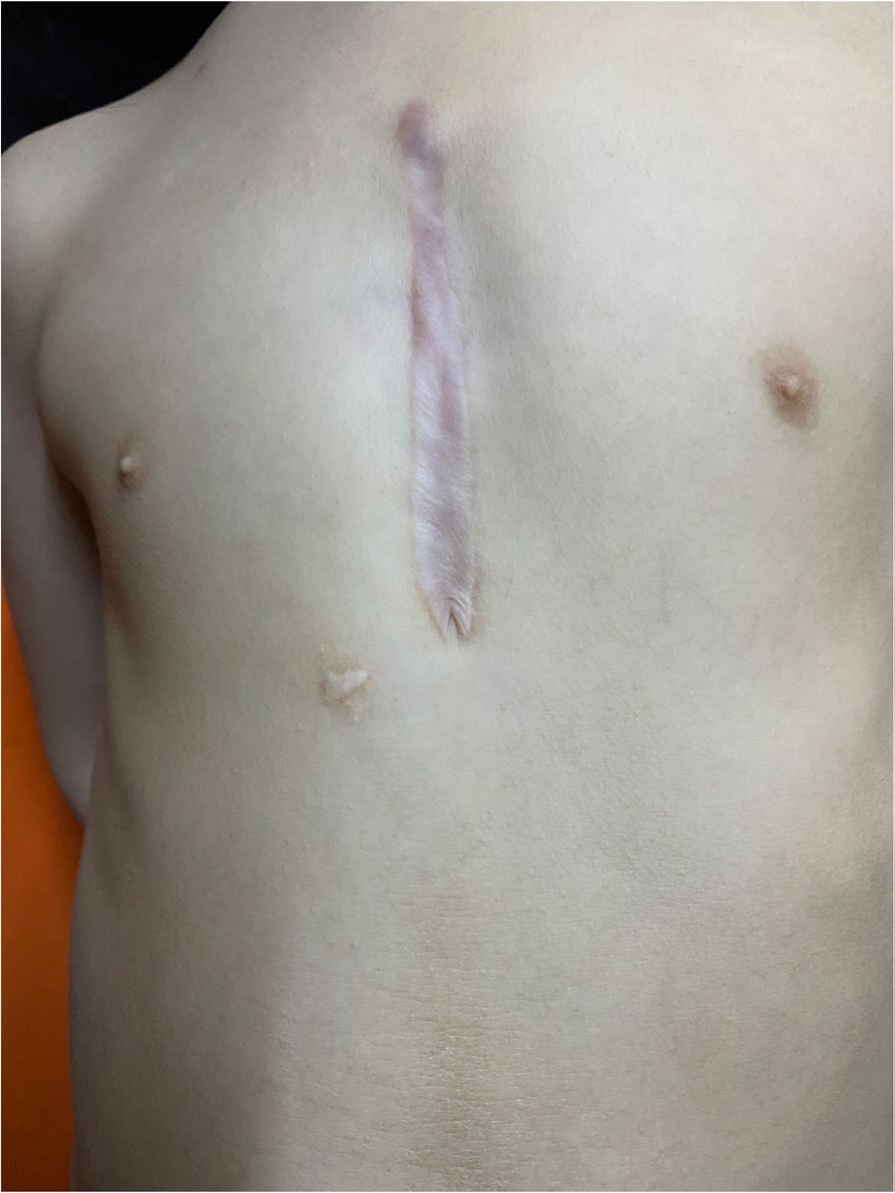
Postoperative scar of a patient who received traditional surgery

General information on the children, such as demographics, were presented as frequencies, percentages, means, and standard deviation. The χ^2^ or two-sample independent *t-*tests were used to compare the differences in baseline characteristics between the two treatment groups. The children’s QOL scores did not conform to a normal distribution. Median and polar distance were used for descriptive analysis, and the Wilcoxon rank sum test was used to compare the differences in QOL score between the traditional and minimally invasive surgery groups. Traditional surgery was performed through a full median sternotomy and under cardiopulmonary bypass. Minimally invasive surgery was performed through a mini-sternotomy and the defect was occluded with a metal occluder delivered through the surface of the heart. Figures 1 and 2 show the scars after the two different surgeries. The difference in QOL score between children undergoing the two treatment modes were analyzed and compared according to age (2–4 years, 5–7 years, 8–12 years, and 13–18 years) and postoperative time (≤1 month, 1–6 months, 6–12 months, 12–36 months, and > 36 months). Considering the unbalanced baseline conditions of children in different treatment groups, propensity score matching (PSM) was used to control for confounding factors. After matching, the standardized mean difference (SMD) of general information was controlled within 10%, and the covariates were age, sex, body mass index (BMI), CHD type, and postoperative time. The average treatment effect (ATE) of matched samples was analyzed and tested. Multiple linear regression was used to analyze the independent factors influencing the children’s QOL for the matched samples, and the dependent variables were the logarithmic values of QOL scores for these dimensions. SAS 9.4 and R 3.5.3 software were used for statistical analysis.

## Results

The minimally invasive group comprised 72 males and 111 females. The average age of patients in the minimally invasive group was 6.5 ± 3.5 years, and there were 20 patients with ventricular septal defects (VSDs), 144 with atrial septal defects (ASDs), and 19 with other conditions. The traditional surgery group comprised 112 males and 105 females. The average age of patients in the traditional surgery group was 5.4 ± 3.1 years, and there were 42 patients with VSDs, 173 with ASDs, and 2 with other conditions (Table 1).

**Table 1:** General demographic and clinical characteristics of children with congenital heart disease who received traditional surgery or minimally invasive surgery whose parents have completed questionnaires (*n* = 400)

General information indicators	Treatment groups	
	TS (*n* = 217)	MIS (*n* = 183)
Age (years, x ®±s)	5.4 ± 3.1	6.5 ± 3.5
Age group (n, %)		
2–4 years	137 (63.1)	72 (39.3)
5–7 years	52 (24.0)	66 (36.1)
8–12 years	15 (6.9)	36 (19.7)
13–18 years	13 (6.0)	9 (4.9)
Sex (n, %)		
Male	112 (51.6)	72 (39.3)
Female	105 (48.4)	111 (60.7)
BMI (x ± s)	16.1 ± 3.4	15.8 ± 3.3
Type of congenital heart disease (n, %)		
ASD	42 (19.4)	20 (10.9)
VSD	173 (79.7)	144 (78.7)
Other	2 (0.9)	19 (10.4)
Time after operation (months, x ± s)	19.1 ± 20.1	22.1 ± 20.8

*TS* Traditional surgery, *MIS* Minimally invasive surgery, *BMI* body mass index, *ASD* atrial septal defect, *VSD* ventricular septal defect

There were significant differences in the scores of children’s problems related to cardiac symptoms and their treatment (*p* = 0.0069), drug treatment (*p* = 0.0395), and perception of physical appearance (*p* = 0.0150) between the traditional and minimally invasive surgery groups. Subgroup analysis by age showed significant differences in scores of problems related to cardiac symptoms and their treatment (*p* = 0.0366), and perception of physical appearance (*p* = 0.0089) among children aged 5–7 years, and cardiac symptoms and their treatment (*p* = 0.0396), drug treatment (*p* = 0.0095), perception of physical appearance (*p* = 0.0228), anxiety regarding treatment (*p* = 0.0072), and communication (*p* = 0.0017) among children aged 8–12 years (*p* < 0.05) (Table 2).

**Table 2:** Quality of life scores of children with congenital heart disease who received traditional surgery and minimally invasive surgery reported by parents by different age groups [Median (range)]

Item		Problems related to cardiac symptoms and their treatment	Drug treatment problems	Problems related to perceived physical appearance	Problems related to anxiety about treatment	Problems related to cognitive psychology	Problems related to communication
All ages	TS (*n* = 217)	93(50–100)	100(0–100)	100(0–100)	100(13–100)	90 (15–100)	100 (0–100)
	MIS (*n* = 183)	93 (36–100)	100 (8–100)	100 (25–100)	100 (25–100)	90 (10–100)	100 (0–100)
	*P*	**0.0069**	**0.0395**	**0.015**	0.1413	0.7419	0.108
2–4 years	TS (*n* = 137)	93 (57–100)	100 (0–100)	100 (25–100)	100 (13–100)	100 (17–100)	100 (0–100)
	MIS (*n* = 72)	91 (50–100)	100 (8–100)	100 (50–100)	100 (25–100)	91 (33–100)	100 (0–100)
	*P*	0.8501	0.493	0.2803	0.487	0.3804	0.2275
5–7 years	TS (*n* = 52)	93 (57–100)	100 (58–100)	100 (33–100)	100 (56–100)	90 (15–100)	100 (0–100)
	MIS (*n* = 66)	96 (71–100)	100 (92–100)	100 (42–100)	100 (31–100)	95 (35–100)	100 (33–100)
	*P*	**0.0366**	0.123	**0.0089**	0.3442	0.1315	**0.0986**
8–12 years	TS (*n* = 15)	86 (50–100)	100 (75–100)	83 (0–100)	81 (31–100)	70 (20–100)	75 (0–100)
	MIS (*n* = 36)	93 (35–100)	100 (80–100)	100 (25–100)	100 (44–100)	85 (10–100)	100 (17–100)
	*P*	**0.0396**	**0.0095**	**0.0228**	**0.0072**	0.1288	**0.0017**
13–18 years	TS (*n* = 13)	79 (50–100)	95 (70–100)	92 (33–100)	81 (31–100)	75 (40–100)	100 (42–100)
	MIS (*n* = 9)	93 (57–100)	100 (80–100)	100 (75–100)	94 (56–100)	80 (30–100)	100 (83–100)
	*P*	0.1307	0.0737	0.28	0.3581	0.6868	0.5226

*TS* Traditional surgery, *MIS* Minimally invasive surgery, #: *P* value was calculated using Wilcoxon rank sum test

There was no difference in the scores for various dimensions between the minimally invasive and traditional surgery groups at 1 month (*p* = 0.5946), 1–6 months, and 6–12 months after surgery (*p* > 0.05). At 12–36 months after the operation, the scores of problems related to perceived physical appearance in the minimally invasive surgery group were higher than those in the traditional surgery group (*p* = 0.0399). After > 36 months, the scores of problems related to cardiac symptoms and their treatment (*p* = 0.0260), drug treatment (*p* = 0.0216), anxiety regarding treatment (*p* = 0.0489), cognitive psychology (*p* = 0.0120), and communication (*p* = 0.0009) in the minimally invasive surgery group were higher than those in the traditional surgery group (*p* < 0.05) (Table 3).

**Table 3:** Quality of life scores of children who received traditional surgery and minimally invasive surgery reported by parents by different postoperative period [Median (range)]

Item		Problems related to cardiac symptoms and their treatment	Drug treatment problems	Problems related to perceived physical appearance	Problems related to anxiety about treatment	Problems related to cognitive psychology	Problems related to communication
≤1 month postoperatively	TS(*n* = 62)	88 (50–100)	100 (0–100)	100 (0–100)	94 (25–100)	85 (17–100)	83 (0–100)
	MIS(*n* = 49)	89 (50–100)	100 (8–100)	100 (25–100)	88 (25–100)	83 (30–100)	83 (0–100)
	*P*^*#*^	0.5946	0.2369	0.4965	0.6774	0.9568	0.8797
1–6 months postoperatively	TS(*n* = 7)	93 (57–100)	100 (90–100)	92 (75–100)	94 (69–100)	75 (40–100)	100 (42–100)
	MIS(*n* = 15)	86 (61–100)	100 (75–100)	100 (75–100)	100 (69–100)	95 (50–100)	100 (0–100)
	*P*^*#*^	0.8029	0.1635	0.1348	0.4712	0.2091	0.6634
6–12 months postoperatively	TS(n22=)	89 (57–100)	100 (50–100)	96 (25–100)	78 (38–100)	75 (15–100)	75 (0–100)
	MIS(*n* = 8)	91 (71–100)	100 (92–100)	100 (33–100)	81 (44–100)	87 (45–100)	79 (33–100)
	*P*^*#*^	0.5068	0.2398	0.2641	0.9621	0.3948	0.5118
12–36 months postoperatively	TS(*n* = 112)	93 (57–100)	100 (42–100)	100 (33–100)	100 (13–100)	100 (30–100)	100 (0–100)
	MIS(*n* = 87)	96 (68–100)	100 (75–100)	100 (42–100)	100 (31–100)	92 (35–100)	100 (42–100)
	*P*^*#*^	**0.0096**	0.4546	**0.0339**	0.3231	0.2009	0.9242
> 36 months postoperatively	TS(*n* = 14)	93 (64–96)	100 (83–100)	88 (33–100)	100 (44–100)	71 (20–100)	88 (0–100)
	MIS(*n* = 24)	96 (79–100)	100 (100–100)	96 (50–100)	100 (50–100)	90 (10–100)	100 (50–100)
	*P*^*#*^	**0.026**	**0.0216**	0.7347	**0.0489**	**0.012**	**0.0009**

*TS* Traditional surgery, *MIS* Minimally invasive surgery,#: *P* value was calculated using Wilcoxon rank sum test

After PSM of the children’s QOL scores, 136 children from each group were successfully matched. After matching, the SMD values of the two groups were below 10% except for the postoperative time, and the matching status was acceptable. The demographic clinical characteristics are shown in Table 4.

**Table 4:** General demographic and clinical characteristics of children with congenital heart disease who received traditional surgery and minimally invasive surgery reported by parents on their behalf after propensity score matching

General information indicators	Treatment mode		SMD (%)
	TS (*n* = 136)	MIS (*n* = 136)	
Age (years, x ®±s)	5.80 ± 3.38	5.89 ± 2.64	2.3
Sex (n, %)			7.4
Male	79 (58.1)	74 (54.4)	
Female	57 (41.9)	62 (45.6)	
BMI (x ± s)	15.7 ± 2.54	15.6 ± 3.13	1.3
Type of congenital heart disease (n,%)			2.1
ASD	19 (14.0)	18 (13.2)	
VSD	115 (84.6)	116 (85.3)	
Other	2 (1.5)	2 (1.5)	
Time after operation (months, x ± s)	20.0 ± 20.7	22.1 ± 20.9	10.0

*TS* Traditional surgery, *MIS* Minimally invasive surgery, *BMI* body mass index, *ASD* atrial septal defect, *VSD* ventricular septal defect, *SMD* standardized mean difference

Comparing the QOL average treatment effect on the treated (ATT) scores of children with CHD between the minimally invasive and traditional surgery groups after matching, the ATT scores of children who underwent minimally invasive surgery was higher than that of those who underwent traditional surgery (*p* = 0.0043). There were no significant differences in ATT scores of children with different surgical procedures in other QOL dimensions including problems related to cardiac symptoms and their treatment (*p* = 0.1009), drug treatment (*p* = 0.2334), anxiety regarding treatment (*p* = 0.1243), cognitive psychology (*p* = 0.7924), and communication (*p* = 0.5939) (Table 5).

**Table 5:** Quality of life ATT scores of children with congenital heart disease who received traditional surgery and minimally invasive surgery reported by parents on their behalf after propensity score matching

Item	ATT		*P*
	Log (mean)	95% CI	
Problems related to cardiac symptoms and their treatment	0.0298	−0.0058-0.0655	0.1009
Drug treatment problems	0.0489	−0.0318-0.1298	0.2334
Problems related to perceived physical appearance	0.1305	0.0412–0.2198	**0.0043**
Problems related to anxiety about treatment	0.0478	−0.0132-0.1089	0.1243
Problems related to cognitive psychology	0.0095	−0.0618-0.0810	0.7924
Problems related to communication	0.0562	−0.1513-0.2639	0.5939

*ATT* average treatment effect, *P* < 0.05 suggesting statistical significance

Multiple linear regression analysis found that different treatment modes (*p* = 0.003) and ages (*p* < 0.001) were independent influencing factors of problems related to children’s perceived physical appearance reported by their parents. Different treatment modes were not independent influencing factors of scores of other QOL dimensions including male sex (reference = female) (*p* = 0.229), BMI (*p* = 0.099), time since the operation (*p* = 0.730), problems related to communication (*p* = 0.701), VSDs (reference = ASDs) (*p* = 0.072), or other types of diseases (reference = ASDs) (*p* = 0.591) (Table 6).

**Table 6:** Results of multiple linear regression analysis on factors influencing children’s quality of life reported by parents on their behalf

	Problems related to cardiac symptoms and their treatment		Drug treatment problems		Problems related to perceived physical appearance		Problems related to anxiety about treatment		Problems related to cognitive psychology		Problems related to communication	
	b	*P*	b	*P*	b	*P*	b	*P*	b	*P*	b	*P*
MIS (reference = TS)	0.0257	0.135	0.0414	0.309	0.1296	**0.003**	0.0463	0.135	0.0127	0.716	0.0390	0.701
Age	−0.0137	**< 0.001**	0.0088	0.226	−0.0287	**< 0.001**	−0.0060	0.277	−0.0102	0.103	0.0187	0.307
Male (reference = female)	0.0511	**0.004**	0.0516	0.216	0.0541	0.229	0.0296	0.349	0.0198	0.578	0.1479	0.156
BMI	−0.0004	0.883	−0.0041	0.577	0.0130	0.099	0.0083	0.132	0.0169	**0.007**	0.0383	**0.036**
Time after operation	0.0016	**< 0.001**	0.0024	**0.028**	0.0004	0.730	0.0002	0.728	−0.0016	0.083	0.0051	0.063
VSD (reference = ASD)	−0.0039	0.882	−0.0395	0.526	0.1216	0.072	0.0786	0.098	0.1372	**0.011**	0.1175	0.452
Other types of diseases (reference = ASD)	−0.0222	0.783	−0.1538	0.423	0.1114	0.591	0.0183	0.900	−0.2179	0.187	−1.7654	**< 0.001**

*TS* Traditional surgery, *MIS* Minimally invasive surgery, *BMI* body mass index, *VSD* ventricular septal defect, *ASD* atrial septal defect, *P* < 0.05 suggesting statistical significance

## Discussion

Multiple studies have been conducted on postoperative QOL of patients with CHD, but results are inconsistent. Patients with CHD score similar to or better than their healthy peers in QOL questionnaires [26–28], but some studies argue that QOL might be compromised [15, 29] The disagreement may have arisen from heterogeneity in subject demographics, the questionnaire used, specific CHD, or country of origin. Our study is the first to focus on the impact of different surgical methods on CHD repair. Our results demonstrated that the QOL of children in the minimally invasive surgery group was higher than that of those in the traditional surgery group with respect to the scores of problems related to cardiac symptoms and their treatment, drug treatment, and perception of physical appearance (< 0.05). This may be related to traditional surgical procedures that use median sternotomy and cardiopulmonary bypass, which can cause more complications and leave a long scar after the operation, whereas minimally invasive surgery is characterized by less trauma, quick recovery time, and no large incision of a median sternotomy, cardiopulmonary bypass, and vascular-related injuries [30–32].

There were no significant differences in the scores of problems related to anxiety regarding treatment, cognitive psychology, or communication between the two groups. Some results are consistent with those from the study by Sarrechia et al. [33]. Although the cardiovascular malformation of children with CHD had been corrected by surgery, their psychological and social problems had not disappeared following the correction of malformation, and these problems continued to affect children with CHD. This result is similar to that obtained by Wang and Li et al. [34, 35]. This emphasizes the need for psychological assessment of children with CHD by clinical medical staff to identify psychological and social problems as soon as possible and provide targeted intervention measures. In addition, it is necessary to provide health education to parents on the psychological and social aspects of CHD.

Our study showed no differences in QOL scores between children with CHD treated with minimally invasive or traditional surgery at 1 month, 1–6 months, and 6–12 months after surgery. Children with CHD and their parents may have mainly considered whether the cardiac malformation was corrected and whether the symptoms of CHD were improved at the early stages after the operation. Both minimally invasive and traditional surgical treatments can achieve ideal treatment outcomes for improving children’s hemodynamics [32, 33]. At 12–36 months after the operation, children in the minimally invasive surgery group scored higher than those in the traditional surgery group (*p* < 0.05), indicating that with the correction of cardiac malformation and recovery of cardiac function, the focus of children with CHD and their parents’ perception of their QOL began to shift toward their physical appearance, and permanent scarring was an important factor affecting the QOL at this stage. Children in the minimally invasive surgery group scored higher than those in the traditional surgery group, reflecting the advantage of minimally invasive surgery. However, after 36 months the scores of problems related to cardiac symptoms and their treatment, drug treatment, anxiety regarding treatment, cognitive psychology, and communication in the minimally invasive surgery group were higher than those in the traditional surgery group (*p* < 0.05). This suggests that the QOL of children with CHD in different periods was dynamic. After 36 months, the QOL of children with CHD treated with minimally invasive surgery was higher in the physiological and psychosocial aspects than that of those treated with traditional surgery, and this difference showed a continuous increasing trend with time, indicating a clear treatment effect of minimally invasive treatment in the medium- and long-term [12, 13, 31].

The multiple linear regression analysis results in this study revealed that different treatment modes were independent influencing factors for problems related to the perception of physical appearance in children with CHD but were not independent influencing factors for other dimensions of their QOL. This result suggests that the advantage of less trauma in the minimally invasive surgery group was related to the increased demands of children with CHD and their parents regarding physical appearance-related problems after the correction of cardiac malformation. This emphasizes the need for attention to wound management for children with CHD who have undergone traditional surgery. To ensure surgical safety, the length of the incision should be minimized as much as possible; wound management should be strengthened after surgery to include extensive health education on wound care at home and encouraging children with CHD to visit physicians at the dermatology and cosmetology department. Attention should be paid to the aesthetics of wounds in children with CHD to reduce the negative impact of permanent scars on the QOL of children with CHD.

## Limitations

Our study has several limitations. Firstly, as this study is a cross-sectional study, we cannot assess any fluctuations of QOL over time, thus future studies including long-term follow-up is required. Secondly, the dropout rate was as high as 14.8%, thus potential bias could not be ignored. Thirdly, our data were completely obtained from parental reports, which need to be further studied in combination with self-reported data from children. Lastly, because this study is not a randomized controlled study, although PSM was adopted, the influence of confounding factors could not be completely ruled out.

## Conclusion

The results of our study show that problems related to the perception of physical appearance were an important factor influencing the postoperative QOL of children with CHD. Compared with traditional surgery, minimally invasive surgery can significantly improve the score of perceived physical appearance and improve the postoperative QOL of children with CHD. For children with CHD who are not eligible for minimally invasive surgery, medical staff should strengthen wound management to reduce the negative impact of permanent scars on the QOL. Furthermore, these findings highlight the need for future studies with larger sample sizes and long-term continuous follow-up to help formulate more targeted nursing interventions to improve the QOL of children with CHD.

## Supplementary Information

**Additional file 1.**

**Additional file 2.**

**Additional file 3.**

**Additional file 4.**

**Additional file 5.**

**Additional file 6.**

**Additional file 7.**

**Additional file 8.**

**Additional file 9.**

**Additional file 10.**

**Additional file 11.**

**Additional file 12.**

**Additional file 13.**

## Data Availability

The dataset necessary to interpret, replicate and build upon the findings reported in the article will be made available on reasonable request and can be obtained by contacting the corresponding author.

## Abbreviations

BMI: Body mass index
ASD: Atrial septal defect
VSD: Ventricular septal defect
CHD: Congenital heart disease
QOL: Quality of Life
ATT: Average treatment effect on the treated

## References

[1] Uzark K (2016). Challenges of assessing quality of life in congenital heart disease globally. J Am Coll Cardiol.

[2] Marelli AJ, Ionescu-Ittu R, Mackie AS, Guo L, Dendukuri N, Kaouache M (2014). Lifetime prevalence of congenital heart disease in the general population from 2000 to 2010. Circulation..

[3] van der Linde D, Konings EE, Slager MA, Witsenburg M, Helbing WA, Takkenberg JJ (2011). Birth prevalence of congenital heart disease worldwide: a systematic review and meta-analysis. J Am Coll Cardiol.

[4] Dolk H, Loane M, Garne E (2011). European surveillance of congenital anomalies working G. congenital heart defects in Europe: prevalence and perinatal mortality, 2000 to 2005. Circulation..

[5] Gaskin K, Kennedy F (2019). Care of infants, children and adults with congenital heart disease. Nurs Stand.

[6] Im YM, Yun TJ, Lee S (2018). Health condition and familial factors associated with health-related quality of life in adolescents with congenital heart disease: a cross sectional study. Health Qual Life Outcomes.

[7] Mellion K, Uzark K, Cassedy A, Drotar D, Wernovsky G, Newburger JW (2014). Health-related quality of life outcomes in children and adolescents with congenital heart disease. J Pediatr.

[8] Fredriksen PM, Mengshoel AM, Frydenlund A, Sorbye O, Thaulow E (2004). Follow-up in patients with congenital cardiac disease more complex than haemodynamic assessment. Cardiol Young.

[9] Hovels-Gurich HH, Konrad K, Wiesner M, Minkenberg R, Herpertz-Dahlmann B, Messmer BJ (2002). Long term behavioural outcome after neonatal arterial switch operation for transposition of the great arteries. Arch Dis Child.

[10] Janus M, Goldberg S (1995). Sibling empathy and behavioural adjustment of children with chronic illness. Child Care Health Dev.

[11] Utens EM, Verhulst FC, Meijboom FJ, Duivenvoorden HJ, Erdman RA, Bos E (1993). Behavioural and emotional problems in children and adolescents with congenital heart disease. Psychol Med.

[12] Landolt MA, Valsangiacomo Buechel ER, Latal B (2008). Health-related quality of life in children and adolescents after open-heart surgery. J Pediatr.

[13] Spijkerboer AW, Utens EM, De Koning WB, Bogers AJ, Helbing WA, Verhulst FC (2006). Health-related quality of life in children and adolescents after invasive treatment for congenital heart disease. Qual Life Res.

[14] Walker RE, Gauvreau K, Jenkins KJ (2004). Health-related quality of life in children attending a cardiology clinic. Pediatr Cardiol.

[15] Teixeira FM, Coelho RM, Proenca C, Silva AM, Vieira D, Vaz C (2011). Quality of life experienced by adolescents and young adults with congenital heart disease. Pediatr Cardiol.

[16] Culbert EL, Ashburn DA, Cullen-Dean G, Joseph JA, Williams WG, Blackstone EH (2003). Quality of life of children after repair of transposition of the great arteries. Circulation..

[17] Apers S, Kovacs AH, Luyckx K, Alday L, Berghammer M, Budts W (2015). Assessment of patterns of patient-reported outcomes in adults with congenital heart disease - international study (APPROACH-IS): rationale, design, and methods. Int J Cardiol.

[18] Varni JW, Burwinkle TM, Seid M, Skarr D (2003). The PedsQL 4.0 as a pediatric population health measure: feasibility, reliability, and validity. Ambul Pediatr.

[19] Ladak LA, Hasan BS, Gullick J, Awais K, Abdullah A, Gallagher R (2019). Health-related quality of life in surgical children and adolescents with congenital heart disease compared with their age-matched healthy sibling: a cross-sectional study from a lower middle-income country, Pakistan. Arch Dis Child.

[20] Tahirovic E, Begic H, Nurkic M, Tahirovic H, Varni JW (2010). Does the severity of congenital heart defects affect disease-specific health-related quality of life in children in Bosnia and Herzegovina?. Eur J Pediatr.

[21] Uzark K, Jones K, Burwinkle TM, Varni JW (2003). The pediatric quality of life inventory™ in children with heart disease. Prog Pediatr Cardiol.

[22] Uzark K, Jones K, Slusher J, Limbers CA, Burwinkle TM, Varni JW (2008). Quality of life in children with heart disease as perceived by children and parents. Pediatrics..

[23] Berkes A, Pataki I, Kiss M, Kemeny C, Kardos L, Varni JW (2010). Measuring health-related quality of life in Hungarian children with heart disease: psychometric properties of the Hungarian version of the pediatric quality of life inventory 4.0 generic Core scales and the cardiac module. Health Qual Life Outcomes.

[24] do Nascimento Moraes A, Ramos Ascensao Terreri MT, Esteves Hilario MO, Len CA. (2013). Health related quality of life of children with rheumatic heart diseases: reliability of the Brazilian version of the pediatric quality of life inventory cardiac module scale. Health Qual Life Outcomes.

[25] Gonzalez-Gil T, Mendoza-Soto A, Alonso-Lloret F, Castro-Murga R, Pose-Becerra C, Martin-Arribas MC (2012). The Spanish version of the health-related quality of life questionnaire for children and adolescents with heart disease (PedsQL(TM)). Rev Esp Cardiol (Engl Ed).

[26] Abassi H, Huguet H, Picot MC, Vincenti M, Guillaumont S, Auer A (2020). Health-related quality of life in children with congenital heart disease aged 5 to 7 years: a multicentre controlled cross-sectional study. Health Qual Life Outcomes.

[27] Reiner B, Oberhoffer R, Ewert P, Muller J (2019). Quality of life in young people with congenital heart disease is better than expected. Arch Dis Child.

[28] Loup O, von Weissenfluh C, Gahl B, Schwerzmann M, Carrel T, Kadner A (2009). Quality of life of grown-up congenital heart disease patients after congenital cardiac surgery. Eur J Cardiothorac Surg.

[29] Moreno-Medina K, Barrera-Castaneda M, Vargas-Acevedo C, Garcia-Torres AE, Ronderos M, Huertas-Quinones M (2020). Quality of life in children with infrequent congenital heart defects: cohort study with one-year of follow-up. Health Qual Life Outcomes.

[30] Rein JG, Freed MD, Norwood WI, Castaneda AR (1977). Early and late results of closure of ventricular septal defect in infancy. Ann Thorac Surg.

[31] Yang J, Yang L, Yu S, Liu J, Zuo J, Chen W (2014). Transcatheter versus surgical closure of perimembranous ventricular septal defects in children: a randomized controlled trial. J Am Coll Cardiol.

[32] Yi K, You T, Ding ZH, Hou XD, Liu XG, Wang XK (2018). Comparison of transcatheter closure, mini-invasive closure, and open-heart surgical repair for treatment of perimembranous ventricular septal defects in children: A PRISMA-compliant network meta-analysis of randomized and observational studies. Medicine (Baltimore).

[33] Sarrechia I, De Wolf D, Miatton M, Francois K, Gewillig M, Meyns B (2015). Neurodevelopment and behavior after transcatheter versus surgical closure of secundum type atrial septal defect. J Pediatr.

[34] Wang J, Liu B (2017). Exercise and congenital heart disease. Adv Exp Med Biol.

[35] So SCY, Li WHC, Ho KY (2019). The impact of congenital heart disease on the psychological well-being and quality of life of Hong Kong Chinese adolescents: A cross-sectional study. J Clin Nurs.

