# Supplementary material for: Effects of minimally invasive and traditional surgeries on the quality of life of children with congenital heart disease: a retrospective propensity score-matched study
Source: BMC Pediatr. 2021 Nov 24;21:522. doi: 10.1186/s12887-021-02978-5 (PMC8611858; doi:10.1186/s12887-021-02978-5)
Supplement: Supplementary file 1 — Additional file 1. [file 12887_2021_2978_MOESM1_ESM.pdf]

ID#:__________________________

Date: __________________________

PedsQL™

Cardiac Module

Version 3.0

PARENT REPORT for ADULTS

DIRECTIONS

Adults with heart conditions sometimes have special problems. On the following page is a list of things that might be a problem for **your child**. Please tell us **how much of a problem** each one has been for **your child** during the **past ONE month** by circling:

0 if it is **never** a problem
1 if it is **almost never** a problem
2 if it is **sometimes** a problem
3 if it is **often** a problem
4 if it is **almost always** a problem

There are no right or wrong answers.
If you do not understand a question, please ask for help.

Not to be reproduced without permission
Copyright © 1998 JW Varni, Ph.D.
All rights reserved

In the past ***ONE month,*** *how much of a* ***problem*** *has your child had with …*

**HEART PROBLEMS AND TREATMENT** ***(problems with…)***	**Never**	**Almost Never**	**Some-times**	**Often**	**Almost Always**
1. Getting out of breath while doing sports activity or exercise	0	1	2	3	4
2. Chest pain or tightness while doing sports activity or exercise	0	1	2	3	4
3. Catching colds easily	0	1	2	3	4
4. Fast heartbeat	0	1	2	3	4
5. His/her lips turning blue when running	0	1	2	3	4
6. Waking up at night with trouble breathing	0	1	2	3	4
7. Having to rest more than his/her friends	0	1	2	3	4

If your child is currently taking heart medicine, please answer the following…
Otherwise, please skip to "Perceived Physical Appearance".

**TREATMENT II** ***(problems with…)***	**Never**	**Almost Never**	**Some-times**	**Often**	**Almost Always**
1. Refusing to take heart medicine	0	1	2	3	4
2. Difficulty taking heart medicine	0	1	2	3	4
3. Forgetting to take heart medicine	0	1	2	3	4
4. Heart medicine making him/her feel sick	0	1	2	3	4
5. Worry about side effects from his/her medicine	0	1	2	3	4

**PERCEIVED PHYSICAL APPEARANCE** ***(problems with…)***	**Never**	**Almost Never**	**Some-times**	**Often**	**Almost Always**
1. Feeling that he/she is not good looking	0	1	2	3	4
2. Not liking other people to see his/her scars	0	1	2	3	4
3. Being embarrassed about others seeing his/her body	0	1	2	3	4

**TREATMENT ANXIETY** ***(problems with…)***	**Never**	**Almost Never**	**Some-times**	**Often**	**Almost Always**
1. Getting anxious when waiting to see the doctor	0	1	2	3	4
2. Getting anxious about going to the doctor	0	1	2	3	4
3. Getting anxious about going to the hospital	0	1	2	3	4
4. Getting anxious when he/she has to have medical treatments	0	1	2	3	4

Not to be reproduced without permission

Copyright © 1998 JW Varni, Ph.D.
All rights reserved

*In the past **ONE month,** how much of a **problem** has your child had with …*

**COGNITIVE PROBLEMS *(problems with…)***	**Never**	**Almost Never**	**Some-times**	**Often**	**Almost Always**
1. Figuring out what to do when something bothers him/her	0	1	2	3	4
2. Trouble solving math problems	0	1	2	3	4
3. Trouble writing papers or reports	0	1	2	3	4
4. Difficulty paying attention to things	0	1	2	3	4
5. Remembering what he/she reads	0	1	2	3	4

**COMMUNICATION *(problems with…)***	**Never**	**Almost Never**	**Some-times**	**Often**	**Almost Always**
1. Telling the doctors and nurses how he/she feels	0	1	2	3	4
2. Asking the doctors or nurses questions	0	1	2	3	4
3. Explaining his/her heart problem to other people	0	1	2	3	4

Not to be reproduced without permission
Copyright © 1998 JW Varni, Ph.D.
All rights reserved
