# Supplementary material for: Effects of minimally invasive and traditional surgeries on the quality of life of children with congenital heart disease: a retrospective propensity score-matched study
Source: BMC Pediatr. 2021 Nov 24;21:522. doi: 10.1186/s12887-021-02978-5 (PMC8611858; doi:10.1186/s12887-021-02978-5)
Supplement: Supplementary file 6 — Additional file 6. [file 12887_2021_2978_MOESM6_ESM.pdf]

ID#: ____________________________

Date: ____________________________

PedsQL™

Cardiac Module

Version 3.0

YOUNG ADULT REPORT (ages 18-25)

DIRECTIONS

Young Adults with heart conditions sometimes have special problems. Please tell us **how much of a problem** each one has been for you during the **past ONE month** by circling:

0 if it is **never** a problem
1 if it is **almost never** a problem
2 if it is **sometimes** a problem
3 if it is **often** a problem
4 if it is **almost always** a problem

There are no right or wrong answers.
If you do not understand a question, please ask for help.

Not to be reproduced without permission
Copyright © 1998 JW Varni, Ph.D.
All rights reserved

*In the past **ONE** month, how much of a **problem** has this been for you…*

**HEART PROBLEMS AND TREATMENT** ***(problems with…)***	**Never**	**Almost Never**	**Some-times**	**Often**	**Almost Always**
1. I get out of breath when I do sports activity or exercise	0	1	2	3	4
2. My chest hurts or feels tight when I do sports activity or exercise	0	1	2	3	4
3. I catch colds easily	0	1	2	3	4
4. I feel my heart beating fast	0	1	2	3	4
5. My lips turn blue when I run	0	1	2	3	4
6. I wake up at night with trouble breathing	0	1	2	3	4
7. I have to rest more than my friends	0	1	2	3	4

If you are currently taking heart medicine, please answer the following…
Otherwise, please skip to "Perceived Physical Appearance".

**TREATMENT II** ***(problems with…)***	**Never**	**Almost Never**	**Some-times**	**Often**	**Almost Always**
1. I refuse to take my heart medicine	0	1	2	3	4
2. It is hard for me to take my heart medicine	0	1	2	3	4
3. I forget to take my heart medicine	0	1	2	3	4
4. My heart medicine makes me feel sick	0	1	2	3	4
5. I worry about how my medicines affect my body	0	1	2	3	4

**PERCEIVED PHYSICAL APPEARANCE** ***(problems with…)***	**Never**	**Almost Never**	**Some-times**	**Often**	**Almost Always**
1. I feel I am not good looking	0	1	2	3	4
2. I don't like other people to see my scars	0	1	2	3	4
3. I am embarrassed when others see my body	0	1	2	3	4

**TREATMENT ANXIETY** ***(problems with…)***	**Never**	**Almost Never**	**Some-times**	**Often**	**Almost Always**
1. I get scared when I am waiting to see the doctor	0	1	2	3	4
2. I get scared when I have to go to the doctor	0	1	2	3	4
3. I get scared when I have to go to the hospital	0	1	2	3	4
4. I get scared when I have to have medical treatments	0	1	2	3	4

Not to be reproduced without permission
Copyright © 1998 JW Varni, Ph.D.
All rights reserved

*In the past **ONE** month, how much of a **problem** has this been for you…*

COGNITIVE PROBLEMS ***(problems with…)***	**Never**	**Almost Never**	**Some-times**	**Often**	**Almost Always**
1. It is hard for me to figure out what to do when something bothers me	0	1	2	3	4
2. I have trouble solving math problems	0	1	2	3	4
3. I have trouble writing papers or reports	0	1	2	3	4
4. It is hard for me to pay attention to things	0	1	2	3	4
5. It is hard for me to remember what I read	0	1	2	3	4

COMMUNICATION ***(problems with…)***	**Never**	**Almost Never**	**Some-times**	**Often**	**Almost Always**
1. It is hard for me to tell the doctors and nurses how I feel	0	1	2	3	4
2. It is hard for me to ask the doctors and nurses questions	0	1	2	3	4
3. It is hard for me to explain my heart problem to other people	0	1	2	3	4

Not to be reproduced without permission

Copyright © 1998 JW Varni, Ph.D.
All rights reserved
