# Supplementary material for: Effects of minimally invasive and traditional surgeries on the quality of life of children with congenital heart disease: a retrospective propensity score-matched study
Source: BMC Pediatr. 2021 Nov 24;21:522. doi: 10.1186/s12887-021-02978-5 (PMC8611858; doi:10.1186/s12887-021-02978-5)
Supplement: Supplementary file 7 — Additional file 7. [file 12887_2021_2978_MOESM7_ESM.pdf]

ID# ____________________________

Date:__________________________

PedsQL ™

Cardiac Module

Version 3.0

YOUNG CHILD REPORT (ages **5-7**)

Instructions for interviewer:

I am going to ask you some questions about things that might be a problem for some children. I want to know how much of a problem any of these things might be for you.

Show the child the template and point to the responses as you read**.**

If it is <u>not at all</u> a problem for you, point to the smiling face

If it is <u>sometimes</u> a problem for you, point to the middle face

If it is a problem for you <u>a lot</u>, point to the frowning face

I will read each question. Point to the pictures to show me how much of a problem it is for you. Let's try a practice one first.

	Not at all	Sometimes	A lot
**Is it hard for you to snap your fingers**	☺	😐	☹

Ask the child to demonstrate snapping his or her fingers to determine whether or not the question was answered correctly. Repeat the question if the child demonstrates a response that is different from his or her action.

Not to be reproduced without permission

Copyright © 1998 JW Varni, Ph.D.
All rights reserved

Think about how you have been doing for the last few weeks. Please listen carefully to each sentence and tell me how much of a problem this is for you.

After reading the item, gesture to the template. If the child hesitates or does not seem to understand how to answer, read the response options while pointing at the faces.

**HEART PROBLEMS AND TREATMENT** ***(problems with…)***	**Not at all**	**Some-times**	**A lot**
1. Is it hard for you to breathe when you do sports activity or exercise	0	2	4
2. Does your chest hurt when you do sports activity or exercise	0	2	4
3. Do you catch colds more than other kids	0	2	4
4. Do you feel your heart beating fast	0	2	4
5. Do others tell you that your lips turn blue when you run	0	2	4
6. Do you wake up at night with trouble breathing	0	2	4
7. Do you have to rest more than your friends	0	2	4

If you are currently taking heart medicine, please answer the following…
Otherwise, please skip to "Perceived Physical Appearance".

**TREATMENT II** ***(problems with…)***	**Not at all**	**Some-times**	**A lot**
1. Do you say no to taking your heart medicine	0	2	4
2. Do you have a hard time taking your heart medicine	0	2	4
3. Does your heart medicine make you feel sick	0	2	4

**PERCEIVED PHYSICAL APPEARANCE** ***(problems with…)***	**Not at all**	**Some-times**	**A lot**
1. Do you feel you don't look good	0	2	4
2. Do you not like other people to see your scars	0	2	4
3. Do other kids tease you when they see your scars	0	2	4

**TREATMENT ANXIETY** ***(problems with…)***	**Not at all**	**Some-times**	**A lot**
1. Do you get scared when you are waiting to see the doctor	0	2	4
2. Do you get scared when you have to go to the doctor	0	2	4
3. Do you get scared when you have to go to the hospital	0	2	4
4. Do you get scared when you have to have heart tests	0	2	4

Not to be reproduced without permission
Copyright © 1998 JW Varni, Ph.D.
All rights reserved

Think about how you have been doing for the last few weeks. Please listen carefully to each sentence and tell me how much of a problem this is for you.

**COGNITIVE PROBLEMS** ***(problems with…)***	**Not at all**	**Some-times**	**A lot**
1. Is it hard for you to know what to do when something bothers you	0	2	4
2. Do you have trouble with numbers or math worksheets	0	2	4
3. Do you have trouble writing letters or words	0	2	4
4. Is it hard for you to listen to the teacher	0	2	4
5. Is it hard for you to remember what is read to you	0	2	4

**COMMUNICATION** ***(problems with…)***	**Not at all**	**Some-times**	**A lot**
1. Is it hard for you to tell the doctors and nurses how you feel	0	2	4
2. Is it hard for you to ask the doctors and nurses questions	0	2	4
3. Is it hard for you to explain your heart problem to other people	0	2	4

Not to be reproduced without permission
Copyright © 1998 JW Varni, Ph.D.
All rights reserved

How much of a problem is this for you?

Not at all	Sometimes	A lot
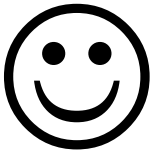	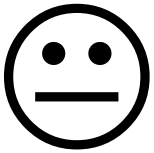	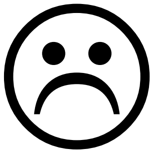

Copyright © 1998 JW Varni, Ph.D. All rights reserved
