# Supplementary material for: Effects of minimally invasive and traditional surgeries on the quality of life of children with congenital heart disease: a retrospective propensity score-matched study
Source: BMC Pediatr. 2021 Nov 24;21:522. doi: 10.1186/s12887-021-02978-5 (PMC8611858; doi:10.1186/s12887-021-02978-5)
Supplement: Supplementary file 8 — Additional file 8. [file 12887_2021_2978_MOESM8_ESM.pdf]

SCALING AND SCORING OF THE

Pediatric Quality of Life Inventory™

PedsQL™

Mapi Research Trust
27 rue de la Villette
69003 Lyon
France
Phone: +33 (0) 4 72 13 66 66
Submit your request directly on our platform:
https://eprovide.mapi-trust.org/

James W. Varni, Ph.D.
Professor Emeritus
Department of Pediatrics, College of Medicine
Department of Landscape Architecture and Urban Planning
College of Architecture
3137 TAMU
College Station, Texas 77843-3137
USA
Email: jvarni@tamu.edu

PedsQL ™ 4.0 Generic Core Scales ... 5
PARENT Report for Toddlers (ages 2-4) ... 5
CHILD and PARENT Reports for Young Children (ages 5-7), Children (ages 8-12), Teens (ages 13-18) ... 7
YOUNG ADULT and PARENT Report for Young Adult (ages 18-25), ADULT and PARENT Report for Adult (ages over 26) ... 9
PedsQL ™ 4.0 SF15 Generic Core Scales ... 11
PARENT Report for Toddlers (ages 2-4), CHILD and PARENT Reports for Young Children (ages 5-7), Children (ages 8-12), Teens (ages 13-18), Young Adults (ages 18-25) and Adults (ages over 26) ... 11
PedsQL ™ 3.0 Arthritis Module ... 14
PARENT Report for Toddlers (ages 2-4) ... 14
CHILD and PARENT Reports for Young Children (ages 5-7) ... 16
CHILD and PARENT Reports for Children (ages 8-12), Teens (ages 13-18) ... 18
PedsQL ™ 3.0 Asthma Module ... 20
PARENT Report for Toddlers (ages 2-4) ... 20
CHILD and PARENT Reports for Young Children (ages 5-7), Children (ages 8-12), Teens (ages 13-18) ... 22
PedsQL ™ 3.0 SF22 Asthma Module ... 24
PARENT Report for Toddlers (ages 2-4) ... 24
CHILD and PARENT Reports for Young Children (ages 5-7), Children (ages 8-12), Teens (ages 13-18) ... 26
PedsQL ™ Brain Tumor Module ... 28
PARENT Report for Toddlers (ages 2-4) ... 28
CHILD and PARENT Reports for Young Children (ages 5-7) ... 30
CHILD and PARENT Reports for Children (ages 8-12), Teens (ages 13-18) ... 32
PedsQL ™ 3.0 Cancer Module ... 34
PARENT Report for Toddlers (ages 2-4) ... 34
CHILD and PARENT Reports for Young Children (ages 5-7) ... 36
CHILD and PARENT Reports for Children (ages 8-12), Teens (ages 13-18), Young Adults (ages 18-25), and ADULT Report (ages over 26) ... 38
PedsQL ™ 3.0 Cardiac Module ... 40
PARENT Report for Toddlers (ages 2-4) ... 40
CHILD and PARENT Reports for Young Children (ages 5-7) ... 42
CHILD and PARENT Reports for Children (ages 8-12), Teens (ages 13-18), Young Adults (ages 18-25), and Adults (ages over 26) ... 44
PedsQL ™ 3.0 Cerebral Palsy Module ... 46
PARENT Report for Toddlers (ages 2-4) ... 46
CHILD and PARENT Reports for Young Children (ages 5-7), Children (ages 8-12), Teens (ages 13-18) ... 48
PedsQL ™ Cognitive Functioning Scale ... 50
PedsQL ™ 3.0 Diabetes Module ... 52
PedsQL ™ 3.2 Diabetes Module ... 54
PedsQL ™ 3.0 Duchenne Muscular Dystrophy Module ... 55
PARENT Report for Young Children (ages 5-7) ... 57
CHILD and PARENT Reports for Children (ages 8-12) and Teens (ages 13-18) ... 59
PedsQL ™ 3.0 End Stage Renal Disease Module ... 61
PARENT Report for Toddlers (ages 2-4) ... 61

CHILD and PARENT Reports for Young Children (ages 5-7), Children (ages 8-12), Teens (ages 13-18), and Young Adults (ages 18-25)....63
PedsQL ™ 3.0 Eosinophilic Esophagitis Module....65
PARENT Report for Toddlers (ages 2-4)....65
CHILD and PARENT Reports for Young Children (ages 5-7)....67
CHILD and PARENT Reports for Children (ages 8-12) and Teens (ages 13-18)....70
PedsQL ™ Eosinophilic Esophagitis Symptoms Scales....72
PARENT Report for Toddlers (ages 2-4)....72
PARENT Reports for Young Children (ages 5-7)....74
CHILD and PARENT Reports for Children (ages 8-12) and Teens (ages 13-18)....76
PedsQL ™ 3.0 Epilepsy Module....78
PARENT Report for Toddlers (ages 2-4)....78
CHILD Reports for Young Children (ages 5-7)....80
CHILD and PARENT Reports for Children (ages 5-7), Children (ages 8-12), Teens (ages 13-18), Young Adults (ages 18-25)....**Error! Bookmark not defined.**
PedsQL TM 2.0 Family Impact Module....86
PARENT Report....87
PedsQL ™ 3.0 Gastrointestinal Symptoms Module....90
PARENT Report for Toddlers (ages 2-4)....90
CHILD and PARENT Report for Young Children (ages 5-7)....93
CHILD and PARENT Reports for Children (ages 8-12), Teens (ages 13-18) and Young Adults (18-25)....96
PedsQL ™ 3.0 Gastrointestinal Symptoms Scales™....99
PARENT Report for Toddlers (ages 2-4)....99
CHILD and PARENT Reports for Young Children (ages 5-7)....101
CHILD and PARENT Reports for Children (ages 8-12), Teens (ages 13-18) and Young Adults (ages 18-25)....103
PedsQL ™ General Well-Being Scale....105
CHILD, ADOLESCENT and YOUNG ADULT Report (ages 8-25 years of age)....105
PedsQL™ Healthcare Satisfaction Generic Module....107
PedsQL™ Healthcare Satisfaction Hematology / Oncology Module....109
PedsQL ™ Infant Scales....111
PARENT Report for Infants (ages 1-24 months)....111
PedsQL ™ Multidimensional Fatigue Scale....114
PedsQL ™ 3.0 Neurofibromatosis Module....116
CHILD and PARENT Reports for Young Children (ages 5-7), Children (ages 8-12), Teens (ages 13-18), Young Adults (ages 18-25), and Adults (ages over 26)....116
PedsQL ™ 3.0 Neuromuscular Module....119
PedsQL ™ Oral Health Scale....121
PedsQL ™ Pediatric Pain Coping Inventory™ (PPCI)....123
PARENT Report for Children and Teens (ages 5-18), CHILD Report for Children (ages 5-12), Teens (ages 13-18)....123
PedsQL ™ Pediatric Pain Questionnaire ™ (PPQ)....125
PedsQL ™ Present Functioning Visual Analogue Scale (PedsQL ™ VAS)....127
CHILD and PARENT reports for CHILD (ages 5-18)....127
PedsQL ™ 3.0 Rheumatology Module....129
PARENT Report for Toddlers (ages 2-4)....129
CHILD and PARENT reports for Young Children (ages 5-7)....131
CHILD and PARENT reports for Children (ages 8-12) and Teens (ages 13-18)....133

PedsQL ™ 3.0 Sickle Cell Disease Module **135**
PARENT Report for Toddlers (ages 2-4) 135
CHILD and PARENT Reports for Young Children (ages 5-7) 137
CHILD and PARENT Reports for Children (ages 8-12),Teens (ages 13-18), Young Adults (ages 18-25), and Adults (ages over 26) 139
PedsQL ™ 1.0 Stem Cell Transplant Module **141**
PARENT Report for Toddlers (ages 2-4) 141
PARENT Report for Young Children (ages 5-7) 143
CHILD and PARENT Reports for Children (ages 8-12) 145
TEEN and PARENT Reports for Teens (ages 13-18) 147
PedsQL ™ 3.0 Transplant Module **149**

PedsQL TM 4.0 Generic Core Scales

PARENT Report for Toddlers (ages 2-4)

The Parent Report for Toddlers (ages 2-4) of the **PedsQL™** 4.0 Generic Core Scales is composed of 21 items comprising 4 dimensions.

DESCRIPTION OF THE QUESTIONNAIRE:

Dimensions	Number of Items	Cluster of Items	Reversed Scoring	Direction of Dimensions
Physical Functioning	8	1-8	1-8	Higher scores indicate better HRQOL.
Emotional Functioning	5	1-5	1-5	
Social Functioning	5	1-5	1-5	
School Functioning	3	1-3	1-3	

SCORING OF DIMENSIONS:

**Item Scaling**	5-point Likert scale from 0 (Never) to 4 (Almost always)
**Weighting of Items**	No
**Extension of the Scoring Scale**	Scores are transformed on a scale from 0 to 100.
**Scoring Procedure**	**Step 1: Transform Score**<br>Items are reversed scored and linearly transformed to a 0-100 scale as follows: 0=100, 1=75, 2=50, 3=25, 4=0.<br>**Step 2: Calculate Scores**<br>Score by Dimensions:<br>• If more than 50% of the items in the scale are missing, the scale scores should not be computed.<br>• Mean score = Sum of the items over the number of items answered.<br>Psychosocial Health Summary Score = Sum of the items over the number of items answered in the Emotional, Social, and School Functioning Scales.<br>Physical Health Summary Score = Physical Functioning Scale Score<br>**Total Score**: Sum of all the items over the number of items answered on all the Scales.
**Interpretation and Analysis of Missing Data**	If more than 50% of the items in the scale are missing, the Scale Scores should not be computed.<br>If 50% or more items are completed: Impute the mean of the completed items in a scale.

CHILD and PARENT Reports for Young Children (ages 5-7), Children (ages 8-12), Teens (ages 13-18)

The **Child and Parent Reports** of the **PedsQL^{TM}** 4.0 Generic Core Scales for:

- Young Children (ages 5-7),
- Children (ages 8-12),
- And Teens (ages 13-18),

are composed of 23 items comprising 4 dimensions.

DESCRIPTION OF THE QUESTIONNAIRE:

Dimensions	Number of Items	Cluster of Items	Reversed scoring	Direction of Dimensions
Physical Functioning	8	1-8	1-8	Higher scores indicate better HRQOL.
Emotional Functioning	5	1-5	1-5	
Social Functioning	5	1-5	1-5	
School Functioning	5	1-5	1-5	

SCORING OF DIMENSIONS:

**Item Scaling**	5-point Likert scale from 0 (Never) to 4 (Almost always)<br>3-point scale: 0 (Not at all), 2 (Sometimes) and 4 (A lot) for the Young Child (ages 5-7) child report
**Weighting of Items**	No
**Extension of the Scoring Scale**	Scores are transformed on a scale from 0 to 100.
**Scoring Procedure**	**Step 1: Transform Score**<br>Items are reversed scored and linearly transformed to a 0-100 scale as follows: 0=100, 1=75, 2=50, 3=25, 4=0.<br>**Step 2: Calculate Scores**<br>Score by Dimensions:<br>• If more than 50% of the items in the scale are missing, the scale scores should not be computed,<br>• Mean score = Sum of the items over the number of items answered.<br>Psychosocial Health Summary Score = Sum of the items over the number of items answered in the Emotional, Social, and School Functioning Scales.<br>Physical Health Summary Score = Physical Functioning Scale Score<br>**Total Score**: Sum of all the items over the number of items answered on all the Scales.
**Interpretation and Analysis of Missing Data**	If more than 50% of the items in the scale are missing, the Scale Scores should not be computed.<br>If 50% or more items are completed: Impute the mean of the completed items in a scale.

YOUNG ADULT and PARENT Report for Young Adult (ages 18-25),
ADULT and PARENT Report for Adult (ages over 26)

The Parent, **Young Adult and Adult Reports** of the **PedsQL™** 4.0 Generic Core Scales for:

- Young Adults (ages 18-25),
- Adults (ages over 26),

are composed of 23 items comprising 4 dimensions.

DESCRIPTION OF THE QUESTIONNAIRE:

Dimensions	Number of Items	Cluster of Items	Reversed scoring	Direction of Dimensions
Physical Functioning	8	1-8	1-8	Higher scores indicate better HRQOL.
Emotional Functioning	5	1-5	1-5	
Social Functioning	5	1-5	1-5	
School Functioning	5	1-5	1-5	

SCORING OF DIMENSIONS:

**Item Scaling**	5-point Likert scale from 0 (Never) to 4 (Almost always)
**Weighting of Items**	No
**Extension of the Scoring Scale**	Scores are transformed on a scale from 0 to 100.
**Scoring Procedure**	**Step 1: Transform Score**<br>Items are reversed scored and linearly transformed to a 0-100 scale as follows: 0=100, 1=75, 2=50, 3=25, 4=0.<br><br>**Step 2: Calculate Scores**<br>Score by Dimensions:<br>• If more than 50% of the items in the scale are missing, the scale scores should not be computed,<br>• Mean score = Sum of the items over the number of items answered.<br><br>Psychosocial Health Summary Score = Sum of the items over the number of items answered in the Emotional, Social, and School Functioning Scales.<br><br>Physical Health Summary Score = Physical Functioning Scale Score<br><br>**Total Score**: Sum of all the items over the number of items answered on all the Scales.
**Interpretation and Analysis of Missing Data**	If more than 50% of the items in the scale are missing, the Scale Scores should not be computed.<br>If 50% or more items are completed: Impute the mean of the completed items in a scale.

PedsQL ^{TM} 4.0 SF15 Generic Core Scales

PARENT Report for Toddlers (ages 2-4),
CHILD and PARENT Reports for Young Children (ages 5-7),
Children (ages 8-12), Teens (ages 13-18), Young Adults (ages 18-25) and Adults (ages over 26)

The Child and Parent Reports of the PedsQL™ **4.0 SF15 Generic Core Scales** for

- Toddlers (ages 2-4),
- Young Children (ages 5-7)
- Children (ages 8-12),
- Teens (ages 13-18),
- Young Adults (ages 18-25),
- And Adults (ages over 26)

are composed of 15 items comprising 4 dimensions.

DESCRIPTION OF THE SF15 QUESTIONNAIRE:

Dimensions	Number of Items	Cluster of Items	Reversed Scoring	Direction of Dimensions
Physical Functioning	5	1-5	1-5	Higher scores indicate better HRQOL.
Emotional Functioning	4	1-4	1-4	
Social Functioning	3	1-3	1-3	
School Functioning	3	1-3	1-3	

SCORING OF DIMENSIONS:

**Item Scaling**	5-point Likert scale from 0 (Never) to 4 (Almost always)<br>3-point scales: 0 (Not at all), 2 (Sometimes) and 4 (A lot) for the Young Child self-report
**Weighting of Items**	No
**Extension of the Scoring Scale**	Scores are transformed on a scale from 0 to 100.
**Scoring Procedure**	**Step 1: Transform Score**<br>Items are reversed scored and linearly transformed to a 0-100 scale as follows: 0=100, 1=75, 2=50, 3=25, 4=0.<br>**Step 2: Calculate Scores**<br>Score by Dimensions:<br>• If more than 50% of the items in the scale are missing, the scale scores should not be computed,<br>• Mean score = Sum of the items over the number of items answered.<br>Psychosocial Health Summary Score = Sum of the items over the number of items answered in the Emotional, Social, and School Functioning Scales.<br>Physical Health Summary Score = Physical Functioning Scale Score

	**Total Score**: Sum of all the items over the number of items answered on all the Scales.
**Interpretation and Analysis of Missing Data**	If more than 50% of the items in the scale are missing, the Scale Scores should not be computed.<br><br>If 50% or more items are completed: Impute the mean of the completed items in a scale.

PedsQL TM 3.0 Arthritis Module

PARENT Report for Toddlers (ages 2-4)

The Parent Report for Toddlers **(ages 2-4) of the** PedsQL TM **3.0 Arthritis Module is composed of 14 items comprising 3 dimensions.**

DESCRIPTION OF THE ARTHRITIS MODULE:

Dimensions	Number of Items	Cluster of Items	Reversed Scoring	Direction of Dimensions
Pain and Hurt	4	1-4	1-4	Higher scores indicate lower problems.
Daily Activities	5	1-5	1-5	
Treatment	5	1-5	1-5	

SCORING OF DIMENSIONS:

**Item Scaling**	5-point Likert scale from 0 (Never) to 4 (Almost always)
**Weighting of Items**	No
**Extension of the Scoring Scale**	Scores are transformed on a scale from 0 to 100.
**Scoring Procedure**	**Step 1: Transform Score**<br>Items are reversed scored and linearly transformed to a 0-100 scale as follows: 0=100, 1=75, 2=50, 3=25, 4=0.<br>**Step 2: Calculate Scores by Dimensions**<br>• If more than 50% of the items in the scale are missing, the scale scores should not be computed<br>• Mean score = Sum of the items over the number of items answered<br>**There is no Total Score**
**Interpretation and Analysis of Missing Data**	If more than 50% of the items in the scale are missing, the Scale Scores should not be computed.<br>If 50% or more items are completed: Impute the mean of the completed items in a scale.

CHILD and PARENT Reports for Young Children (ages 5-7)

The Child and Parent Reports **of the** PedsQL TM **3.0 Arthritis Module for** Young Children **(ages 5-7) are composed of 20 items comprising 5 dimensions.**

DESCRIPTION OF THE ARTHRITIS MODULE:

Dimensions	Number of Items	Cluster of Items	Reversed Scoring	Direction of Dimensions
Pain and Hurt	4	1-4	1-4	Higher scores indicate lower problems.
Daily Activities	5	1-5	1-5	
Treatment	5	1-5	1-5	
Worry	3	1-3	1-3	
Communication	3	1-3	1-3	

SCORING OF DIMENSIONS:

**Item Scaling**	5-point Likert scale from 0 (Never) to 4 (Almost always) for the parent report<br>3-point scale: 0 (Not at all), 2 (Sometimes) and 4 (A lot) for the child report
**Weighting of Items**	No
**Extension of the Scoring Scale**	Scores are transformed on a scale from 0 to 100.
**Scoring Procedure**	**Step 1: Transform Score**<br>Items are reversed scored and linearly transformed to a 0-100 scale as follows: 0=100, 1=75, 2=50, 3=25, 4=0.<br>**Step 2: Calculate Scores by Dimensions**<br>• If more than 50% of the items in the scale are missing, the scale scores should not be computed,<br>• Mean score = Sum of the items over the number of items answered.<br>**There is no Total Score**
**Interpretation and Analysis of Missing Data**	If more than 50% of the items in the scale are missing, the Scale Scores should not be computed.<br>If 50% or more items are completed: Impute the mean of the completed items in a scale.

CHILD and PARENT Reports for Children (ages 8-12), Teens (ages 13-18)

The **Child and Parent Reports** of the **PedsQL** TM 3.0 Arthritis Module for:
- Children (ages 8-12),
- And Teens (ages 13-18),

are composed of 22 items comprising 5 dimensions.

DESCRIPTION OF THE ARTHRITIS MODULE:

Dimensions	Number of Items	Cluster of Items	Reversed Scoring	Direction of Dimensions
Pain and Hurt	4	1-4	1-4	Higher scores indicate lower problems.
Daily Activities	5	1-5	1-5	
Treatment	7	1-7	1-7	
Worry	3	1-3	1-3	
Communication	3	1-3	1-3	

SCORING OF DIMENSIONS:

**Item Scaling**	5-point Likert scale from 0 (Never) to 4 (Almost always)
**Weighting of Items**	No
**Extension of the Scoring Scale**	Scores are transformed on a scale from 0 to 100.
**Scoring Procedure**	**Step 1: Transform Score**<br>Items are reversed scored and linearly transformed to a 0-100 scale as follows: 0=100, 1=75, 2=50, 3=25, 4=0.<br><br>**Step 2: Calculate Scores by Dimensions**<br>• If more than 50% of the items in the scale are missing, the scale scores should not be computed,<br>• Mean score = Sum of the items over the number of items answered.<br><br>**There is no Total Score**
**Interpretation and Analysis of missing data**	If more than 50% of the items in the scale are missing, the Scale Scores should not be computed.<br>If 50% or more items are completed: Impute the mean of the completed items in a scale.

PedsQL ™ 3.0 Asthma Module

PARENT Report for Toddlers (ages 2-4)

The Parent Report for Toddlers **(ages 2-4) of the** PedsQL TM **3.0 Asthma Module is composed of 26 items comprising 4 dimensions.**

DESCRIPTION OF THE ASTHMA MODULE:

Dimensions	Number of Items	Cluster of Items	Reversed Scoring	Direction of Dimensions
Asthma	11	1-11	1-11	Higher scores indicate lower problems.
Treatment	9	1-9	1-9	
Worry	3	1-3	1-3	
Communication	3	1-3	1-3	

SCORING OF DIMENSIONS:

**Item Scaling**	5-point Likert scale from 0 (Never) to 4 (Almost always)
**Weighting of Items**	No
**Extension of the Scoring Scale**	Scores are transformed on a scale from 0 to 100.
**Scoring Procedure**	**Step 1: Transform Score**<br>Items are reversed scored and linearly transformed to a 0-100 scale as follows: 0=100, 1=75, 2=50, 3=25, 4=0.<br>**Step 2: Calculate Scores by Dimensions**<br>• If more than 50% of the items in the scale are missing, the scale scores should not be computed,<br>• Mean score = Sum of the items over the number of items answered.<br>**There is no Total Score**
**Interpretation and Analysis of Missing Data**	If more than 50% of the items in the scale are missing, the Scale Scores should not be computed.<br>If 50% or more items are completed: Impute the mean of the completed items in a scale.

CHILD and PARENT Reports for Young Children (ages 5-7), Children (ages 8-12), Teens (ages 13-18)

The Child and Parent Reports **of the** PedsQL TM **3.0 Asthma Module for:**

- Young Children (ages 5-7),
- Children (ages 8-12),
- And Teens (ages 13-18),

are composed of 28 items comprising 4 dimensions.

DESCRIPTION OF THE ASTHMA MODULE:

Dimensions	Number of Items	Cluster of Items	Reversed Scoring	Direction of Dimensions
Asthma	11	1-11	1-11	Higher scores indicate lower problems.
Treatment	11	1-11	1-11	
Worry	3	1-3	1-3	
Communication	3	1-3	1-3	

SCORING OF DIMENSIONS:

**Item Scaling**	5-point Likert scale from 0 (Never) to 4 (Almost always)<br>3-point scale: 0 (Not at all), 2 (Sometimes) and 4 (A lot) for the child report for Young Children (ages 5-7)
**Weighting of Items**	No
**Extension of the Scoring Scale**	Scores are transformed on a scale from 0 to 100.
**Scoring Procedure**	**Step 1: Transform Score**<br>Items are reversed scored and linearly transformed to a 0-100 scale as follows: 0=100, 1=75, 2=50, 3=25, 4=0.<br>**Step 2: Calculate Scores by Dimensions**<br>• If more than 50% of the items in the scale are missing, the scale scores should not be computed,<br>• Mean score = Sum of the items over the number of items answered.<br>**There is no Total Score**
**Interpretation and Analysis of Missing Data**	If more than 50% of the items in the scale are missing, the Scale Scores should not be computed.<br>If 50% or more items are completed: Impute the mean of the completed items in a scale.

PedsQL TM 3.0 SF22 Asthma Module

PARENT Report for Toddlers (ages 2-4)

The Parent Report for Toddlers **(ages 2-4) of the** PedsQL TM **3.0 SF22 Asthma Module is composed of 20 items comprising 2 dimensions.**

DESCRIPTION OF THE SF22 ASTHMA MODULE:

Dimensions	Number of Items	Cluster of Items	Reversed Scoring	Direction of Dimensions
Asthma	11	1-11	1-11	Higher scores indicate lower problems.
Treatment	9	1-9	1-9	

SCORING OF DIMENSIONS:

**Item Scaling**	5-point Likert scale from 0 (Never) to 4 (Almost always)
**Weighting of Items**	No
**Extension of the Scoring Scale**	Scores are transformed on a scale from 0 to 100.
**Scoring Procedure**	**Step 1: Transform Score**<br>Items are reversed scored and linearly transformed to a 0-100 scale as follows: 0=100, 1=75, 2=50, 3=25, 4=0.<br><br>**Step 2: Calculate Scores by Dimensions**<br>• If more than 50% of the items in the scale are missing, the scale scores should not be computed,<br>• Mean score = Sum of the items over the number of items answered.<br><br>**There is no Total Score**
**Interpretation and Analysis of Missing Data**	If more than 50% of the items in the scale are missing, the Scale Scores should not be computed.<br>If 50% or more items are completed: Impute the mean of the completed items in a scale.

CHILD and PARENT Reports for Young Children (ages 5-7), Children (ages 8-12), Teens (ages 13-18)

The Child and Parent Reports **of the** PedsQL™ **3.0 SF22 Asthma Module for:**

- Young Children (ages 5-7),
- Children (ages 8-12),
- And Teens (ages 13-18)

are composed of 22 items comprising 2 dimensions.

DESCRIPTION OF THE SF22 ASTHMA MODULE:

Dimensions	Number of Items	Cluster of Items	Reversed Scoring	Direction of Dimensions
Asthma	11	1-11	1-11	Higher scores indicate lower problems.
Treatment	11	1-11	1-11	

SCORING OF DIMENSIONS:

**Item Scaling**	5-point Likert scale from 0 (Never) to 4 (Almost always)<br>3-point scale: 0 (Not at all), 2 (Sometimes) and 4 (A lot) for the Child R Report for Young Children (ages 5-7)
**Weighting of Items**	No
**Extension of the Scoring Scale**	Scores are transformed on a scale from 0 to 100.
**Scoring Procedure**	**Step 1: Transform Score**<br>Items are reversed scored and linearly transformed to a 0-100 scale as follows: 0=100, 1=75, 2=50, 3=25, 4=0.<br>**Step 2: Calculate Scores by Dimensions**<br>• If more than 50% of the items in the scale are missing, the scale scores should not be computed,<br>• Mean score = Sum of the items over the number of items answered.<br>**There is no Total Score**
**Interpretation and Analysis of Missing Data**	If more than 50% of the items in the scale are missing, the Scale Scores should not be computed.<br>If 50% or more items are completed: Impute the mean of the completed items in a scale.

PedsQL ™ Brain Tumor Module

PARENT Report for Toddlers (ages 2-4)

The Parent Report for Toddlers **(ages 2-4) of the** PedsQL™ **Brain Tumor Module is composed of 17 items comprising 5 dimensions.**

DESCRIPTION OF THE BRAIN TUMOR MODULE:

Dimensions	Number of Items	Cluster of Items	Reversed Scoring	Direction of Dimensions
Pain and Hurt	3	1-3	1-3	Higher scores indicate lower problems.
Movement and Balance	3	1-3	1-3	
Procedural Anxiety	3	1-3	1-3	
Nausea	5	1-5	1-5	
Worry	3	1-3	1-3	

SCORING OF DIMENSIONS:

**Item Scaling**	5-point Likert scale from 0 (Never a problem) to 4 (Almost always a problem)
**Weighting of Items**	No
**Extension of the Scoring Scale**	Scores are transformed on a scale from 0 to 100.
**Scoring Procedure**	**Step 1: Transform Score**<br>Items are reversed scored and linearly transformed to a 0-100 scale as follows: 0=100, 1=75, 2=50, 3=25, 4=0.<br>**Step 2: Calculate Scores by Dimensions**<br>• If more than 50% of the items in the scale are missing, the scale scores should not be computed,<br>• Mean score = Sum of the items over the number of items answered.<br>**There is no Total Score**
**Interpretation and Analysis of Missing Data**	If more than 50% of the items in the scale are missing, the Scale Scores should not be computed.<br>If 50% or more items are completed: Impute the mean of the completed items in a scale.

CHILD and PARENT Reports for Young Children (ages 5-7)

The **Parent and Child Reports for Young Children** (ages 5-7) of the **PedsQL ™** Brain Tumor Module are composed of 23 items comprising 6 dimensions.

DESCRIPTION OF THE BRAIN TUMOR MODULE:

Dimensions	Number of Items	Cluster of Items	Reversed Scoring	Direction of Dimensions
Cognitive Problems	6	1-6	1-6	Higher scores indicate lower problems.
Pain and Hurt	3	1-3	1-3	
Movement and Balance	3	1-3	1-3	
Procedural Anxiety	3	1-3	1-3	
Nausea	5	1-5	1-5	
Worry	3	1-3	1-3	

SCORING OF DIMENSIONS:

**Item Scaling**	5-point Likert scale from 0 (Never) to 4 (Almost always)<br>3-point scale: 0 (Not at all), 2 (Sometimes) and 4 (A lot) for the Child Report for Young Children (ages 5-7)
**Weighting of Items**	No
**Extension of the Scoring Scale**	Scores are transformed on a scale from 0 to 100.
**Scoring Procedure**	**Step 1: Transform Score**<br>Items are reversed scored and linearly transformed to a 0-100 scale as follows: 0=100, 1=75, 2=50, 3=25, 4=0.<br>**Step 2: Calculate Scores by Dimensions**<br>• If more than 50% of the items in the scale are missing, the scale scores should not be computed,<br>• Mean score = Sum of the items over the number of items answered.<br>**There is no Total Score**
**Interpretation and Analysis of Missing Data**	If more than 50% of the items in the scale are missing, the Scale Scores should not be computed.<br>If 50% or more items are completed: Impute the mean of the completed items in a scale.

CHILD and PARENT Reports for Children (ages 8-12), Teens (ages 13-18)

The **Parent and Child Reports for Children** (ages 8-12) and **Teens** (ages 13-18) of the **PedsQL[TM]** Brain Tumor Module are composed of 24 items comprising 6 dimensions.

DESCRIPTION OF THE BRAIN TUMOR MODULE:

Dimensions	Number of Items	Cluster of Items	Reversed Scoring	Direction of Dimensions
Cognitive Problems	7	1-7	1-7	Higher scores indicate lower problems
Pain and Hurt	3	1-3	1-3	
Movement and Balance	3	1-3	1-3	
Procedural Anxiety	3	1-3	1-3	
Nausea	5	1-5	1-5	
Worry	3	1-3	1-3	

SCORING OF DIMENSIONS:

**Item Scaling**	5-point Likert scale from 0 (Never a problem) to 4 (Almost always a problem)
**Weighting of Items**	No
**Extension of the Scoring Scale**	Scores are transformed on a scale from 0 to 100.
**Scoring Procedure**	**Step 1: Transform Score**<br>Items are reversed scored and linearly transformed to a 0-100 scale as follows: 0=100, 1=75, 2=50, 3=25, 4=0.<br><br>**Step 2: Calculate Scores by Dimensions**<br>• If more than 50% of the items in the scale are missing, the scale scores should not be computed,<br>• Mean score = Sum of the items over the number of items answered.<br><br>**There is no Total Score**
**Interpretation and Analysis of Missing Data**	If more than 50% of the items in the scale are missing, the Scale Scores should not be computed.<br>If 50% or more items are completed: Impute the mean of the completed items in a scale.

PedsQL ™ 3.0 Cancer Module

PARENT Report for Toddlers (ages 2-4)

The Parent Report for Toddlers (ages 2-4) of the **PedsQL™** 3.0 Cancer Module is composed of 25 items comprising 8 dimensions.

DESCRIPTION OF THE CANCER MODULE:

Dimensions	Number of Items	Cluster of Items	Reversed Scoring	Direction of Dimensions
Pain and Hurt	2	1-2	1-2	Higher scores indicate lower problems.
Nausea	5	1-5	1-5	
Procedural Anxiety	3	1-3	1-3	
Treatment Anxiety	3	1-3	1-3	
Worry	3	1-3	1-3	
Cognitive Problems	3	1-3	1-3	
Perceived Physical Appearance	3	1-3	1-3	
Communication	3	1-3	1-3	

SCORING OF DIMENSIONS:

**Item Scaling**	5-point Likert scale from 0 (Never) to 4 (Almost always)
**Weighting of Items**	No
**Extension of the Scoring Scale**	Scores are transformed to a 0 to 100 scale.
**Scoring Procedure**	**Step 1: Transform Score**<br>Items are reversed scored and linearly transformed to a 0-100 scale as follows: 0=100, 1=75, 2=50, 3=25, 4=0.<br><br>**Step 2: Calculate Scores by Dimensions**<br>• If more than 50% of the items in the scale are missing, the scale scores should not be computed,<br>• Mean score = Sum of the items over the number of items answered.<br><br>**Total Score:** Sum of all the items over the number of items answered on all the Scales.
**Interpretation and Analysis of Missing Data**	If more than 50% of the items in the scale are missing, the Scale Scores should not be computed.<br>If 50% or more items are completed: Impute the mean of the completed items in a scale.

CHILD and PARENT Reports for Young Children (ages 5-7)

The **Child and Parent Reports** of the **PedsQL™** 3.0 Cancer Module for Young Children (ages 5-7), are composed of 26 items comprising 8 dimensions.

DESCRIPTION OF THE CANCER MODULE:

Dimensions	Number of Items	Cluster of Items	Reversed Scoring	Direction of Dimensions
Pain and Hurt	2	1-2	1-2	Higher scores indicate lower problems.
Nausea	5	1-5	1-5	
Procedural Anxiety	3	1-3	1-3	
Treatment Anxiety	3	1-3	1-3	
Worry	3	1-3	1-3	
Cognitive Problems	4	1-4	1-4	
Perceived Physical Appearance	3	1-3	1-3	
Communication	3	1-3	1-3	

SCORING OF DIMENSIONS:

**Item Scaling**	5-point Likert scale from 0 (Never) to 4 (Almost always)<br>3-point scale: 0 (Not at all), 2 (Sometimes) and 4 (A lot) for the Child Report for Young Children (ages 5-7)
**Weighting of Items**	No
**Extension of the Scoring Scale**	Scores are transformed on a scale from 0 to 100.
**Scoring Procedure**	**Step 1: Transform Score**<br>Items are reversed scored and linearly transformed to a 0-100 scale as follows: 0=100, 1=75, 2=50, 3=25, 4=0.<br><br>**Step 2: Calculate Scores by Dimensions**<br>• If more than 50% of the items in the scale are missing, the scale scores should not be computed,<br>• Mean score = Sum of the items over the number of items answered.<br><br>**Total Score:** Sum of all the items over the number of items answered on all the Scales.
**Interpretation and Analysis of Missing Data**	If more than 50% of the items in the scale are missing, the Scale Scores should not be computed.<br>If 50% or more items are completed: Impute the mean of the completed items in a scale.

CHILD and PARENT Reports for Children (ages 8-12), Teens (ages 13-18), Young Adults (ages 18-25), and ADULT Report (ages over 26)

The **Child and Parent Reports** of the **PedsQL** TM 3.0 Cancer Module for:
- Children (ages 8-12),
- Teens (ages 13-18),
- Young Adults (ages 18-25),

and the **Adult Report** (ages over 26)
are composed of 27 items comprising 8 dimensions.

DESCRIPTION OF THE CANCER MODULE:

Dimensions	Number of Items	Cluster of Items	Reversed Scoring	Direction of Dimensions
Pain and Hurt	2	1-2	1-2	Higher scores indicate lower problems.
Nausea	5	1-5	1-5	
Procedural Anxiety	3	1-3	1-3	
Treatment Anxiety	3	1-3	1-3	
Worry	3	1-3	1-3	
Cognitive Problems	5	1-5	1-5	
Perceived Physical Appearance	3	1-3	1-3	
Communication	3	1-3	1-3	

SCORING OF DIMENSIONS:

**Item Scaling**	5-point Likert scale from 0 (Never) to 4 (Almost always)<br>3-point scale: 0 (Not at all), 2 (Sometimes) and 4 (A lot) for the Child Report for Young Children (ages 5-7)
**Weighting of Items**	No
**Extension of the Scoring Scale**	Scores are transformed on a scale from 0 to 100.
**Scoring Procedure**	**Step 1: Transform Score**<br>Items are reversed scored and linearly transformed to a 0-100 scale as follows: 0=100, 1=75, 2=50, 3=25, 4=0.<br>**Step 2: Calculate Scores by Dimensions**<br>• If more than 50% of the items in the scale are missing, the scale scores should not be computed,<br>• Mean score = Sum of the items over the number of items answered.<br>**Total Score**: Sum of all the items over the number of items answered on all the Scales.
**Interpretation and Analysis of Missing Data**	If more than 50% of the items in the scale are missing, the Scale Scores should not be computed.<br>If 50% or more items are completed: Impute the mean of the completed items in a scale.

PedsQL ™ 3.0 Cardiac Module

PARENT Report for Toddlers (ages 2-4)

The Parent Report for Toddlers (age 2-4) of the **PedsQL**TM 3.0 Cardiac Module is composed of 23 items comprising 6 dimensions.

DESCRIPTION OF THE CARDIAC MODULE:

Dimensions	Number of Items	Cluster of Items	Reversed Scoring	Direction of Dimensions
Heart problems and Treatment	7	1-7	1-7	Higher scores indicate lower problems.
Treatment II	3	1-3	1-3	
Perceived Physical Appearance	3	1-3	1-3	
Treatment Anxiety	4	1-4	1-4	
Cognitive Problems	3	1-3	1-3	
Communication	3	1-3	1-3	

SCORING OF DIMENSIONS:

**Item Scaling**	5-point Likert scale from 0 (Never) to 4 (Almost always)
**Weighting of Items**	No
**Extension of the Scoring Scale**	Scores are transformed to a 0 to 100 scale.
**Scoring Procedure**	**Step 1: Transform Score**<br>Items are reversed scored and linearly transformed to a 0-100 scale as follows: 0=100, 1=75, 2=50, 3=25, 4=0.<br><br>**Step 2: Calculate scores by Dimensions**<br>• If more than 50% of the items in the scale are missing, the scale scores should not be computed,<br>• Mean score = Sum of the items over the number of items answered.<br><br>**There is no Total Score.**
**Interpretation and Analysis of Missing Data**	If more than 50% of the items in the scale are missing, the Scale Scores should not be computed.<br>If 50% or more items are completed: Impute the mean of the completed items in a scale.

CHILD and PARENT Reports for Young Children (ages 5-7)

The **Child and Parent Reports** of the **PedsQL™** 3.0 Cardiac Module for Young Children (ages 5-7) are composed of 25 items comprising 6 dimensions.

DESCRIPTION OF THE CARDIAC MODULE:

Dimensions	Number of Items	Cluster of Items	Reversed Scoring	Direction of Dimensions
Heart Problems and Treatment	7	1-7	1-7	Higher scores indicate lower problems.
Treatment II	3	1-3	1-3	
Perceived Physical Appearance	3	1-3	1-3	
Treatment Anxiety	4	1-4	1-4	
Cognitive Problems	5	1-5	1-5	
Communication	3	1-3	1-3	

SCORING OF DIMENSIONS:

**Item Scaling**	5-point Likert scale from 0 (Never) to 4 (Almost always)<br>3-point scale: 0 (Not at all), 2 (Sometimes) and 4 (A lot) for the Child Report for Young Children (ages 5-7)
**Weighting of Items**	No
**Extension of the Scoring Scale**	Scores are transformed on a scale from 0 to 100.
**Scoring Procedure**	**Step 1: Transform Score**<br>Items are reversed scored and linearly transformed to a 0-100 scale as follows: 0=100, 1=75, 2=50, 3=25, 4=0<br>**Step 2: Calculate Scores by Dimensions**<br>• If more than 50% of the items in the scale are missing, the scale scores should not be computed,<br>• Mean score = Sum of the items over the number of items answered.<br>**There is no Total Score**
**Interpretation and Analysis of Missing Data**	If more than 50% of the items in the scale are missing, the Scale Scores should not be computed.<br>If 50% or more items are completed: Impute the mean of the completed items in a scale.

CHILD and PARENT Reports for Children (ages 8-12), Teens (ages 13-18), Young Adults (ages 18-25), and Adults (ages over 26)

The **Child and Parent Reports** of the **PedsQL™** 3.0 Cardiac Module for:

- Children (ages 8-12),
- Teens (ages 13-18),
- Young Adults (ages 18-25),
- **Adults** (ages over 26)

are composed of 27 items comprising 6 dimensions.

DESCRIPTION OF THE CARDIAC MODULE:

Dimensions	Number of Items	Cluster of Items	Reversed Scoring	Direction of Dimensions
Heart Problems and Treatment	7	1-7	1-7	Higher scores indicate lower problems.
Treatment II	5	1-5	1-5	
Perceived Physical Appearance	3	1-3	1-3	
Treatment Anxiety	4	1-4	1-4	
Cognitive Problems	5	1-5	1-5	
Communication	3	1-3	1-3	

SCORING OF DIMENSIONS:

**Item Scaling**	5-point Likert scale from 0 (Never) to 4 (Almost always)
**Weighting of Items**	No
**Extension of the Scoring Scale**	Scores are transformed on a scale from 0 to 100.
**Scoring Procedure**	**Step 1: Transform Score**<br>Items are reversed scored and linearly transformed to a 0-100 scale as follows: 0=100, 1=75, 2=50, 3=25, 4=0<br>**Step 2: Calculate Scores by Dimensions**<br>• If more than 50% of the items in the scale are missing, the scale scores should not be computed,<br>• Mean score = Sum of the items over the number of items answered.<br>**There is no Total Score**
**Interpretation and Analysis of Missing Data**	If more than 50% of the items in the scale are missing, the Scale Scores should not be computed.<br>If 50% or more items are completed: Impute the mean of the completed items in a scale.

PedsQL ™ 3.0 Cerebral Palsy Module

PARENT Report for Toddlers (ages 2-4)

The Parent Report for Toddlers (ages 2-4) of the **PedsQL™** 3.0 Cerebral Palsy Module is composed of 22 items comprising 5 dimensions.

DESCRIPTION OF THE CEREBRAL PALSY MODULE:

Dimensions	Number of Items	Cluster of Items	Reversed Scoring	Direction of Dimensions
Daily Activities	5	1-5	1-5	Higher scores indicate lower problems.
Movement and Balance	5	1-5	1-5	
Pain and Hurt	4	1-4	1-4	
Fatigue	4	1-4	1-4	
Eating Activities	4	1-4	1-4	

SCORING OF DIMENSIONS:

**Item Scaling**	5-point Likert scale from 0 (Never) to 4 (Almost always)
**Weighting of Items**	No
**Extension of the Scoring Scale**	Scores are transformed to a 0 to 100 scale.
**Scoring Procedure**	**Step 1: Transform Score**<br>Items are reversed scored and linearly transformed to a 0-100 scale as follows: 0=100, 1=75, 2=50, 3=25, 4=0<br><br>**Step 2: Calculate Scores by Dimensions**<br>• If more than 50% of the items in the scale are missing, the scale scores should not be computed,<br>• Mean score = Sum of the items over the number of items answered.<br><br>**There is no Total score**
**Interpretation and Analysis of Missing Data**	If more than 50% of the items in the scale are missing, the Scale Scores should not be computed.<br>If 50% or more items are completed: Impute the mean of the completed items in a scale.

CHILD and PARENT Reports for Young Children (ages 5-7), Children (ages 8-12), Teens (ages 13-18)

The **Child and Parent Reports** of the **PedsQL™** 3.0 Cerebral Palsy Module for:
- Young Children (ages 5-7),
- Children (ages 8-12),
- And Teens (ages 13-18)

are composed of 35 items comprising 7 dimensions.

DESCRIPTION OF THE CEREBRAL PALSY MODULE:

Dimensions	Number of Items	Cluster of Items	Reversed Scoring	Direction of Dimensions
Daily Activities	9	1-9	1-9	Higher scores indicate lower problems.
School Activities	4	1-4	1-4	
Movement and Balance	5	1-5	1-5	
Pain and Hurt	4	1-4	1-4	
Fatigue	4	1-4	1-4	
Eating Activities	5	1-5	1-5	
Speech and Communication	4	1-4	1-4	

SCORING OF DIMENSIONS:

**Item Scaling**	5-point Likert scale from 0 (Never) to 4 (Almost always)<br>3-point scale: 0 (Not at all), 2 (Sometimes) and 4 (A lot) for the Child Report for Young Children (ages 5-7)
**Weighting of Items**	No
**Extension of the Scoring Scale**	Scores are transformed on a scale from 0 to 100.
**Scoring Procedure**	**Step 1: Transform Score**<br>Items are reversed scored and linearly transformed to a 0-100 scale as follows: 0=100, 1=75, 2=50, 3=25, 4=0.<br><br>**Step 2: Calculate Scores by Dimensions**<br>• If more than 50% of the items in the scale are missing, the scale scores should not be computed,<br>• Mean score = Sum of the items over the number of items answered.<br><br>**There is no Total score**
**Interpretation and Analysis of Missing Data**	If more than 50% of the items in the scale are missing, the Scale Scores should not be computed.<br>If 50% or more items are completed: Impute the mean of the completed items in a scale.

PedsQL TM Cognitive Functioning Scale

The **PedsQL**TM Cognitive Functioning Scale is composed of 6 items comprising 1 dimension.

DESCRIPTION OF THE COGNITIVE FUNTIONING SCALE:

Dimensions	Number of Items	Cluster of Items	Reversed Scoring	Direction of Dimensions
Cognitive Functioning	6	1-6	1-6	Higher scores indicate lower problems

SCORING OF DIMENSIONS:

**Item Scaling**	5-point Likert scale from 0 (Never) to 4 (Almost always)<br>3-point scale: 0 (Not at all), 2 (Sometimes) and 4 (A lot) for the Child Report for Young Children (ages 5-7)
**Weighting of Items**	No
**Extension of the Scoring Scale**	Scores are transformed on a scale from 0 to 100.
**Scoring Procedure**	**Step 1: Transform Score**<br>Items are reversed scored and linearly transformed to a 0-100 scale as follows: 0=100, 1=75, 2=50, 3=25, 4=0.<br>**Step 2: Calculate Total Score**<br>Sum of all the items over the number of items answered
**Interpretation and Analysis of Missing Data**	If more than 50% of the items in the scale are missing, the Scale Score should not be computed.<br>If 50% or more items are completed: Impute the mean of the completed items in a scale.

PedsQL ™ 3.0 Diabetes Module

The **PedsQL™** 3.0 Diabetes Module is composed of 28 items comprising 5 dimensions.

DESCRIPTION OF THE DIABETES MODULE:

Dimensions	Number of Items	Cluster of Items	Reversed Scoring	Direction of Dimensions
Diabetes	11	1-11	1-11	Higher scores indicate lower problems.
Treatment I	4	1-4	1-4	
Treatment II	7	1-7	1-7	
Worry	3	1-3	1-3	
Communication	3	1-3	1-3	

SCORING OF DIMENSIONS:

**Item Scaling**	5-point scale from 0 (Never) to 4 (Almost always)<br>3-point scale: 0 (Not at all), 2 (Sometimes) and 4 (A lot) for the Child Report for Young Children (ages 5-7)
**Weighting of Items**	No
**Extension of the Scoring Scale**	Scores are transformed on a scale from 0 to 100.
**Scoring Procedure**	**Step 1: Transform Score**<br>Items are reversed scored and linearly transformed to a 0-100 scale as follows: 0=100, 1=75, 2=50, 3=25, 4=0.<br>**Step 2: Calculate scores by Dimension**<br>• If more than 50% of the items in the scale are missing, the scale scores should not be computed,<br>• Mean score = Sum of the items over the number of items answered.<br>**Total Score**: Sum of all the items over the number of items answered on all the Scales.
**Interpretation and Analysis of Missing Data**	If more than 50% of the items in the scale are missing, the Scale Scores should not be computed.<br>If 50% or more items are completed: Impute the mean of the completed items in a scale.

PedsQL TM 3.2 Diabetes Module

The **PedsQL™** 3.2 Diabetes Module is composed of 33 items comprising 5 dimensions for ages 8-45 years. For ages 2-7 years, the **PedsQL™** 3.2 Diabetes Module is composed of 32 items comprising 5 dimensions (one less item for the Worry Scale).

DESCRIPTION OF THE DIABETES MODULE:

Dimensions	Number of Items	Cluster of Items	Reversed Scoring	Direction of Dimensions
Diabetes Symptoms	15	1-15	1-15	Higher scores indicate lower problems.
Treatment I	5	1-5	1-5	
Treatment II	6	1-6	1-6	
Worry	3 (2 ages 2-7)	1-3 (or 1-2)	1-3 (or 1-2)	
Communication	4	1-4	1-4	

SCORING OF DIMENSIONS:

**Item Scaling**	5-point scale from 0 (Never) to 4 (Almost always)<br>3-point scale: 0 (Not at all), 2 (Sometimes) and 4 (A lot) for the Child Report for Young Children (ages 5-7)
**Weighting of Items**	No
**Extension of the Scoring Scale**	Scores are transformed on a scale from 0 to 100.
**Scoring Procedure**	**Step 1: Transform Score**<br><br>Items are reversed scored and linearly transformed to a 0-100 scale as follows: 0=100, 1=75, 2=50, 3=25, 4=0.<br><br>**Step 2: Calculate Scores**<br>Score by Dimensions:<br>• If more than 50% of the items in the scale are missing, the scale scores should not be computed,<br>• Mean score = Sum of the items over the number of items answered.<br><br>Diabetes Symptoms Summary Score = Diabetes Symptoms Scale Score<br><br>Diabetes Management Summary Score = Sum of the items over the number of items answered in the Treatment I, Treatment II, Worry, and Communication Scales.<br><br>**Total Score**: Sum of all the items over the number of items answered on all the Scales.
**Interpretation and Analysis of Missing Data**	If more than 50% of the items in the scale are missing, the Scale Scores should not be computed.<br>If 50% or more items are completed: Impute the mean of the completed items in a scale.

PedsQL TM 3.0 Duchenne Muscular Dystrophy Module

PARENT Report for Young Children (ages 5-7)

The **Parent Report** of the **PedsQL™ 3.0 Duchenne Muscular Dystrophy Module** for Young Children (ages 5-7) is composed of 18 items comprising 4 dimensions.

DESCRIPTION OF THE DUCHENNE MUSCULAR DYSTROPHY MODULE:

Dimensions	Number of Items	Cluster of Items	Reversed Scoring	Direction of Dimensions
Daily Activities	5	1-5	1-5	Higher scores indicate lower problems.
Treatment	4	1-4	1-4	
Worry	6	1-6	1-6	
Communication	3	1-3	1-3	

SCORING OF DIMENSIONS:

**Item Scaling**	5-point Likert scale from 0 (Never) to 4 (Almost always)
**Weighting of Items**	No
**Extension of the Scoring Scale**	Scores are transformed on a scale from 0 to 100.
**Scoring Procedure**	**Step 1: Transform Score**<br>Items are reversed scored and linearly transformed to a 0-100 scale as follows: 0=100, 1=75, 2=50, 3=25, 4=0<br>**Step 2: Calculate Scores by Dimensions**<br>• If more than 50% of the items in the scale are missing, the scale scores should not be computed,<br>• Mean score = Sum of the items over the number of items answered.<br>**No Total Score**
**Interpretation and Analysis of Missing Data**	If more than 50% of the items in the scale are missing, the Scale Scores should not be computed.<br>If 50% or more items are completed: Impute the mean of the completed items in a scale.

CHILD and PARENT Reports for Children (ages 8-12), Teens (ages 13-18) and Young Adults (ages 18-25)

The **Child and Parent Reports** of the **PedsQL[TM] 3.0 Duchenne Muscular Dystrophy Module** for:

- Children (ages 8-12),
- Teens (ages 13-18)
- Young Adults (ages 18-25)

are composed of 18 items comprising 4 dimensions.

DESCRIPTION OF THE DUCHENNE MUSCULAR DYSTROPHY MODULE:

Dimensions	Number of Items	Cluster of Items	Reversed Scoring	Direction of Dimensions
Daily Activities	5	1-5	1-5	Higher scores indicate lower problems.
Treatment	4	1-4	1-4	
Worry	6	1-6	1-6	
Communication	3	1-3	1-3	

SCORING OF DIMENSIONS:

**Item Scaling**	5-point Likert scale from 0 (Never) to 4 (Almost always)
**Weighting of Items**	No
**Extension of the Scoring Scale**	Scores are transformed on a scale from 0 to 100.
**Scoring Procedure**	**Step 1: Transform Score**<br>Items are reversed scored and linearly transformed to a 0-100 scale as follows: 0=100, 1=75, 2=50, 3=25, 4=0<br><br>**Step 2: Calculate Scores by Dimensions**<br>• If more than 50% of the items in the scale are missing, the scale scores should not be computed,<br>• Mean score = Sum of the items over the number of items answered.<br><br>**No Total Score**
**Interpretation and Analysis of Missing Data**	If more than 50% of the items in the scale are missing, the Scale Scores should not be computed.<br>If 50% or more items are completed: Impute the mean of the completed items in a scale.

PedsQL TM 3.0 End Stage Renal Disease Module

PARENT Report for Toddlers (ages 2-4)

The Parent Report for Toddlers (ages 2-4) of the **PedsQL**™ 3.0 End Stage Renal Disease Module is composed of 13 items comprising 4 dimensions.

DESCRIPTION OF THE END STAGE RENAL DISEASE MODULE:

Dimensions	Number of Items	Cluster of Items	Reversed Scoring	Direction of Dimensions
General Fatigue	3	1-3	1-3	Higher scores indicate lower problems.
About My Kidney Disease	4	1-4	1-4	
Treatment Problems	3	1-3	1-3	
Worry	3	1-3	1-3	

SCORING OF DIMENSIONS:

**Item Scaling**	5-point Likert scale from 0 (Never) to 4 (Almost always)
**Weighting of Items**	No
**Extension of the Scoring Scale**	Scores are transformed to a 0 to 100 scale.
**Scoring Procedure**	**Step 1: Transform Score**<br>Items are reversed scored and linearly transformed to a 0-100 scale as follows: 0=100, 1=75, 2=50, 3=25, 4=0.<br><br>**Step 2: Calculate Scores by Dimensions**<br>• If more than 50% of the items in the scale are missing, the scale scores should not be computed,<br>• Mean score = Sum of the items over the number of items answered.<br><br>**Total Score**: Sum of all the items over the number of items answered on all the Scales.
**Interpretation and Analysis of Missing Data**	If more than 50% of the items in the scale are missing, the Scale Scores should not be computed.<br>If 50% or more items are completed: Impute the mean of the completed items in a scale.

CHILD and PARENT Reports for Young Children (ages 5-7), Children (ages 8-12), Teens (ages 13-18), and Young Adults (ages 18-25)

The **Child and Parent Reports** of the **PedsQL™** 3.0 End Stage Renal Disease Module for:
- Young Children (ages 5-7),
- Children (ages 8-12),
- And Teens ages (13-18),
- Young Adults (ages 18-25)

are composed of 34 items comprising 7 dimensions.

DESCRIPTION OF THE END STAGE RENAL DISEASE MODULE:

Dimensions	Number of Items	Cluster of Items	Reversed Scoring	Direction of Dimensions
General Fatigue	4	1-4	1-4	Higher scores indicate lower problems.
About My Kidney Disease	5	1-5	1-5	
Treatment Problems	4	1-4	1-4	
Family and Peer Interaction	3	1-3	1-3	
Worry	10	1-10	1-10	
Perceived Physical Appearance	3	1-3	1-3	
Communication	5	1-5	1-5	

SCORING OF DIMENSIONS:

**Item Scaling**	5-point Likert scale from 0 (Never) to 4 (Almost always)<br>3-point scale: 0 (Not at all), 2 (Sometimes) and 4 (A lot) for the Young Children (ages 5-7) child report
**Weighting of Items**	No
**Extension of the Scoring Scale**	Scores are transformed on a scale from 0 to 100.
**Scoring Procedure**	**<u>Step 1: Transform Score</u>**<br><br>Items are reversed scored and linearly transformed to a 0-100 scale as follows: 0=100, 1=75, 2=50, 3=25, 4=0.<br><br>**<u>Step 2: Calculate Scores</u>**<br><u>Score by Dimensions:</u><br>• If more than 50% of the items in the scale are missing, the scale scores should not be computed,<br>• Mean score = Sum of the items over the number of items answered.<br><br>**<u>Total Score</u>**: Sum of all the items over the number of items answered on all the Scales.
**Interpretation and Analysis of Missing Data**	If more than 50% of the items in the scale are missing, the Scale Scores should not be computed.<br>If 50% or more items are completed: Impute the mean of the completed items in a scale.

PedsQL TM 3.0 Eosinophilic Esophagitis Module

PARENT Report for Toddlers (ages 2-4)

The Parent Report for Toddlers (age 2-4) of the **PedsQL™** 3.0 Eosinophilic Esophagitis Module is composed of 21 items comprising 6 dimensions.

DESCRIPTION OF THE EOSINOPHILIC ESOPHAGITIS MODULE:

Dimensions	Number of Items	Cluster of Items	Reversed Scoring	Direction of Dimensions
Symptoms I	3	1-3	1-3	Higher scores indicate lower problems.
Symptoms II	4	1-4	1-4	
Treatment	4	1-4	1-4	
Worry	4	1-4	1-4	
Food and Eating	3	1-3	1-3	
Food Feelings	3	1-3	1-3	

Note: Feeding Tube Scale not scored.

SCORING OF DIMENSIONS:

**Item Scaling**	5-point Likert scale from 0 (Never) to 4 (Almost always)
**Weighting of Items**	No
**Extension of the Scoring Scale**	Scores are transformed to a 0 to 100 scale.
**Scoring Procedure**	**Step 1: Transform Score**<br>Items are reversed scored and linearly transformed to a 0-100 scale as follows: 0=100, 1=75, 2=50, 3=25, 4=0.<br><br>**Step 2: Calculate scores by Dimensions**<br>• If more than 50% of the items in the scale are missing, the scale scores should not be computed,<br>• Mean score = Sum of the items over the number of items answered.<br><br>Symptoms Total Scale Score = Sum of the items over the number of items answered in the Symptoms I and Symptoms II Scales.<br><br>**Total Score:** Sum of all the items over the number of items answered on all the Scales except Feeding Tube Scale.
**Interpretation and Analysis of Missing Data**	If more than 50% of the items in the scale are missing, the Scale Scores should not be computed.<br>If 50% or more items are completed: Impute the mean of the completed items in a scale.

REFERENCES:

Franciosi, J.P., Hommel, K.A., Bendo, C.B., King, E.C., Collins, M.H., Eby, M.D., Marsolo, K., Abonia, J.P., von Tiehl, K.F., Putnam, P.E., Greenler, A.J., Greenberg, A.B., Bryson,

CHILD and PARENT Reports for Young Children (ages 5-7)

The **Child and Parent Reports** of the **PedsQL**™ 3.0 Eosinophilic Esophagitis Module for Young Children (ages 5-7) are composed of 26 items comprising 7 dimensions.

DESCRIPTION OF THE EOSINOPHILIC ESOPHAGITIS MODULE:

Dimensions	Number of Items	Cluster of Items	Reversed Scoring	Direction of Dimensions
Symptoms I	3	1-3	1-3	Higher scores indicate lower problems.
Symptoms II*	4	1-4	1-4	
Treatment	4	1-4	1-4	
Worry	4	1-4	1-4	
Communication	4	1-4	1-4	
Food and Eating	4	1-4	1-4	
Food Feelings	3	1-3	1-3	

*Symptoms II Scale not scored for child self-report ages 5-7 years.
Note: Feeding Tube Scale not scored.

SCORING OF DIMENSIONS:

**Item Scaling**	5-point Likert scale from 0 (Never) to 4 (Almost always)<br>3-point scale: 0 (Not at all), 2 (Sometimes) and 4 (A lot) for the Child Report for Young Children (ages 5-7)
**Weighting of Items**	No
**Extension of the Scoring Scale**	Scores are transformed on a scale from 0 to 100.
**Scoring Procedure**	**Step 1: Transform Score**<br>Items are reversed scored and linearly transformed to a 0-100 scale as follows: 0=100, 1=75, 2=50, 3=25, 4=0<br><br>**Step 2: Calculate Scores by Dimensions**<br>• If more than 50% of the items in the scale are missing, the scale scores should not be computed,<br>• Mean score = Sum of the items over the number of items answered.<br><br>Symptoms Total Scale Score = Sum of the items over the number of items answered in the Symptoms I and Symptoms II Scales. No Symptoms Total Scale Score for child self-report ages 5-7 years.<br><br>**Total Score:** Sum of all the items over the number of items answered on all the Scales for parent report except Feeding Tube Scale. For child self-report ages 5-7, sum all of the items over the number of items answered on all Scales except for Symptoms II and Feeding Tube Scale.

**Interpretation and Analysis of Missing Data**	If more than 50% of the items in the scale are missing, the Scale Scores should not be computed.<br>If 50% or more items are completed: Impute the mean of the completed items in a scale.

CHILD and PARENT Reports for Children (ages 8-12) and Teens (ages 13-18)

The **Child and Parent Reports** of the **PedsQL**TM 3.0 Eosinophilic Esophagitis Module for:

- Children (ages 8-12),
- And Teens (ages 13-18)

are composed of 33 items comprising 7 dimensions.

DESCRIPTION OF THE EOSINOPHILIC ESOPHAGITIS MODULE:

Dimensions	Number of Items	Cluster of Items	Reversed Scoring	Direction of Dimensions
Symptoms I	6	1-6	1-6	Higher scores indicate lower problems.
Symptoms II	4	1-4	1-4	
Treatment	5	1-5	1-5	
Worry	6	1-6	1-6	
Communication	5	1-5	1-5	
Food and Eating	4	1-4	1-4	
Food Feelings	3	1-3	1-3	

Note: Feeding Tube Scale not scored.

SCORING OF DIMENSIONS:

**Item Scaling**	5-point Likert scale from 0 (Never) to 4 (Almost always)
**Weighting of Items**	No
**Extension of the Scoring Scale**	Scores are transformed on a scale from 0 to 100.
**Scoring Procedure**	**Step 1: Transform Score**<br>Items are reversed scored and linearly transformed to a 0-100 scale as follows: 0=100, 1=75, 2=50, 3=25, 4=0<br><br>**Step 2: Calculate Scores by Dimensions**<br>• If more than 50% of the items in the scale are missing, the scale scores should not be computed,<br>• Mean score = Sum of the items over the number of items answered.<br><br>Symptoms Total Scale Score = Sum of the items over the number of items answered in the Symptoms I and Symptoms II Scales.<br><br>**Total Score:** Sum of all the items over the number of items answered on all the Scales except Feeding Tube Scale.
**Interpretation and Analysis of Missing Data**	If more than 50% of the items in the scale are missing, the Scale Scores should not be computed.<br>If 50% or more items are completed: Impute the mean of the completed items in a scale.

PedsQL TM Eosinophilic Esophagitis Symptoms Scales

PARENT Report for Toddlers (ages 2-4)

The Parent Report for Toddlers (age 2-4) of the **PedsQL™** Eosinophilic Esophagitis Symptoms Scales is composed of 7 items comprising 2 dimensions.

DESCRIPTION OF THE EOSINOPHILIC ESOPHAGITIS MODULE:

Dimensions	Number of Items	Cluster of Items	Reversed Scoring	Direction of Dimensions
Symptoms I	3	1-3	1-3	Higher scores indicate lower problems.
Symptoms II	4	1-4	1-4	

SCORING OF DIMENSIONS:

**Item Scaling**	5-point Likert scale from 0 (Never) to 4 (Almost always)
**Weighting of Items**	No
**Extension of the Scoring Scale**	Scores are transformed to a 0 to 100 scale.
**Scoring Procedure**	**Step 1: Transform Score**<br>Items are reversed scored and linearly transformed to a 0-100 scale as follows: 0=100, 1=75, 2=50, 3=25, 4=0.<br><br>**Step 2: Calculate scores by Dimensions**<br>• If more than 50% of the items in the scale are missing, the scale scores should not be computed,<br>• Mean score = Sum of the items over the number of items answered.<br><br>**Symptoms Total Scale Score** = Sum of the items over the number of items answered in the Symptoms I and Symptoms II Scales.
**Interpretation and Analysis of Missing Data**	If more than 50% of the items in the scale are missing, the Scale Scores should not be computed.<br>If 50% or more items are completed: Impute the mean of the completed items in a scale.

PARENT Reports for Young Children (ages 5-7)

The **Parent Reports** of the **PedsQL™** Eosinophilic Esophagitis Symptoms Scales for Young Children (ages 5-7) are composed of 7 items comprising 2 dimensions. **Please note that there is no child self-report for ages 5-7.**

DESCRIPTION OF THE EOSINOPHILIC ESOPHAGITIS MODULE:

Dimensions	Number of Items	Cluster of Items	Reversed Scoring	Direction of Dimensions
Symptoms I	3	1-3	1-3	Higher scores indicate lower problems.
Symptoms II	4	1-4	1-4	

SCORING OF DIMENSIONS:

**Item Scaling**	5-point Likert scale from 0 (Never) to 4 (Almost always)<br>3-point scale: 0 (Not at all), 2 (Sometimes) and 4 (A lot) for the Child Report for Young Children (ages 5-7)
**Weighting of Items**	No
**Extension of the Scoring Scale**	Scores are transformed on a scale from 0 to 100.
**Scoring Procedure**	**Step 1: Transform Score**<br>Items are reversed scored and linearly transformed to a 0-100 scale as follows: 0=100, 1=75, 2=50, 3=25, 4=0<br><br>**Step 2: Calculate Scores by Dimensions**<br>• If more than 50% of the items in the scale are missing, the scale scores should not be computed,<br>• Mean score = Sum of the items over the number of items answered.<br><br>**Symptoms Total Scale Score** = Sum of the items over the number of items answered in the Symptoms I and Symptoms II Scales.
**Interpretation and Analysis of Missing Data**	If more than 50% of the items in the scale are missing, the Scale Scores should not be computed.<br>If 50% or more items are completed: Impute the mean of the completed items in a scale.

CHILD and PARENT Reports for Children (ages 8-12) and Teens (ages 13-18)

The **Child and Parent Reports** of the **PedsQL™** Eosinophilic Esophagitis Symptoms Scales for:

- Children (ages 8-12),
- And Teens (ages 13-18)

are composed of 10 items comprising 2 dimensions.

DESCRIPTION OF THE EOSINOPHILIC ESOPHAGITIS MODULE:

Dimensions	Number of Items	Cluster of Items	Reversed Scoring	Direction of Dimensions
Symptoms I	6	1-6	1-6	Higher scores indicate lower problems.
Symptoms II	4	1-4	1-4	

SCORING OF DIMENSIONS:

**Item Scaling**	5-point Likert scale from 0 (Never) to 4 (Almost always)
**Weighting of Items**	No
**Extension of the Scoring Scale**	Scores are transformed on a scale from 0 to 100.
**Scoring Procedure**	**Step 1: Transform Score**<br>Items are reversed scored and linearly transformed to a 0-100 scale as follows: 0=100, 1=75, 2=50, 3=25, 4=0<br><br>**Step 2: Calculate Scores by Dimensions**<br>• If more than 50% of the items in the scale are missing, the scale scores should not be computed,<br>• Mean score = Sum of the items over the number of items answered.<br><br>**Symptoms Total Scale Score** = Sum of the items over the number of items answered in the Symptoms I and Symptoms II Scales.
**Interpretation and Analysis of Missing Data**	If more than 50% of the items in the scale are missing, the Scale Scores should not be computed.<br>If 50% or more items are completed: Impute the mean of the completed items in a scale.

PedsQL ™ 3.0 Epilepsy Module

PARENT Report for Toddlers (ages 2-4)

The Parent Report for Toddlers (ages 2-4) of the **PedsQL™** 3.0 Epilepsy Module is composed of 22 items comprising 5 dimensions.

DESCRIPTION OF THE EPILEPSY MODULE:

Dimensions	Number of Items	Cluster of Items	Reversed Scoring	Direction of Dimensions
Impact	6	1-6	1-6	Higher scores indicate lower problems.
Cognitive Functioning	5	1-5	1-5	
Sleep/Rest	2	1-2	1-2	
Executive Functioning	4	1-4	1-4	
Mood/Behavior	5	1-5	1-5	

SCORING OF DIMENSIONS:

**Item Scaling**	5-point Likert scale from 0 (Never) to 4 (Almost always)
**Weighting of Items**	No
**Extension of the Scoring Scale**	Scores are transformed to a 0 to 100 scale.
**Scoring Procedure**	**<u>Step 1: Transform Score</u>**<br>Items are reversed scored and linearly transformed to a 0-100 scale as follows: 0=100, 1=75, 2=50, 3=25, 4=0<br><br>**<u>Step 2: Calculate Scores by Dimensions</u>**<br>• If more than 50% of the items in the scale are missing, the scale scores should not be computed,<br>• Mean score = Sum of the items over the number of items answered.<br><br>**There is no Total score**
**Interpretation and Analysis of Missing Data**	If more than 50% of the items in the scale are missing, the Scale Scores should not be computed.<br>If 50% or more items are completed: Impute the mean of the completed items in a scale.

CHILD Reports for Young Children (ages 5-7)

The **Child Reports** of the **PedsQL™** 3.0 Epilepsy Module for:

- Young Children (ages 5-7)

are composed of 28 items comprising 5 dimensions.

DESCRIPTION OF THE EPILEPSY MODULE:

Dimensions	Number of Items	Cluster of Items	Reversed Scoring	Direction of Dimensions
Impact	8	1-8	1-8	Higher scores indicate lower problems.
Cognitive Functioning	6	1-6	1-6	
Sleep/Rest	3	1-3	1-3	
Executive Functioning	6	1-6	1-6	
Mood/Behavior	5	1-5	1-5	

SCORING OF DIMENSIONS:

**Item Scaling**	3-point scale: 0 (Not at all), 2 (Sometimes) and 4 (A lot) for the Child Report for Young Children (ages 5-7)
**Weighting of Items**	No
**Extension of the Scoring Scale**	Scores are transformed on a scale from 0 to 100.
**Scoring Procedure**	**Step 1: Transform Score**<br>Items are reversed scored and linearly transformed to a 0-100 scale as follows: 0=100, 2=50, 4=0.<br><br>**Step 2: Calculate Scores by Dimensions**<br>• If more than 50% of the items in the scale are missing, the scale scores should not be computed,<br>• Mean score = Sum of the items over the number of items answered.<br><br>**There is no Total score**
**Interpretation and Analysis of Missing Data**	If more than 50% of the items in the scale are missing, the Scale Scores should not be computed.<br>If 50% or more items are completed: Impute the mean of the completed items in a scale.

PARENT Reports for Young Children (ages 5-7)

The **Parent Reports** of the **PedsQL™** 3.0 Epilepsy Module for:

- Young Children (ages 5-7)

are composed of 29 items comprising 5 dimensions.

DESCRIPTION OF THE EPILEPSY MODULE:

Dimensions	Number of Items	Cluster of Items	Reversed Scoring	Direction of Dimensions
Impact	9	1-9	1-9	Higher scores indicate lower problems.
Cognitive Functioning	6	1-6	1-6	
Sleep/Rest	3	1-3	1-3	
Executive Functioning	6	1-6	1-6	
Mood/Behavior	5	1-5	1-5	

SCORING OF DIMENSIONS:

**Item Scaling**	5-point Likert scale from 0 (Never) to 4 (Almost always)
**Weighting of Items**	No
**Extension of the Scoring Scale**	Scores are transformed on a scale from 0 to 100.
**Scoring Procedure**	**Step 1: Transform Score**<br>Items are reversed scored and linearly transformed to a 0-100 scale as follows: 0=100, 1=75, 2=50, 3=25, 4=0.<br><br>**Step 2: Calculate Scores by Dimensions**<br>• If more than 50% of the items in the scale are missing, the scale scores should not be computed,<br>• Mean score = Sum of the items over the number of items answered.<br><br>**There is no Total score**
**Interpretation and Analysis of Missing Data**	If more than 50% of the items in the scale are missing, the Scale Scores should not be computed.<br>If 50% or more items are completed: Impute the mean of the completed items in a scale.

CHILD and PARENT Reports for Children (ages 8-12), Teens (ages 13-18), Young Adults (ages 18-25)

The **Child and Parent Reports** of the **PedsQL**[TM] 3.0 Epilepsy Module for:
- Children (ages 8-12),
- Teens (ages 13-18)
- Young Adults (18-25)

are composed of 29 items comprising 5 dimensions.

DESCRIPTION OF THE EPILEPSY MODULE:

Dimensions	Number of Items	Cluster of Items	Reversed Scoring	Direction of Dimensions
Impact	9	1-9	1-9	Higher scores indicate lower problems.
Cognitive Functioning	6	1-6	1-6	
Sleep/Rest	3	1-3	1-3	
Executive Functioning	6	1-6	1-6	
Mood/Behavior	5	1-5	1-5	

SCORING OF DIMENSIONS:

**Item Scaling**	5-point Likert scale from 0 (Never) to 4 (Almost always)
**Weighting of Items**	No
**Extension of the Scoring Scale**	Scores are transformed on a scale from 0 to 100.
**Scoring Procedure**	**Step 1: Transform Score**<br>Items are reversed scored and linearly transformed to a 0-100 scale as follows: 0=100, 1=75, 2=50, 3=25, 4=0.<br><br>**Step 2: Calculate Scores by Dimensions**<br>• If more than 50% of the items in the scale are missing, the scale scores should not be computed,<br>• Mean score = Sum of the items over the number of items answered.<br><br>**There is no Total score**
**Interpretation and Analysis of Missing Data**	If more than 50% of the items in the scale are missing, the Scale Scores should not be computed.<br>If 50% or more items are completed: Impute the mean of the completed items in a scale.

PedsQL TM 2.0 Family Impact Module

PARENT Report

The Parent report of the **PedsQL™** 2.0 Family impact Module is composed of 36 items comprising 8 dimensions.

DESCRIPTION OF THE FAMILY IMPACT MODULE:

Dimensions	Number of Items	Cluster of Items	Reversed Scoring	Direction of Dimensions
Physical Functioning	6	1-6	1-6	Higher scores indicate better functioning.
Emotional Functioning	5	1-5	1-5	
Social Functioning	4	1-4	1-4	
Cognitive Functioning	5	1-5	1-5	
Communication	3	1-3	1-3	
Worry	5	1-5	1-5	
Daily Activities	3	1-3	1-3	
Family Relationships	5	1-5	1-5	

SCORING OF DIMENSIONS:

**Item Scaling**	5-point Likert scale from 0 (Never) to 4 (Almost always)
**Weighting of Items**	No
**Extension of the Scoring Scale**	Scores are transformed to a 0 to 100 scale.
**Scoring Procedure**	**Step 1: Transform Score**<br>Items are reversed scored and linearly transformed to a 0-100 scale as follows: 0=100, 1=75, 2=50, 3=25, 4=0<br>**Step 2: Calculate Scores by Dimensions**<br>• If more than 50% of the items in the scale are missing, the scale scores should not be computed,<br>• Mean score = Sum of the items over the number of items answered.<br>**Step 3: Total Scores**<br>• The Total Score is the sum of all 36 items divided by the number of items answered<br>• The Parent HRQL Summary Score (20 items) is computed as the sum of the items divided by the number of items answered in the Physical, Emotional, Social, and Cognitive Functioning scales.<br>• The Family Functioning Summary Score (8 items) is computed as the sum of the items divided by the number of items answered in the Daily Activities and family Relationships scales.

**Interpretation and Analysis of Missing Data**	If more than 50% of the items in the scale are missing, the Scale Scores should not be computed.<br>If 50% or more items are completed: Impute the mean of the completed items in a scale.

PedsQL TM 3.0 Gastrointestinal Symptoms Module

PARENT Report for Toddlers (ages 2-4)

The Parent Report for Toddlers (age 2-4) of the **PedsQL™** 3.0 Gastrointestinal Symptoms Module is composed of 74 items comprising 14 dimensions.

DESCRIPTION OF THE GASTROINTESTINAL SYMPTOMS MODULE:

Dimensions	Number of Items	Cluster of Items	Reversed Scoring	Direction of Dimensions
Stomach Pain and Hurt	6	1-6	1-6	Higher scores indicate lower problems.
Stomach Discomfort When Eating	5	1-5	1-5	
Food and Drink Limits	6	1-6	1-6	
Trouble Swallowing	3	1-3	1-3	
Heart Burn and Reflux	4	1-4	1-4	
Nausea and Vomiting	4	1-4	1-4	
Gas and Bloating	7	1-7	1-7	
Constipation	14	1-14	1-14	
Blood in Poop	2	1-2	1-2	
Diarrhea	7	1-7	1-7	
Worry About Going Poop	5	1-5	1-5	
Worry About Stomach Aches	2	1-2	1-2	
Medicines	4	1-4	1-4	
Communication	5	1-5	1-5	

SCORING OF DIMENSIONS:

**Item Scaling**	5-point Likert scale from 0 (Never) to 4 (Almost always)
**Weighting of Items**	No
**Extension of the Scoring Scale**	Scores are transformed to a 0 to 100 scale.
**Scoring Procedure**	**Step 1: Transform Score**<br>Items are reversed scored and linearly transformed to a 0-100 scale as follows: 0=100, 1=75, 2=50, 3=25, 4=0.<br><br>**Step 2: Calculate scores by Dimensions**<br>• If more than 50% of the items in the scale are missing, the scale scores should not be computed,<br>• Mean score = Sum of the items over the number of items answered.

	Symptoms Total Scales Score = Sum of the items over the number of items answered in the 10 Symptoms Scales.<br><br>**Total Score:** Sum of all the items over the number of items answered on all the Scales.
**Interpretation and Analysis of Missing Data**	If more than 50% of the items in the scale are missing, the Scale Scores should not be computed.<br>If 50% or more items are completed: Impute the mean of the completed items in a scale.

CHILD and PARENT Report for Young Children (ages 5-7)

The **Child and Parent Reports** of the **PedsQL™** 3.0 Gastrointestinal Symptoms Module for Young Children (ages 5-7) are composed of 74 items comprising 14 dimensions.

DESCRIPTION OF THE GASTROINTESTINAL SYMPTOMS MODULE:

Dimensions	Number of Items	Cluster of Items	Reversed Scoring	Direction of Dimensions
Stomach Pain and Hurt	6	1-6	1-6	Higher scores indicate lower problems.
Stomach Discomfort When Eating	5	1-5	1-5	
Food and Drink Limits	6	1-6	1-6	
Trouble Swallowing	3	1-3	1-3	
Heart Burn and Reflux	4	1-4	1-4	
Nausea and Vomiting	4	1-4	1-4	
Gas and Bloating	7	1-7	1-7	
Constipation	14	1-14	1-14	
Blood in Poop	2	1-2	1-2	
Diarrhea	7	1-7	1-7	
Worry About Going Poop	5	1-5	1-5	
Worry About Stomach Aches	2	1-2	1-2	
Medicines	4	1-4	1-4	
Communication	5	1-5	1-5	

SCORING OF DIMENSIONS:

**Item Scaling**	5-point Likert scale from 0 (Never) to 4 (Almost always)<br>3-point scale: 0 (Not at all), 2 (Sometimes) and 4 (A lot) for the Child Report for Young Children (ages 5-7)
**Weighting of Items**	No
**Extension of the Scoring Scale**	Scores are transformed on a scale from 0 to 100.
**Scoring Procedure**	**Step 1: Transform Score**<br>Items are reversed scored and linearly transformed to a 0-100 scale as follows: 0=100, 1=75, 2=50, 3=25, 4=0<br>**Step 2: Calculate Scores by Dimensions**<br>• If more than 50% of the items in the scale are missing, the scale scores

	should not be computed,<br>• Mean score = Sum of the items over the number of items answered.<br><br>Symptoms Total Scales Score = Sum of the items over the number of items answered in the 10 Symptoms Scales.<br><br>**Total Score:** Sum of all the items over the number of items answered on all the Scales.
**Interpretation and Analysis of Missing Data**	If more than 50% of the items in the scale are missing, the Scale Scores should not be computed.<br>If 50% or more items are completed: Impute the mean of the completed items in a scale.

CHILD and PARENT Reports for Children (ages 8-12), Teens (ages 13-18) and Young Adults (18-25)

The **Child and Parent Reports** of the **PedsQL™** 3.0 Gastrointestinal Symptoms Module for:

- Children (ages 8-12),
- Teens (ages 13-18),
- Young Adults (ages 18-25)

are composed of 74 items comprising 14 dimensions.

DESCRIPTION OF THE GASTROINTESTINAL SYMPTOMS MODULE:

Dimensions	Number of Items	Cluster of Items	Reversed Scoring	Direction of Dimensions
Stomach Pain and Hurt	6	1-6	1-6	Higher scores indicate lower problems.
Stomach Discomfort When Eating	5	1-5	1-5	
Food and Drink Limits	6	1-6	1-6	
Trouble Swallowing	3	1-3	1-3	
Heart Burn and Reflux	4	1-4	1-4	
Nausea and Vomiting	4	1-4	1-4	
Gas and Bloating	7	1-7	1-7	
Constipation	14	1-14	1-14	
Blood in Poop (Bowel Movement)	2	1-2	1-2	
Diarrhea	7	1-7	1-7	
Worry About Going Poop (Bowel Movements)	5	1-5	1-5	
Worry About Stomach Aches	2	1-2	1-2	
Medicines	4	1-4	1-4	
Communication	5	1-5	1-5	

SCORING OF DIMENSIONS:

**Item Scaling**	5-point Likert scale from 0 (Never) to 4 (Almost always)
**Weighting of Items**	No
**Extension of the Scoring Scale**	Scores are transformed to a 0 to 100 scale.
**Scoring Procedure**	**Step 1: Transform Score**<br>Items are reversed scored and linearly transformed to a 0-100 scale as follows: 0=100, 1=75, 2=50, 3=25, 4=0.

	**Step 2: Calculate scores by Dimensions**<br>• If more than 50% of the items in the scale are missing, the scale scores should not be computed,<br>• Mean score = Sum of the items over the number of items answered.<br><br>Symptoms Total Scales Score = Sum of the items over the number of items answered in the 10 Symptoms Scales.<br><br>**Total Score:** Sum of all the items over the number of items answered on all the Scales.
**Interpretation and Analysis of Missing Data**	If more than 50% of the items in the scale are missing, the Scale Scores should not be computed.<br>If 50% or more items are completed: Impute the mean of the completed items in a scale.

PedsQL ™ 3.0 Gastrointestinal Symptoms Scales™

PARENT Report for Toddlers (ages 2-4)

The Parent Report for Toddlers (age 2-4) of the **PedsQL™** 3.0 Gastrointestinal Symptoms Scales™ is composed of 58 items comprising 10 dimensions.

DESCRIPTION OF THE GASTROINTESTINAL SYMPTOMS SCALES™:

Dimensions	Number of Items	Cluster of Items	Reversed Scoring	Direction of Dimensions
Stomach Pain and Hurt	6	1-6	1-6	Higher scores indicate lower problems.
Stomach Discomfort When Eating	5	1-5	1-5	
Food and Drink Limits	6	1-6	1-6	
Trouble Swallowing	3	1-3	1-3	
Heart Burn and Reflux	4	1-4	1-4	
Nausea and Vomiting	4	1-4	1-4	
Gas and Bloating	7	1-7	1-7	
Constipation	14	1-14	1-14	
Blood in Poop	2	1-2	1-2	
Diarrhea	7	1-7	1-7	

SCORING OF DIMENSIONS:

**Item Scaling**	5-point Likert scale from 0 (Never) to 4 (Almost always)
**Weighting of Items**	No
**Extension of the Scoring Scale**	Scores are transformed to a 0 to 100 scale.
**Scoring Procedure**	**Step 1: Transform Score**<br>Items are reversed scored and linearly transformed to a 0-100 scale as follows: 0=100, 1=75, 2=50, 3=25, 4=0.<br><br>**Step 2: Calculate scores by Dimensions**<br>• If more than 50% of the items in the scale are missing, the scale scores should not be computed,<br>• Mean score = Sum of the items over the number of items answered.<br><br>**Symptoms Total Scales Score** = Sum of the items over the number of items answered in the 10 Symptoms Scales.
**Interpretation and Analysis of Missing Data**	If more than 50% of the items in the scale are missing, the Scale Scores should not be computed.<br>If 50% or more items are completed: Impute the mean of the completed items in a scale.

CHILD and PARENT Reports for Young Children (ages 5-7)

The **Child and Parent Reports** of the **PedsQL™** 3.0 Gastrointestinal Symptoms Scales™ for Young Children (ages 5-7) are composed of 58 items comprising 10 dimensions.

DESCRIPTION OF THE GASTROINTESTINAL SYMPTOMS SCALES™:

Dimensions	Number of Items	Cluster of Items	Reversed Scoring	Direction of Dimensions
Stomach Pain and Hurt	6	1-6	1-6	Higher scores indicate lower problems.
Stomach Discomfort When Eating	5	1-5	1-5	
Food and Drink Limits	6	1-6	1-6	
Trouble Swallowing	3	1-3	1-3	
Heart Burn and Reflux	4	1-4	1-4	
Nausea and Vomiting	4	1-4	1-4	
Gas and Bloating	7	1-7	1-7	
Constipation	14	1-14	1-14	
Blood in Poop	2	1-2	1-2	
Diarrhea	7	1-7	1-7	

SCORING OF DIMENSIONS:

**Item Scaling**	5-point Likert scale from 0 (Never) to 4 (Almost always)<br>3-point scale: 0 (Not at all), 2 (Sometimes) and 4 (A lot) for the Child Report for Young Children (ages 5-7)
**Weighting of Items**	No
**Extension of the Scoring Scale**	Scores are transformed on a scale from 0 to 100.
**Scoring Procedure**	**Step 1: Transform Score**<br>Items are reversed scored and linearly transformed to a 0-100 scale as follows: 0=100, 1=75, 2=50, 3=25, 4=0<br><br>**Step 2: Calculate Scores by Dimensions**<br>• If more than 50% of the items in the scale are missing, the scale scores should not be computed,<br>• Mean score = Sum of the items over the number of items answered.<br><br>**Symptoms Total Scales Score** = Sum of the items over the number of items answered in the 10 Symptoms Scales.
**Interpretation and Analysis of Missing Data**	If more than 50% of the items in the scale are missing, the Scale Scores should not be computed.<br>If 50% or more items are completed: Impute the mean of the completed items in a scale.

CHILD and PARENT Reports for Children (ages 8-12), Teens (ages 13-18) and Young Adults (ages 18-25)

The **Child and Parent Reports** of the **PedsQL™** 3.0 Gastrointestinal Symptoms Scales™ for:
- Children (ages 8-12),
- Teens (ages 13-18),
- Young Adults (ages 18-25)

are composed of 58 items comprising 10 dimensions.

DESCRIPTION OF THE GASTROINTESTINAL SYMPTOMS SCALES™:

Dimensions	Number of Items	Cluster of Items	Reversed Scoring	Direction of Dimensions
Stomach Pain and Hurt	6	1-6	1-6	Higher scores indicate lower problems.
Stomach Discomfort When Eating	5	1-5	1-5	
Food and Drink Limits	6	1-6	1-6	
Trouble Swallowing	3	1-3	1-3	
Heart Burn and Reflux	4	1-4	1-4	
Nausea and Vomiting	4	1-4	1-4	
Gas and Bloating	7	1-7	1-7	
Constipation	14	1-14	1-14	
Blood in Poop (Bowel Movement)	2	1-2	1-2	
Diarrhea	7	1-7	1-7	

SCORING OF DIMENSIONS:

**Item Scaling**	5-point Likert scale from 0 (Never) to 4 (Almost always)
**Weighting of Items**	No
**Extension of the Scoring Scale**	Scores are transformed to a 0 to 100 scale.
**Scoring Procedure**	**Step 1: Transform Score**<br>Items are reversed scored and linearly transformed to a 0-100 scale as follows: 0=100, 1=75, 2=50, 3=25, 4=0.<br>**Step 2: Calculate scores by Dimensions**<br>• If more than 50% of the items in the scale are missing, the scale scores should not be computed,<br>• Mean score = Sum of the items over the number of items answered.<br>**Symptoms Total Scales Score** = Sum of the items over the number of items answered in the 10 Symptoms Scales.
**Interpretation and Analysis of Missing Data**	If more than 50% of the items in the scale are missing, the Scale Scores should not be computed.<br>If 50% or more items are completed: Impute the mean of the completed items in a scale.

PedsQL ™ General Well-Being Scale

CHILD, ADOLESCENT and YOUNG ADULT Report (ages 8-25 years of age)

The **PedsQL™** General Well-Being Scale is composed of 6 items for ages 8-25 years of age, and 1 overall General Health item.

DESCRIPTION OF THE QUESTIONNAIRE (ages 8-25 years):

Dimensions	Number of Items	Cluster of Items	Reversed Scoring	Direction of Dimensions
General Well-Being	6	1-6	No	Higher scores indicate better HRQOL.

Dimensions	Number of Items	Cluster of Items	Reversed Scoring	Direction of Dimensions
General Health	1	1	No	Higher scores indicate better HRQOL.

SCORING OF DIMENSIONS:

**Item Scaling**	5-point Likert scale from 0 (Never) to 4 (Almost always)
**Weighting of Items**	No
**Extension of the Scoring Scale**	Scores are transformed on a scale from 0 to 100.
**Scoring Procedure**	**Step 1: Transform Score**<br>Items are linearly transformed to a 0-100 scale as follows: 0=0, 1=25, 2=50, 3=75, 4=100.<br>**Step 2: Calculate Scores**<br>Score by Dimensions:<br>• If more than 50% of the items in the scale are missing, the scale score should not be computed.<br>• Mean score = Sum of the items over the number of items answered.<br>• The General Health item is not included in the scale score, but is reported separately.
**Interpretation and Analysis of Missing Data**	If more than 50% of the items in the scale are missing, the Scale Score should not be computed.<br>If 50% or more items are completed: Impute the mean of the completed items in a scale.

PedsQL™ Healthcare Satisfaction Generic Module

The **PedsQL**TM Healthcare Satisfaction Generic Module is composed of 24 items comprising 6 dimensions.

DESCRIPTION OF THE MODULE:

Dimensions	Number of Items	Cluster of Items	Reversed Scoring	Direction of Dimensions
Information	5	1-5	No	Higher scores indicate higher satisfaction.
Inclusion of Family	4	1-4		
Communication	5	1-5		
Technical Skills	3	1-3		
Emotional Needs	4	1-4		
Overall Satisfaction	3	1-3		

SCORING OF DIMENSIONS:

**Item Scaling**	5-point Likert scale: 0 (Never) to 4 (Always) and Not Applicable
**Weighting of items**	No
**Extension of the Scoring Scale**	Scores are transformed on a scale from 0 to 100.
**Scoring Procedure**	**Step 1: Transform Score**<br>Items are scored and linearly transformed to a 0-100 scale as follows: 0=0, 1=25, 2=50, 3=75, 4=100<br><br>**Step 2: Calculate Scores by Dimensions**<br>• If 50% or more items are completed, the Mean score = Sum of the items divided by the number of items answered in the scale,<br>• If more than 50% of the items in the scale are missing, the scale scores should not be computed.<br><br>**Total Score**: Mean score = Sum of all the items in all scales divided by the number of items answered on all the scales.
**Interpretation and Analysis of Missing Data**	If more than 50% of the items in the scale are missing, the Scale Scores should not be computed.<br>If 50% or more items are completed: Impute the mean of the completed items in the scale.

PedsQL™ Healthcare Satisfaction Hematology / Oncology Module

The **PedsQL™** Healthcare Satisfaction Hematology / Oncology Module is composed of 25 items comprising 6 dimensions.

DESCRIPTION OF THE MODULE:

Dimensions	Number of Items	Cluster of Items	Reversed Scoring	Direction of Dimensions
General Satisfaction	3	1-3	No	Higher scores indicate higher satisfaction.
Information	5	1-5		
Inclusion of Family	4	1-4		
Communication	5	1-5		
Technical Skills	4	1-4		
Emotional Needs	4	1-4		

SCORING OF DIMENSIONS:

**Item Scaling**	5-point Likert scale: 1 (Very dissatisfied) to 5 (Very satisfied)
**Weighting of Items**	No
**Extension of the Scoring Scale**	Scores are transformed on a scale from 0 to 100
**Scoring Procedure**	**Step 1: Transform Score**<br>Items are scored and linearly transformed to a 0-100 scale as follows: 1=0, 2=25, 3=50, 4=75, 5=100.<br><br>**Step 2: Calculate Scores by Dimensions**<br>• If 50% or more items are completed, the Mean score = Sum of the items divided by the number of items answered in the scale,<br>• If more than 50% of the items in the scale are missing, the scale scores should not be computed.<br><br>**Total Score**: Mean score = Sum of all the items in all scales divided by the number of items answered on all the scales.
**Interpretation and Analysis of Missing Data**	If more than 50% of the items in the scale are missing, the Scale Scores should not be computed.<br>If 50% or more items are completed: Impute the mean of the completed items in the scale.

PedsQL ™ Infant Scales

PARENT Report for Infants (ages 1-24 months)

The Parent Report for Infants (ages 1-24 months) of the **PedsQL**™ Infant Scales is composed of 36 items for infants ages 1-12 months and 45 items for infants ages 13-24 months comprising 5 dimensions.

DESCRIPTION OF THE QUESTIONNAIRE (INFANTS 1-12 MONTHS):

Dimensions	Number of Items	Cluster of Items	Reversed Scoring	Direction of Dimensions
Physical Functioning	6	1-6	1-6	Higher scores indicate better HRQOL.
Physical Symptoms	10	1-10	1-10	
Emotional Functioning	12	1-12	1-12	
Social Functioning	4	1-4	1-4	
Cognitive Functioning	4	1-4	1-4	

DESCRIPTION OF THE QUESTIONNAIRE (INFANTS 13-24 MONTHS):

Dimensions	Number of Items	Cluster of Items	Reversed Scoring	Direction of Dimensions
Physical Functioning	9	1-9	1-9	Higher scores indicate better HRQOL.
Physical Symptoms	10	1-10	1-10	
Emotional Functioning	12	1-12	1-12	
Social Functioning	5	1-5	1-5	
Cognitive Functioning	9	1-9	1-9	

SCORING OF DIMENSIONS:

**Item Scaling**	5-point Likert scale from 0 (Never) to 4 (Almost always)
**Weighting of Items**	No
**Extension of the Scoring Scale**	Scores are transformed on a scale from 0 to 100.

**Scoring Procedure**	**<u>Step 1: Transform Score</u>**<br>Items are reversed scored and linearly transformed to a 0-100 scale as follows: 0=100, 1=75, 2=50, 3=25, 4=0.<br><br>**<u>Step 2: Calculate Scores</u>**<br><u>Score by Dimensions:</u><br>• If more than 50% of the items in the scale are missing, the scale scores should not be computed.<br>• Mean score = Sum of the items over the number of items answered.<br><br><u>Psychosocial Health Summary Score</u> = Sum of the items over the number of items answered in the Emotional, Social, and Cognitive Functioning Scales.<br><br><u>Physical Health Summary Score</u> = Sum of the items over the number of items answered in the Physical Functioning and Physical Symptoms Scales.<br><br>**<u>Total Score</u>**: Sum of all the items over the number of items answered on all the Scales.
**Interpretation and Analysis of Missing Data**	If more than 50% of the items in the scale are missing, the Scale Scores should not be computed.<br><br>If 50% or more items are completed: Impute the mean of the completed items in a scale.

PedsQL ™ Multidimensional Fatigue Scale

Mapi Research Trust

The **PedsQL**TM Multidimensional Fatigue Scale is composed of 18 items comprising 3 dimensions.

DESCRIPTION OF THE MULTIDIMENSIONAL FATIGUE MODULE:

Dimensions	Number of Items	Cluster of Items	Reversed Scoring	Direction of Dimensions
General Fatigue	6	1-6	1-6	Higher scores indicate lower problems.
Sleep/Rest Fatigue	6	1-6	1-6	
Cognitive Fatigue	6	1-6	1-6	

SCORING OF DIMENSIONS:

**Item Scaling**	5-point Likert scale from 0 (Never) to 4 (Almost always)<br>3-point scale: 0 (Not at all), 2 (Sometimes) and 4 (A lot) for the Child Report for Young Children (ages 5-7)
**Weighting of Items**	No
**Extension of the Scoring Scale**	Scores are transformed on a scale from 0 to 100.
**Scoring Procedure**	**Step 1: Transform Score**<br>Items are reversed scored and linearly transformed to a 0-100 scale as follows: 0=100, 1=75, 2=50, 3=25, 4=0.<br><br>**Step 2: Calculate Scores by Dimensions**<br>• If more than 50% of the items in the scale are missing, the scale scores should not be computed,<br>• Mean score= Sum of the items over the number of items answered.<br><br>**Total Score**: Sum of all the items over the number of items answered on all the Scales
**Interpretation and Analysis of Missing Data**	If more than 50% of the items in the scale are missing, the Scale Scores should not be computed.<br>If 50% or more items are completed: Impute the mean of the completed items in a scale.

PedsQL ™ 3.0 Neurofibromatosis Module

CHILD and PARENT Reports for Young Children (ages 5-7), Children (ages 8-12), Teens (ages 13-18), Young Adults (ages 18-25), and Adults (ages over 26)

The **Child and Parent Reports** of the **PedsQL™ 3.0 Neurofibromatosis Module** for:
- Young Children (ages 5-7)
- Children (ages 8-12),
- Teens (ages 13-18),
- Young Adults (ages 18-25),
- Adults (ages over 26)

are composed of 104 items comprising 18 dimensions.

DESCRIPTION OF THE NEUROFIBROMATOSIS MODULE:

Dimensions	Number of Items	Cluster of Items	Reversed Scoring	Direction of Dimensions
Skin Itch Bother	6	1-6	1-6	Higher scores indicate lower problems.
Skin Sensations	3	1-3	1-3	
Pain	6	1-6	1-6	
Pain Impact	16	1-16	1-16	
Pain Management	2	1-2	1-2	
Cognitive Functioning	15	1-15	1-15	
Speech	4	1-4	1-4	
Fine Motor	6	1-6	1-6	
Balance	5	1-5	1-5	
Vision	5	1-5	1-5	
Perceived Physical Appearance	3	1-3	1-3	
Communication	6	1-6	1-6	
Worry	10	1-10	1-10	
Treatment	6	1-6	1-6	
Medicines	3	1-3	1-3	
Stomach Discomfort	3	1-3	1-3	
Constipaton	3	1-3	1-3	
Diarrhea	2	1-2	1-2	

SCORING OF DIMENSIONS:

**Item Scaling**	5-point Likert scale from 0 (Never) to 4 (Almost always)<br>3-point scale: 0 (Not at all), 2 (Sometimes) and 4 (A lot) for the Young Child (ages 5-7) child report
**Weighting of Items**	No
**Extension of the Scoring Scale**	Scores are transformed on a scale from 0 to 100.
**Scoring Procedure**	**Step 1: Transform Score**<br>Items are reversed scored and linearly transformed to a 0-100 scale as follows: 0=100, 1=75, 2=50, 3=25, 4=0.<br><br>**Step 2: Calculate Scores by Dimensions**<br>• If more than 50% of the items in the scale are missing, the scale scores should not be computed,<br>• Mean score = Sum of the items over the number of items answered.<br><br>**Total Score**: Sum of all the items over the number of items answered on all the Scales.
**Interpretation and Analysis of Missing Data**	If more than 50% of the items in the scale are missing, the Scale Scores should not be computed.<br>If 50% or more items are completed: Impute the mean of the completed items in a scale.

PedsQL ™ 3.0 Neuromuscular Module

Child and Parent Reports of the **PedsQL**™ 3.0 Neuromuscular Module for:

- Children (ages 8-12),
- And Teens (ages 13-18),

are composed of 25 items comprising 3 dimensions.

- **Young Child Report** (ages 5-7) only consists of the 17-item About My Neuromuscular Disease Scale, **Parent Report** for ages 2-18 includes all 3 dimensions and all 25 items,

DESCRIPTION OF THE NEUROMUSCULAR MODULE:

Dimensions	Number of Items	Cluster of Items	Reversed Scoring	Direction of Dimensions
About My Neuromuscular Disease	17	1-17	1-17	Higher scores indicate lower problems.
Communication	3	1-3	1-3	
About Our Family Resources	5	1-5	1-5	

SCORING OF DIMENSIONS:

**Item Scaling**	5-point Likert scale from 0 (Never) to 4 (Almost always)
**Weighting of Items**	No
**Extension of the Scoring Scale**	Scores are transformed on a scale from 0 to 100.
**Scoring Procedure**	**Step 1: Transform Score**<br>Items are reversed scored and linearly transformed to a 0-100 scale as follows: 0=100, 1=75, 2=50, 3=25, 4=0.<br><br>**Step 2: Calculate Scores by Dimensions**<br>• If more than 50% of the items in the scale are missing, the scale scores should not be computed,<br>• Mean score = Sum of the items over the number of items answered.<br><br>**Total Score**: Sum of all the items over the number of items answered on all the Scales.
**Interpretation and Analysis of missing data**	If more than 50% of the items in the scale are missing, the Scale Scores should not be computed.<br>If 50% or more items are completed: Impute the mean of the completed items in a scale.

PedsQL ™ Oral Health Scale

The **PedsQL^{TM}** Oral Health Scale is composed of 5 items comprising 1 dimension.

DESCRIPTION OF THE ORAL HEALTH SCALE:

Dimensions	Number of Items	Cluster of Items	Reversed Scoring	Direction of Dimensions
Oral Health	5	1-5	1-5	Higher scores indicate lower problems

SCORING OF DIMENSIONS:

**Item Scaling**	5-point Likert scale from 0 (Never) to 4 (Almost always)<br>3-point scale: 0 (Not at all), 2 (Sometimes) and 4 (A lot) for the Child Report for Young Children (ages 5-7)
**Weighting of Items**	No
**Extension of the Scoring Scale**	Scores are transformed on a scale from 0 to 100.
**Scoring Procedure**	**Step 1: Transform Score**<br>Items are reversed scored and linearly transformed to a 0-100 scale as follows: 0=100, 1=75, 2=50, 3=25, 4=0.<br>**Step 2: Calculate Total Score**<br>Sum of all the items over the number of items answered
**Interpretation and Analysis of Missing Data**	If more than 50% of the items in the scale are missing, the Scale Score should not be computed.<br>If 50% or more items are completed: Impute the mean of the completed items in a scale.

PedsQL ™ Pediatric Pain Coping Inventory™ (PPCI)

PARENT Report for Children and Teens (ages 5-18),
CHILD Report for Children (ages 5-12), Teens (ages 13-18)

The Parent Report of the **PedsQL™ Pediatric Pain Coping Inventory™** for Children (ages 5-12) and Teens (ages 13-18) is composed of 41 items comprising 5 dimensions.

The Child Reports of the **PedsQL™ Pediatric Pain Coping Inventory™** for:
- Children (ages 5-12)
- And teens (ages 13-18)

are composed of 41 items comprising 5 dimensions.

See Varni, J.W. et al. (1996). Development of the Waldron/Varni Pediatric Pain Coping Inventory. Pain, 67, 141-150, for scoring instructions for the *a priori* and *empirically-derived* scale scores.

DESCRIPTION OF THE PEDIATRIC PAIN COPING INVENTORY:

Dimensions	Number of Items	Cluster of Items	Reversed Scoring	Direction of Dimensions
Cognitive Self-Instruction	7	12, 22, 23, 24, 31, 32, 40	No	Higher scores indicate greater use of the coping strategy.
Problem-Solving	10	1, 2, 11, 15, 26, 27, 30, 33, 34, 35		
Distraction	9	6, 8, 9, 10, 14, 16, 17, 18, 19		
Seeks Social Support	9	3, 4, 7, 13, 28, 29, 37, 38 ,39		
Catastrophizing / Helplessness	6	5, 20, 21, 25, 36, 41		

SCORING OF DIMENSIONS:

**Item Scaling**	3-point Likert scale from 0 (Never) to 2 (Often)
**Weighting of Items**	No
**Extension of the Scoring Scale**	Scores are transformed on a scale from 0 to 2.
**Scoring Procedure**	**Calculate Scale Scores**<br>Sum of all the items over the number of items answered for each Scale.
**Interpretation and Analysis of Missing Data**	If more than 50% of the items in the scale are missing, the Scale Scores should not be computed.<br><br>If 50% or more items are completed: Impute the mean of the completed items in a scale.

PedsQL ™ Pediatric Pain Questionnaire ™ (PPQ)

The **PedsQL™** Pediatric Pain Questionnaire™ is composed of 3 items.

DESCRIPTION OF THE QUESTIONNAIRE:

Dimensions	Number of Items	Cluster of Items	Reversed Scoring	Direction of Dimensions
Present Pain	1	1	No	Higher score = higher pain intensity.
Worst Pain	1	2		

The third item refers to the localisation of pain and is not scored.

SCORING OF DIMENSIONS:

**Item Scaling**	10cm (100mm) VAS scales from 0 (Not hurting / No discomfort / No pain) to 10 (100) (Hurting a whole lot / Very uncomfortable / Severe pain)
**Weighting of Items**	No
**Extension of the Scoring Scale**	0-10cm or 0-100mm
**Scoring Procedure**	Present pain and worst pain are scored separately.<br>The score is based on line length to the nearest 0.5 cm (5mm).
**Interpretation and Analysis of Missing Data**	There is no imputation process since these are single item VAS scales.

PedsQL ™ Present Functioning Visual Analogue Scale (PedsQL ™ VAS)

CHILD and PARENT reports for CHILD (ages 5-18)

The **PedsQL™** Present Functioning Visual Analogue Scale is composed of 6 items.

DESCRIPTION OF THE SCALE:

Dimensions	Number of Items	Cluster of Items	Reversed Scoring	Direction of Dimensions
Present Functioning	6	1-6	No	Higher score = higher distress or intensity.

SCORING OF DIMENSIONS:

**Item Scaling**	100mm VAS scales from 0 to 100
**Weighting of Items**	No
**Extension of the Scoring Scale**	0-100
**Scoring Procedure**	- PedsQL VAS Total Symptom Score = average of all six items<br>- PedsQL VAS Emotional Distress Summary Score = mean of the anxiety, sadness, anger, and worry items
**Interpretation and Analysis of Missing Data**	There is no imputation process since these are single item VAS scales.

PedsQL TM 3.0 Rheumatology Module

PARENT Report for Toddlers (ages 2-4)

The **Parent Report for Toddlers** (age 2-4) of the **PedsQL™** 3.0 Rheumatology Module is composed of 14 items comprising 3 dimensions.

DESCRIPTION OF THE RHEUMATOLOGY MODULE:

Dimensions	Number of Items	Cluster of Items	Reversed Scoring	Direction of Dimensions
Pain and Hurt	4	1-4	1-4	Higher scores indicate lower problems.
Daily Activities	5	1-5	1-5	
Treatment	5	1-5	1-5	

SCORING OF DIMENSIONS:

**Item Scaling**	5-point Likert scale from 0 (Never) to 4 (Almost always)
**Weighting of Items**	No
**Extension of the Scoring Scale**	Scores are transformed to a 0 to 100 scale.
**Scoring Procedure**	**Step 1: Transform Score**<br>Items are reversed scored and linearly transformed to a 0-100 scale as follows: 0=100, 1=75, 2=50, 3=25, 4=0.<br>**Step 2: Calculate Scores by Dimensions**<br>• If more than 50% of the items in the scale are missing, the scale scores should not be computed,<br>• Mean score = Sum of the items over the number of items answered.<br>**There is no Total Score**
**Interpretation and Analysis of Missing Data**	If more than 50% of the items in the scale are missing, the Scale Scores should not be computed.<br>If 50% or more items are completed: Impute the mean of the completed items in a scale.

CHILD and PARENT reports for Young Children (ages 5-7)

The **Child and Parent Reports** of the **PedsQL™** 3.0 Rheumatology Module for Young Children (ages 5-7) are composed of 20 items comprising 5 dimensions.

DESCRIPTION OF THE RHEUMATOLOGY MODULE:

Dimensions	Number of Items	Cluster of Items	Reversed Scoring	Direction of Dimensions
Pain and Hurt	4	1-4	1-4	Higher scores indicate lower problems.
Daily Activities	5	1-5	1-5	
Treatment	5	1-5	1-5	
Worry	3	1-3	1-3	
Communication	3	1-3	1-3	

SCORING OF DIMENSIONS:

**Item Scaling**	5-point Likert scale from 0 (Never) to 4 (Almost always)<br>3-point scale: 0 (Not at all), 2 (Sometimes) and 4 (A lot) for the child report for Young Children (ages 5-7)
**Weighting of Items**	No
**Extension of the Scoring Scale**	Scores are transformed on a scale from 0 to 100.
**Scoring Procedure**	**Step 1: Transform Score**<br>Items are reversed scored and linearly transformed to a 0-100 scale as follows: 0=100, 1=75, 2=50, 3=25, 4=0.<br><br>**Step 2: Calculate Scores by Dimensions**<br>• If more than 50% of the items in the scale are missing, the scale scores should not be computed,<br>• Mean score = Sum of the items over the number of items answered.<br><br>**There is no Total Score**
**Interpretation and Analysis of Missing Data**	If more than 50% of the items in the scale are missing, the Scale Scores should not be computed.<br>If 50% or more items are completed: Impute the mean of the completed items in a scale.

CHILD and PARENT reports for Children (ages 8-12) and Teens (ages 13-18)

The **Child and Parent Reports** of the **PedsQL™** 3.0 Rheumatology Module for:
- Children (ages 8-12),
- And Teens (ages 13-18)

are composed of 22 items comprising 5 dimensions.

DESCRIPTION OF THE RHEUMATOLOGY MODULE:

Dimensions	Number of Items	Cluster of Items	Reversed Scoring	Direction of Dimensions
Pain and Hurt	4	1-4	1-4	Higher scores indicate lower problems.
Daily Activities	5	1-5	1-5	
Treatment	7	1-5	1-5	
Worry	3	1-3	1-3	
Communication	3	1-3	1-3	

SCORING OF DIMENSIONS:

**Item Scaling**	5-point Likert scale from 0 (Never) to 4 (Almost always)
**Weighting of Items**	No
**Extension of the Scoring Scale**	Scores are transformed on a scale from 0 to 100.
**Scoring Procedure**	**Step 1: Transform Score**<br>Items are reversed scored and linearly transformed to a 0-100 scale as follows: 0=100, 1=75, 2=50, 3=25, 4=0.<br><br>**Step 2: Calculate Scores by Dimensions**<br>• If more than 50% of the items in the scale are missing, the scale scores should not be computed,<br>• Mean score = Sum of the items over the number of items answered.<br><br>**There is no Total Score**
**Interpretation and Analysis of Missing Data**	If more than 50% of the items in the scale are missing, the Scale Scores should not be computed.<br>If 50% or more items are completed: Impute the mean of the completed items in a scale.

PedsQL [TM] 3.0 Sickle Cell Disease Module

PARENT Report for Toddlers (ages 2-4)

The Parent Report for Toddlers (age 2-4) of the **PedsQL™** 3.0 Sickle Cell Disease Module is composed of 42 items comprising 9 dimensions.

DESCRIPTION OF THE SICKLE CELL DISEASE MODULE:

Dimensions	Number of Items	Cluster of Items	Reversed Scoring	Direction of Dimensions
Pain and Hurt	9	1-9	1-9	Higher scores indicate lower problems.
Pain Impact	10	1-10	1-10	
Pain Management	2	1-2	1-2	
Worry I	5	1-5	1-5	
Worry II	2	1-2	1-2	
Emotions	2	1-2	1-2	
Treatment	6	1-6	1-6	
Communication I	3	1-3	1-3	
Communication II	3	1-3	1-3	

SCORING OF DIMENSIONS:

**Item Scaling**	5-point Likert scale from 0 (Never) to 4 (Almost always)
**Weighting of Items**	No
**Extension of the Scoring Scale**	Scores are transformed to a 0 to 100 scale.
**Scoring Procedure**	**Step 1: Transform Score**<br>Items are reversed scored and linearly transformed to a 0-100 scale as follows: 0=100, 1=75, 2=50, 3=25, 4=0.<br><br>**Step 2: Calculate scores by Dimensions**<br>• If more than 50% of the items in the scale are missing, the scale scores should not be computed.<br>• Mean score = Sum of the items over the number of items answered.<br><br>**Total Score:** Sum of all the items over the number of items answered on all the Scales.
**Interpretation and Analysis of Missing Data**	If more than 50% of the items in the scale are missing, the Scale Scores should not be computed.<br>If 50% or more items are completed: Impute the mean of the completed items in a scale.

CHILD and PARENT Reports for Young Children (ages 5-7)

The **Child and Parent Reports for Young Children (ages 5-7)** of the **PedsQL™** 3.0 Sickle Cell Disease Module are composed of 40 items for the child report and 42 items for the parent report, comprising 9 dimensions.

DESCRIPTION OF THE SICKLE CELL DISEASE MODULE:

Dimensions	Number of Items	Cluster of Items	Reversed Scoring	Direction of Dimensions
Pain and Hurt	9	1-9	1-9	Higher scores indicate lower problems.
Pain Impact	10	1-10	1-10	
Pain Management	2	1-2	1-2	
Worry I	5	1-5	1-5	
Worry II*	2	1-2	1-2	
Emotions	2	1-2	1-2	
Treatment	6	1-6	1-6	
Communication I	3	1-3	1-3	
Communication II	3	1-3	1-3	

*Note: Worry II Scale is not included for child self-report for ages 5-7.

SCORING OF DIMENSIONS:

**Item Scaling**	5-point Likert scale from 0 (Never) to 4 (Almost always)<br>3-point scale: 0 (Not at all), 2 (Sometimes) and 4 (A lot) for the Child Report for Young Children (ages 5-7)
**Weighting of Items**	No
**Extension of the Scoring Scale**	Scores are transformed on a scale from 0 to 100.
**Scoring Procedure**	**Step 1: Transform Score**<br>Items are reversed scored and linearly transformed to a 0-100 scale as follows: 0=100, 1=75, 2=50, 3=25, 4=0<br>**Step 2: Calculate Scores by Dimensions**<br>• If more than 50% of the items in the scale are missing, the scale scores should not be computed.<br>• Mean score = Sum of the items over the number of items answered.<br>**Total Score:** Sum of all the items over the number of items answered on all the Scales.
**Interpretation and Analysis of Missing Data**	If more than 50% of the items in the scale are missing, the Scale Scores should not be computed.<br>If 50% or more items are completed: Impute the mean of the completed items in a scale.

CHILD and PARENT Reports for Children (ages 8-12),Teens (ages 13-18), Young Adults (ages 18-25), and Adults (ages over 26)

The **Child and Parent Reports** of the **PedsQL™** 3.0 Sickle Cell Disease Module for:
- Children (ages 8-12)
- Teens (ages 13-18)
- Young Adults (ages 18-25)
- And Adults (ages over 26)

are composed of 43 items comprising 9 dimensions.

DESCRIPTION OF THE SICKLE CELL DISEASE MODULE:

Dimensions	Number of Items	Cluster of Items	Reversed Scoring	Direction of Dimensions
Pain and Hurt	9	1-9	1-9	Higher scores indicate lower problems.
Pain Impact	10	1-10	1-10	
Pain Management	2	1-2	1-2	
Worry I	5	1-5	1-5	
Worry II	2	1-2	1-2	
Emotions	2	1-2	1-2	
Treatment	7	1-7	1-7	
Communication I	3	1-3	1-3	
Communication II	3	1-3	1-3	

SCORING OF DIMENSIONS:

**Item Scaling**	5-point Likert scale from 0 (Never) to 4 (Almost always)
**Weighting of Items**	No
**Extension of the Scoring Scale**	Scores are transformed on a scale from 0 to 100.
**Scoring Procedure**	**Step 1: Transform Score**<br>Items are reversed scored and linearly transformed to a 0-100 scale as follows: 0=100, 1=75, 2=50, 3=25, 4=0<br><br>**Step 2: Calculate Scores by Dimensions**<br>• If more than 50% of the items in the scale are missing, the scale scores should not be computed.<br>• Mean score = Sum of the items over the number of items answered.<br><br>**Total Score:** Sum of all the items over the number of items answered on all the Scales.
**Interpretation and Analysis of Missing Data**	If more than 50% of the items in the scale are missing, the Scale Scores should not be computed.<br>If 50% or more items are completed: Impute the mean of the completed items in a scale.

PedsQL ™ 1.0 Stem Cell Transplant Module

PARENT Report for Toddlers (ages 2-4)

The Parent Report for Toddlers (age 2-4) of the **PedsQL™** 1.0 Stem Cell Transplant Module is composed of 31 items comprising 8 dimensions.

DESCRIPTION OF THE STEM CELL TRANSPLANT MODULE:

Dimensions	Number of Items	Cluster of Items	Reversed Scoring	Direction of Dimensions
Pain and Hurt	2	1-2	1-2	Higher scores indicate lower problems.
Fatigue and Sleep	5	1-5	1-5	
Nausea	4	1-4	1-4	
Worry	2	1-2	1-2	
Nutrition	5	1-5	1-5	
Thinking	4	1-4	1-4	
Communication	3	1-3	1-3	
Other Complaints	6	1-6	1-6	

SCORING OF DIMENSIONS:

**Item Scaling**	5-point Likert scale from 0 (Never) to 4 (Almost always)
**Weighting of Items**	No
**Extension of the Scoring Scale**	Scores are transformed to a 0 to 100 scale.
**Scoring Procedure**	**Step 1: Transform Score**<br>Items are reversed scored and linearly transformed to a 0-100 scale as follows: 0=100, 1=75, 2=50, 3=25, 4=0.<br><br>**Step 2: Calculate scores by Dimensions**<br>• If more than 50% of the items in the scale are missing, the scale scores should not be computed.<br>• Mean score = Sum of the items over the number of items answered.<br><br>**Total Score:** Sum of all the items over the number of items answered on all the Scales.
**Interpretation and Analysis of Missing Data**	If more than 50% of the items in the scale are missing, the Scale Scores should not be computed.<br>If 50% or more items are completed: Impute the mean of the completed items in a scale.

PARENT Report for Young Children (ages 5-7)

The **Parent Report for Young Children (ages 5-7)** of the **PedsQL™** 1.0 Stem Cell Transplant Module is composed of 39 items comprising 8 dimensions.

DESCRIPTION OF THE STEM CELL TRANSPLANT MODULE:

Dimensions	Number of Items	Cluster of Items	Reversed Scoring	Direction of Dimensions
Pain and Hurt	2	1-2	1-2	Higher scores indicate lower problems.
Fatigue and Sleep	5	1-5	1-5	
Nausea	4	1-4	1-4	
Worry	10	1-10	1-10	
Nutrition	5	1-5	1-5	
Thinking	4	1-4	1-4	
Communication	3	1-3	1-3	
Other Complaints	6	1-6	1-6	

SCORING OF DIMENSIONS:

**Item Scaling**	5-point Likert scale from 0 (Never) to 4 (Almost always)
**Weighting of Items**	No
**Extension of the Scoring Scale**	Scores are transformed on a scale from 0 to 100.
**Scoring Procedure**	**Step 1: Transform Score**<br>Items are reversed scored and linearly transformed to a 0-100 scale as follows: 0=100, 1=75, 2=50, 3=25, 4=0<br><br>**Step 2: Calculate Scores by Dimensions**<br>• If more than 50% of the items in the scale are missing, the scale scores should not be computed.<br>• Mean score = Sum of the items over the number of items answered.<br><br>**Total Score:** Sum of all the items over the number of items answered on all the Scales.
**Interpretation and Analysis of Missing Data**	If more than 50% of the items in the scale are missing, the Scale Scores should not be computed.<br>If 50% or more items are completed: Impute the mean of the completed items in a scale.

CHILD and PARENT Reports for Children (ages 8-12)

The **Child and Parent Reports for Children (ages 8-12)** of the **PedsQL^{TM}** 1.0 Stem Cell Transplant Module are composed of 39 items comprising 8 dimensions.

DESCRIPTION OF THE STEM CELL TRANSPLANT MODULE:

Dimensions	Number of Items	Cluster of Items	Reversed Scoring	Direction of Dimensions
Pain and Hurt	2	1-2	1-2	Higher scores indicate lower problems.
Fatigue and Sleep	5	1-5	1-5	
Nausea	4	1-4	1-4	
Worry	10	1-10	1-10	
Nutrition	5	1-5	1-5	
Thinking	4	1-4	1-4	
Communication	3	1-3	1-3	
Other Complaints	6	1-6	1-6	

SCORING OF DIMENSIONS:

**Item Scaling**	5-point Likert scale from 0 (Never) to 4 (Almost always)
**Weighting of Items**	No
**Extension of the Scoring Scale**	Scores are transformed on a scale from 0 to 100.
**Scoring Procedure**	**Step 1: Transform Score**<br>Items are reversed scored and linearly transformed to a 0-100 scale as follows: 0=100, 1=75, 2=50, 3=25, 4=0<br><br>**Step 2: Calculate Scores by Dimensions**<br>• If more than 50% of the items in the scale are missing, the scale scores should not be computed.<br>• Mean score = Sum of the items over the number of items answered.<br><br>**Total Score:** Sum of all the items over the number of items answered on all the Scales.
**Interpretation and Analysis of Missing Data**	If more than 50% of the items in the scale are missing, the Scale Scores should not be computed.<br>If 50% or more items are completed: Impute the mean of the completed items in a scale.

TEEN and PARENT Reports for Teens (ages 13-18)

The **Teen and Parent Reports for Teens (ages 13-18)** of the **PedsQL™** 1.0 Stem Cell Transplant Module are composed of 41 items comprising 8 dimensions.

DESCRIPTION OF THE STEM CELL TRANSPLANT MODULE:

Dimensions	Number of Items	Cluster of Items	Reversed Scoring	Direction of Dimensions
Pain and Hurt	2	1-2	1-2	Higher scores indicate lower problems.
Fatigue and Sleep	5	1-5	1-5	
Nausea	4	1-4	1-4	
Worry	12	1-12	1-12	
Nutrition	5	1-5	1-5	
Thinking	4	1-4	1-4	
Communication	3	1-3	1-3	
Other Complaints	6	1-6	1-6	

SCORING OF DIMENSIONS:

**Item Scaling**	5-point Likert scale from 0 (Never) to 4 (Almost always)
**Weighting of Items**	No
**Extension of the Scoring Scale**	Scores are transformed on a scale from 0 to 100.
**Scoring Procedure**	**Step 1: Transform Score**<br>Items are reversed scored and linearly transformed to a 0-100 scale as follows: 0=100, 1=75, 2=50, 3=25, 4=0<br><br>**Step 2: Calculate Scores by Dimensions**<br>• If more than 50% of the items in the scale are missing, the scale scores should not be computed.<br>• Mean score = Sum of the items over the number of items answered.<br><br>**Total Score:** Sum of all the items over the number of items answered on all the Scales.
**Interpretation and Analysis of Missing Data**	If more than 50% of the items in the scale are missing, the Scale Scores should not be computed.<br>If 50% or more items are completed: Impute the mean of the completed items in a scale.

PedsQL TM 3.0 Transplant Module

The PedsQLTM 3.0 Transplant Module is composed of 46 items comprising 8 dimensions.

DESCRIPTION OF THE TRANSPLANT MODULE:

Dimensions	Number of Items	Cluster of Items	Reversed Scoring	Direction of Dimensions
About My Medicines I	9	1-9	1-9	Higher scores indicate lower problems.
About My Medicines II	8	1-8	1-8	
My Transplant and Others	8	1-8	1-8	
Pain and Hurt	3	1-3	1-3	
Worry	7	1-7	1-7	
Treatment Anxiety	4	1-4	1-4	
How I Look	3	1-3	1-3	
Communication	4	1-4	1-4	

SCORING OF DIMENSIONS:

**Item Scaling**	5-point scale from 0 (Never) to 4 (Almost always)<br>3-point scale: 0 (Not at all), 2 (Sometimes) and 4 (A lot) for the Child Report for Young Children (ages 5-7)
**Weighting of Items**	No
**Extension of the Scoring Scale**	Scores are transformed on a scale from 0 to 100.
**Scoring Procedure**	**<u>Step 1: Transform Score</u>**<br>Items are reversed scored and linearly transformed to a 0-100 scale as follows: 0=100, 1=75, 2=50, 3=25, 4=0.<br>**<u>Step 2: Calculate scores by Dimension</u>**<br>• If more than 50% of the items in the scale are missing, the scale scores should not be computed,<br>• Mean score = Sum of the items over the number of items answered.<br>**<u>Total Score</u>**: Sum of all the items over the number of items answered on all the Scales.
**Interpretation and Analysis of Missing Data**	If more than 50% of the items in the scale are missing, the Scale Scores should not be computed.<br>If 50% or more items are completed: Impute the mean of the completed items in a scale.
