# Supplementary material for: Effects of minimally invasive and traditional surgeries on the quality of life of children with congenital heart disease: a retrospective propensity score-matched study
Source: BMC Pediatr. 2021 Nov 24;21:522. doi: 10.1186/s12887-021-02978-5 (PMC8611858; doi:10.1186/s12887-021-02978-5)
Supplement: Supplementary file 9 — Additional file 9. [file 12887_2021_2978_MOESM9_ESM.pdf]

ID# ______________________

Date: ______________________

# PedsQL™

## Cardiac Module

Version 3.0

**TEEN REPORT** (ages **13-18**)

**DIRECTIONS**

Teens with heart conditions sometimes have special problems.
Please tell us **how much of a problem** each one has been for you during the **past ONE month** by circling:

**0** if it is **never** a problem
**1** if it is **almost never** a problem
**2** if it is **sometimes** a problem
**3** if it is **often** a problem
**4** if it is **almost always** a problem

There are no right or wrong answers.
If you do not understand a question, please ask for help.


Not to be reproduced without permission
Copyright © 1998 JW Varni, Ph.D.
All rights reserved

*In the past **ONE** month, how much of a **problem** has this been for you…*

| **HEART PROBLEMS AND TREATMENT** ***(problems with…)*** | **Never** | **Almost Never** | **Some-times** | **Often** | **Almost Always** |
|---|---|---|---|---|---|
| 1. I get out of breath when I do sports activity or exercise | 0 | 1 | 2 | 3 | 4 |
| 2. My chest hurts or feels tight when I do sports activity or exercise | 0 | 1 | 2 | 3 | 4 |
| 3. I catch colds easily | 0 | 1 | 2 | 3 | 4 |
| 4. I feel my heart beating fast | 0 | 1 | 2 | 3 | 4 |
| 5. My lips turn blue when I run | 0 | 1 | 2 | 3 | 4 |
| 6. I wake up at night with trouble breathing | 0 | 1 | 2 | 3 | 4 |
| 7. I have to rest more than my friends | 0 | 1 | 2 | 3 | 4 |

**If you are currently taking heart medicine, please answer the following…**
**Otherwise, please skip to "Perceived Physical Appearance".**

| **TREATMENT II** ***(problems with…)*** | **Never** | **Almost Never** | **Some-times** | **Often** | **Almost Always** |
|---|---|---|---|---|---|
| 1. I refuse to take my heart medicine | 0 | 1 | 2 | 3 | 4 |
| 2. It is hard for me to take my heart medicine | 0 | 1 | 2 | 3 | 4 |
| 3. I forget to take my heart medicine | 0 | 1 | 2 | 3 | 4 |
| 4. My heart medicine makes me feel sick | 0 | 1 | 2 | 3 | 4 |
| 5. I worry about side effects from my medicine | 0 | 1 | 2 | 3 | 4 |

| **PERCEIVED PHYSICAL APPEARANCE** ***(problems with…)*** | **Never** | **Almost Never** | **Some-times** | **Often** | **Almost Always** |
|---|---|---|---|---|---|
| 1. I feel I am not good looking | 0 | 1 | 2 | 3 | 4 |
| 2. I don't like other people to see my scars | 0 | 1 | 2 | 3 | 4 |
| 3. I am embarrassed when others see my body | 0 | 1 | 2 | 3 | 4 |

| **TREATMENT ANXIETY** ***(problems with…)*** | **Never** | **Almost Never** | **Some-times** | **Often** | **Almost Always** |
|---|---|---|---|---|---|
| 1. I get scared when I am waiting to see the doctor | 0 | 1 | 2 | 3 | 4 |
| 2. I get scared when I have to go to the doctor | 0 | 1 | 2 | 3 | 4 |
| 3. I get scared when I have to go to the hospital | 0 | 1 | 2 | 3 | 4 |
| 4. I get scared when I have to have medical treatments | 0 | 1 | 2 | 3 | 4 |


Not to be reproduced without permission
Copyright © 1998 JW Varni, Ph.D.
All rights reserved

*In the past **ONE** month, how much of a **problem** has this been for you…*

| **COGNITIVE PROBLEMS *(problems with…)*** | **Never** | **Almost Never** | **Some-times** | **Often** | **Almost Always** |
|---|---|---|---|---|---|
| 1. It is hard for me to figure out what to do when something bothers me | 0 | 1 | 2 | 3 | 4 |
| 2. I have trouble solving math problems | 0 | 1 | 2 | 3 | 4 |
| 3. I have trouble writing school papers or reports | 0 | 1 | 2 | 3 | 4 |
| 4. It is hard for me to pay attention to things | 0 | 1 | 2 | 3 | 4 |
| 5. It is hard for me to remember what I read | 0 | 1 | 2 | 3 | 4 |

| **COMMUNICATION *(problems with…)*** | **Never** | **Almost Never** | **Some-times** | **Often** | **Almost Always** |
|---|---|---|---|---|---|
| 1. It is hard for me to tell the doctors and nurses how I feel | 0 | 1 | 2 | 3 | 4 |
| 2. It is hard for me to ask the doctors and nurses questions | 0 | 1 | 2 | 3 | 4 |
| 3. It is hard for me to explain my heart problem to other people | 0 | 1 | 2 | 3 | 4 |


Not to be reproduced without permission
Copyright © 1998 JW Varni, Ph.D.
All rights reserved
