# Supplementary material for: Effects of minimally invasive and traditional surgeries on the quality of life of children with congenital heart disease: a retrospective propensity score-matched study
Source: BMC Pediatr. 2021 Nov 24;21:522. doi: 10.1186/s12887-021-02978-5 (PMC8611858; doi:10.1186/s12887-021-02978-5)
Supplement: Supplementary file 11 — Additional file 11. [file 12887_2021_2978_MOESM11_ESM.pdf]

ID#: ____________________

Date: ____________________

PedsQL™

Cardiac Module

Version 3.0

ADULT REPORT

DIRECTIONS

Adults with heart conditions sometimes have special problems.
Please tell us **how much of a problem** each one has been for you during the **past ONE month** by circling:

0 if it is **never** a problem
1 if it is **almost never** a problem
2 if it is **sometimes** a problem
3 if it is **often** a problem
4 if it is **almost always** a problem

There are no right or wrong answers.
If you do not understand a question, please ask for help.

Not to be reproduced without permission

Copyright © 1998 JW Varni, Ph.D.
All rights reserved

*In the past **ONE** month, how much of a **problem** has this been for you…*

**HEART PROBLEMS AND TREATMENT** ***(problems with…)***	**Never**	**Almost Never**	**Some-times**	**Often**	**Almost Always**
1. I get out of breath when I do sports activity or exercise	0	1	2	3	4
2. My chest hurts or feels tight when I do sports activity or exercise	0	1	2	3	4
3. I catch colds easily	0	1	2	3	4
4. I feel my heart beating fast	0	1	2	3	4
5. My lips turn blue when I run	0	1	2	3	4
6. I wake up at night with trouble breathing	0	1	2	3	4
7. I have to rest more than my friends	0	1	2	3	4

If you are currently taking heart medicine, please answer the following…
Otherwise, please skip to "Perceived Physical Appearance".

**TREATMENT II** ***(problems with…)***	**Never**	**Almost Never**	**Some-times**	**Often**	**Almost Always**
1. I refuse to take my heart medicine	0	1	2	3	4
2. It is hard for me to take my heart medicine	0	1	2	3	4
3. I forget to take my heart medicine	0	1	2	3	4
4. My heart medicine makes me feel sick	0	1	2	3	4
5. I worry about how my medicines affect my body	0	1	2	3	4

**PERCEIVED PHYSICAL APPEARANCE** ***(problems with…)***	**Never**	**Almost Never**	**Some-times**	**Often**	**Almost Always**
1. I feel I am not good looking	0	1	2	3	4
2. I don't like other people to see my scars	0	1	2	3	4
3. I am embarrassed when others see my body	0	1	2	3	4

**TREATMENT ANXIETY** ***(problems with…)***	**Never**	**Almost Never**	**Some-times**	**Often**	**Almost Always**
1. I get scared when I am waiting to see the doctor	0	1	2	3	4
2. I get scared when I have to go to the doctor	0	1	2	3	4
3. I get scared when I have to go to the hospital	0	1	2	3	4
4. I get scared when I have to have medical treatments	0	1	2	3	4

Not to be reproduced without permission
Copyright © 1998 JW Varni, Ph.D.
All rights reserved

*In the past **ONE** month, how much of a **problem** has this been for you…*

COGNITIVE PROBLEMS ***(problems with…)***	Never	Almost Never	Some-times	Often	Almost Always
1. It is hard for me to figure out what to do when something bothers me	0	1	2	3	4
2. I have trouble solving math problems	0	1	2	3	4
3. I have trouble writing papers or reports	0	1	2	3	4
4. It is hard for me to pay attention to things	0	1	2	3	4
5. It is hard for me to remember what I read	0	1	2	3	4

COMMUNICATION ***(problems with…)***	Never	Almost Never	Some-times	Often	Almost Always
1. It is hard for me to tell the doctors and nurses how I feel	0	1	2	3	4
2. It is hard for me to ask the doctors and nurses questions	0	1	2	3	4
3. It is hard for me to explain my heart problem to other people	0	1	2	3	4

Not to be reproduced without permission
Copyright © 1998 JW Varni, Ph.D.
All rights reserved

ID#: ____________________________

Date: ____________________________

PedsQL™

Cardiac Module

Version 3.0

YOUNG ADULT REPORT (ages 18-25)

DIRECTIONS

Young Adults with heart conditions sometimes have special problems. Please tell us **how much of a problem** each one has been for you during the **past ONE month** by circling:

0 if it is **never** a problem
1 if it is **almost never** a problem
2 if it is **sometimes** a problem
3 if it is **often** a problem
4 if it is **almost always** a problem

There are no right or wrong answers.
If you do not understand a question, please ask for help.

Not to be reproduced without permission
Copyright © 1998 JW Varni, Ph.D.
All rights reserved

*In the past **ONE** month, how much of a **problem** has this been for you…*

**HEART PROBLEMS AND TREATMENT** ***(problems with…)***	**Never**	**Almost Never**	**Some-times**	**Often**	**Almost Always**
1. I get out of breath when I do sports activity or exercise	0	1	2	3	4
2. My chest hurts or feels tight when I do sports activity or exercise	0	1	2	3	4
3. I catch colds easily	0	1	2	3	4
4. I feel my heart beating fast	0	1	2	3	4
5. My lips turn blue when I run	0	1	2	3	4
6. I wake up at night with trouble breathing	0	1	2	3	4
7. I have to rest more than my friends	0	1	2	3	4

If you are currently taking heart medicine, please answer the following…
Otherwise, please skip to "Perceived Physical Appearance".

**TREATMENT II** ***(problems with…)***	**Never**	**Almost Never**	**Some-times**	**Often**	**Almost Always**
1. I refuse to take my heart medicine	0	1	2	3	4
2. It is hard for me to take my heart medicine	0	1	2	3	4
3. I forget to take my heart medicine	0	1	2	3	4
4. My heart medicine makes me feel sick	0	1	2	3	4
5. I worry about how my medicines affect my body	0	1	2	3	4

**PERCEIVED PHYSICAL APPEARANCE** ***(problems with…)***	**Never**	**Almost Never**	**Some-times**	**Often**	**Almost Always**
1. I feel I am not good looking	0	1	2	3	4
2. I don't like other people to see my scars	0	1	2	3	4
3. I am embarrassed when others see my body	0	1	2	3	4

**TREATMENT ANXIETY** ***(problems with…)***	**Never**	**Almost Never**	**Some-times**	**Often**	**Almost Always**
1. I get scared when I am waiting to see the doctor	0	1	2	3	4
2. I get scared when I have to go to the doctor	0	1	2	3	4
3. I get scared when I have to go to the hospital	0	1	2	3	4
4. I get scared when I have to have medical treatments	0	1	2	3	4

Not to be reproduced without permission
Copyright © 1998 JW Varni, Ph.D.
All rights reserved

*In the past **ONE** month, how much of a **problem** has this been for you…*

**COGNITIVE PROBLEMS *(problems with…)***	**Never**	**Almost Never**	**Some-times**	**Often**	**Almost Always**
1. It is hard for me to figure out what to do when something bothers me	0	1	2	3	4
2. I have trouble solving math problems	0	1	2	3	4
3. I have trouble writing papers or reports	0	1	2	3	4
4. It is hard for me to pay attention to things	0	1	2	3	4
5. It is hard for me to remember what I read	0	1	2	3	4

**COMMUNICATION *(problems with…)***	**Never**	**Almost Never**	**Some-times**	**Often**	**Almost Always**
1. It is hard for me to tell the doctors and nurses how I feel	0	1	2	3	4
2. It is hard for me to ask the doctors and nurses questions	0	1	2	3	4
3. It is hard for me to explain my heart problem to other people	0	1	2	3	4

Not to be reproduced without permission
Copyright © 1998 JW Varni, Ph.D.
All rights reserved

ID#:________________________

Date: ________________________

PedsQL™

Cardiac Module

Version 3.0

PARENT REPORT for YOUNG ADULTS (ages 18-25)

DIRECTIONS

Young Adults with heart conditions sometimes have special problems. On the following page is a list of things that might be a problem for **your child**. Please tell us **how much of a problem** each one has been for **your child** during the **past ONE month** by circling:

0 if it is **never** a problem
1 if it is **almost never** a problem
2 if it is **sometimes** a problem
3 if it is **often** a problem
4 if it is **almost always** a problem

There are no right or wrong answers.
If you do not understand a question, please ask for help.

Not to be reproduced without permission

Copyright © 1998 JW Varni, Ph.D.
All rights reserved

In the past ***ONE month,*** *how much of a* ***problem*** *has your child had with …*

**HEART PROBLEMS AND TREATMENT** ***(problems with…)***	**Never**	**Almost Never**	**Some-times**	**Often**	**Almost Always**
1. Getting out of breath while doing sports activity or exercise	0	1	2	3	4
2. Chest pain or tightness while doing sports activity or exercise	0	1	2	3	4
3. Catching colds easily	0	1	2	3	4
4. Fast heartbeat	0	1	2	3	4
5. His/her lips turning blue when running	0	1	2	3	4
6. Waking up at night with trouble breathing	0	1	2	3	4
7. Having to rest more than his/her friends	0	1	2	3	4

If your child is currently taking heart medicine, please answer the following…
Otherwise, please skip to "Perceived Physical Appearance".

**TREATMENT II** ***(problems with…)***	**Never**	**Almost Never**	**Some-times**	**Often**	**Almost Always**
1. Refusing to take heart medicine	0	1	2	3	4
2. Difficulty taking heart medicine	0	1	2	3	4
3. Forgetting to take heart medicine	0	1	2	3	4
4. Heart medicine making him/her feel sick	0	1	2	3	4
5. Worry about side effects from his/her medicine	0	1	2	3	4

**PERCEIVED PHYSICAL APPEARANCE** ***(problems with…)***	**Never**	**Almost Never**	**Some-times**	**Often**	**Almost Always**
1. Feeling that he/she is not good looking	0	1	2	3	4
2. Not liking other people to see his/her scars	0	1	2	3	4
3. Being embarrassed about others seeing his/her body	0	1	2	3	4

**TREATMENT ANXIETY** ***(problems with…)***	**Never**	**Almost Never**	**Some-times**	**Often**	**Almost Always**
1. Getting anxious when waiting to see the doctor	0	1	2	3	4
2. Getting anxious about going to the doctor	0	1	2	3	4
3. Getting anxious about going to the hospital	0	1	2	3	4
4. Getting anxious when he/she has to have medical treatments	0	1	2	3	4

Not to be reproduced without permission

Copyright © 1998 JW Varni, Ph.D.
All rights reserved

*In the past **ONE month,** how much of a **problem** has your child had with …*

**COGNITIVE PROBLEMS *(problems with…)***	**Never**	**Almost Never**	**Some-times**	**Often**	**Almost Always**
1. Figuring out what to do when something bothers him/her	0	1	2	3	4
2. Trouble solving math problems	0	1	2	3	4
3. Trouble writing papers or reports	0	1	2	3	4
4. Difficulty paying attention to things	0	1	2	3	4
5. Remembering what he/she reads	0	1	2	3	4

**COMMUNICATION *(problems with…)***	**Never**	**Almost Never**	**Some-times**	**Often**	**Almost Always**
1. Telling the doctors and nurses how he/she feels	0	1	2	3	4
2. Asking the doctors or nurses questions	0	1	2	3	4
3. Explaining his/her heart problem to other people	0	1	2	3	4

Not to be reproduced without permission

Copyright © 1998 JW Varni, Ph.D.
All rights reserved

ID# ___________________________

Date:__________________________

PedsQL™

Cardiac Module

Version 3.0

TEEN REPORT (ages **13-18**)

DIRECTIONS

Teens with heart conditions sometimes have special problems.
Please tell us **how much of a problem** each one has been for you during the **past ONE month** by circling:

0 if it is **never** a problem
1 if it is **almost never** a problem
2 if it is **sometimes** a problem
3 if it is **often** a problem
4 if it is **almost always** a problem

There are no right or wrong answers.
If you do not understand a question, please ask for help.

Not to be reproduced without permission
Copyright © 1998 JW Varni, Ph.D.
All rights reserved

*In the past **ONE** month, how much of a **problem** has this been for you…*

**HEART PROBLEMS AND TREATMENT** ***(problems with…)***	**Never**	**Almost Never**	**Some-times**	**Often**	**Almost Always**
1. I get out of breath when I do sports activity or exercise	0	1	2	3	4
2. My chest hurts or feels tight when I do sports activity or exercise	0	1	2	3	4
3. I catch colds easily	0	1	2	3	4
4. I feel my heart beating fast	0	1	2	3	4
5. My lips turn blue when I run	0	1	2	3	4
6. I wake up at night with trouble breathing	0	1	2	3	4
7. I have to rest more than my friends	0	1	2	3	4

If you are currently taking heart medicine, please answer the following…
Otherwise, please skip to "Perceived Physical Appearance".

**TREATMENT II** ***(problems with…)***	**Never**	**Almost Never**	**Some-times**	**Often**	**Almost Always**
1. I refuse to take my heart medicine	0	1	2	3	4
2. It is hard for me to take my heart medicine	0	1	2	3	4
3. I forget to take my heart medicine	0	1	2	3	4
4. My heart medicine makes me feel sick	0	1	2	3	4
5. I worry about side effects from my medicine	0	1	2	3	4

**PERCEIVED PHYSICAL APPEARANCE** ***(problems with…)***	**Never**	**Almost Never**	**Some-times**	**Often**	**Almost Always**
1. I feel I am not good looking	0	1	2	3	4
2. I don't like other people to see my scars	0	1	2	3	4
3. I am embarrassed when others see my body	0	1	2	3	4

**TREATMENT ANXIETY** ***(problems with…)***	**Never**	**Almost Never**	**Some-times**	**Often**	**Almost Always**
1. I get scared when I am waiting to see the doctor	0	1	2	3	4
2. I get scared when I have to go to the doctor	0	1	2	3	4
3. I get scared when I have to go to the hospital	0	1	2	3	4
4. I get scared when I have to have medical treatments	0	1	2	3	4

Not to be reproduced without permission
Copyright © 1998 JW Varni, Ph.D.
All rights reserved

*In the past **ONE** month, how much of a **problem** has this been for you…*

COGNITIVE PROBLEMS ***(problems with…)***	**Never**	**Almost Never**	**Some-times**	**Often**	**Almost Always**
1. It is hard for me to figure out what to do when something bothers me	0	1	2	3	4
2. I have trouble solving math problems	0	1	2	3	4
3. I have trouble writing school papers or reports	0	1	2	3	4
4. It is hard for me to pay attention to things	0	1	2	3	4
5. It is hard for me to remember what I read	0	1	2	3	4

COMMUNICATION ***(problems with…)***	**Never**	**Almost Never**	**Some-times**	**Often**	**Almost Always**
1. It is hard for me to tell the doctors and nurses how I feel	0	1	2	3	4
2. It is hard for me to ask the doctors and nurses questions	0	1	2	3	4
3. It is hard for me to explain my heart problem to other people	0	1	2	3	4

Not to be reproduced without permission
Copyright © 1998 JW Varni, Ph.D.
All rights reserved

ID#_______________________

Date:_______________________

PedsQL™

Cardiac Module

Version 3.0

PARENT REPORT for **TEENS** (ages **13-18**)

DIRECTIONS

Teens with heart conditions sometimes have special problems. On the following page is a list of things that might be a problem for **your teen**. Please tell us **how much of a problem** each one has been for **your teen** during the **past ONE month** by circling:

0 if it is **never** a problem
1 if it is **almost never** a problem
2 if it is **sometimes** a problem
3 if it is **often** a problem
4 if it is **almost always** a problem

There are no right or wrong answers.
If you do not understand a question, please ask for help.

Not to be reproduced without permission

Copyright © 1998 JW Varni, Ph.D.
All rights reserved

*In the past **ONE month,** how much of a **problem** has your teen had with …*

HEART PROBLEMS AND TREATMENT *(problems with…)*	Never	Almost Never	Some-times	Often	Almost Always
1. Getting out of breath while doing sports activity or exercise	0	1	2	3	4
2. Chest pain or tightness while doing sports activity or exercise	0	1	2	3	4
3. Catching colds easily	0	1	2	3	4
4. Fast heartbeat	0	1	2	3	4
5. His/her lips turning blue when running	0	1	2	3	4
6. Waking up at night with trouble breathing	0	1	2	3	4
7. Having to rest more than his/her friends	0	1	2	3	4

If your child is currently taking heart medicine, please answer the following…
Otherwise, please skip to "Perceived Physical Appearance".

TREATMENT II *(problems with…)*	Never	Almost Never	Some-times	Often	Almost Always
1. Refusing to take heart medicine	0	1	2	3	4
2. Difficulty taking heart medicine	0	1	2	3	4
3. Forgetting to take heart medicine	0	1	2	3	4
4. Heart medicine making him/her feel sick	0	1	2	3	4
5. Worrying about side effects from his/her medicine	0	1	2	3	4

PERCEIVED PHYSICAL APPEARANCE *(problems with…)*	Never	Almost Never	Some-times	Often	Almost Always
1. Feeling that he/she is not good looking	0	1	2	3	4
2. Not liking other people to see his/her scars	0	1	2	3	4
3. Being embarrassed about others seeing his/her body	0	1	2	3	4

TREATMENT ANXIETY *(problems with…)*	Never	Almost Never	Some-times	Often	Almost Always
1. Getting anxious when waiting to see the doctor	0	1	2	3	4
2. Getting anxious about going to the doctor	0	1	2	3	4
3. Getting anxious about going to the hospital	0	1	2	3	4
4. Getting anxious when he/she has to have medical treatments	0	1	2	3	4

*In the past **ONE month,** how much of a **problem** has your teen had with …*

Not to be reproduced without permission
Copyright © 1998 JW Varni, Ph.D. All rights reserved

COGNITIVE PROBLEMS *(problems with…)*	Never	Almost Never	Some-times	Often	Almost Always
1. Figuring out what to do when something bothers him/her	0	1	2	3	4
2. Trouble solving math problems	0	1	2	3	4
3. Trouble writing school papers or reports	0	1	2	3	4
4. Difficulty paying attention to things	0	1	2	3	4
5. Remembering what he/she reads	0	1	2	3	4

COMMUNICATION *(problems with…)*	Never	Almost Never	Some-times	Often	Almost Always
1. Telling the doctors and nurses how he/she feels	0	1	2	3	4
2. Asking the doctors or nurses questions	0	1	2	3	4
3. Explaining his/her heart problem to other people	0	1	2	3	4

Not to be reproduced without permission
Copyright © 1998 JW Varni, Ph.D.
All rights reserved

ID# ___________________________

Date:__________________________

PedsQL ™

Cardiac Module

Version 3.0

CHILD REPORT (ages **8-12**)

DIRECTIONS

Children with heart conditions sometimes have special problems. Please tell us **how much of a problem** each one has been for you during the **past ONE month** by circling:

0 if it is **never** a problem
1 if it is **almost never** a problem
2 if it is **sometimes** a problem
3 if it is **often** a problem
4 if it is **almost always** a problem

There are no right or wrong answers.
If you do not understand a question, please ask for help.

Not to be reproduced without permission
Copyright © 1998 JW Varni, Ph.D.
All rights reserved

*In the past **ONE** month, how much of a **problem** has this been for you…*

**HEART PROBLEMS AND TREATMENT** ***(problems with…)***	**Never**	**Almost Never**	**Some-times**	**Often**	**Almost Always**
1. I get out of breath when I do sports activity or exercise	0	1	2	3	4
2. My chest hurts or feels tight when I do sports activity or exercise	0	1	2	3	4
3. I catch colds easily	0	1	2	3	4
4. I feel my heart beating fast	0	1	2	3	4
5. My lips turn blue when I run	0	1	2	3	4
6. I wake up at night with trouble breathing	0	1	2	3	4
7. I have to rest more than my friends	0	1	2	3	4

If you are currently taking heart medicine, please answer the following…
Otherwise, please skip to "Perceived Physical Appearance".

**TREATMENT II** ***(problems with…)***	**Never**	**Almost Never**	**Some-times**	**Often**	**Almost Always**
1. I refuse to take my heart medicine	0	1	2	3	4
2. It is hard for me to take my heart medicine	0	1	2	3	4
3. I forget to take my heart medicine	0	1	2	3	4
4. My heart medicine makes me feel sick	0	1	2	3	4
5. I worry about how my medicines affect my body	0	1	2	3	4

**PERCEIVED PHYSICAL APPEARANCE** ***(problems with…)***	**Never**	**Almost Never**	**Some-times**	**Often**	**Almost Always**
1. I feel I am not good looking	0	1	2	3	4
2. I don't like other people to see my scars	0	1	2	3	4
3. I am embarrassed when others see my body	0	1	2	3	4

**TREATMENT ANXIETY** ***(problems with…)***	**Never**	**Almost Never**	**Some-times**	**Often**	**Almost Always**
1. I get scared when I am waiting to see the doctor	0	1	2	3	4
2. I get scared when I have to go to the doctor	0	1	2	3	4
3. I get scared when I have to go to the hospital	0	1	2	3	4
4. I get scared when I have to have medical treatments	0	1	2	3	4

Not to be reproduced without permission
Copyright © 1998 JW Varni, Ph.D.
All rights reserved

*In the past **ONE** month, how much of a **problem** has this been for you…*

COGNITIVE PROBLEMS ***(problems with…)***	**Never**	**Almost Never**	**Some-times**	**Often**	**Almost Always**
1. It is hard for me to figure out what to do when something bothers me	0	1	2	3	4
2. I have trouble solving math problems	0	1	2	3	4
3. I have trouble writing school papers or reports	0	1	2	3	4
4. It is hard for me to pay attention to things	0	1	2	3	4
5. It is hard for me to remember what I read	0	1	2	3	4

COMMUNICATION ***(problems with…)***	**Never**	**Almost Never**	**Some-times**	**Often**	**Almost Always**
1. It is hard for me to tell the doctors and nurses how I feel	0	1	2	3	4
2. It is hard for me to ask the doctors and nurses questions	0	1	2	3	4
3. It is hard for me to explain my heart problem to other people	0	1	2	3	4

Not to be reproduced without permission
Copyright © 1998 JW Varni, Ph.D.
All rights reserved

ID#________________________

Date:________________________

PedsQL™
Cardiac Module

Version 3.0

PARENT REPORT for **CHILDREN** (ages **8-12**)

DIRECTIONS

Children with heart conditions sometimes have special problems. On the following page is a list of things that might be a problem for **your child**. Please tell us **how much of a problem** each one has been for **your child** during the **past ONE month** by circling:

0 if it is **never** a problem
1 if it is **almost never** a problem
2 if it is **sometimes** a problem
3 if it is **often** a problem
4 if it is **almost always** a problem

There are no right or wrong answers.
If you do not understand a question, please ask for help.

Not to be reproduced without permission
Copyright © 1998 JW Varni, Ph.D.
All rights reserved

*In the past **ONE month,** how much of a **problem** has your child had with …*

**HEART PROBLEMS AND TREATMENT** ***(problems with…)***	**Never**	**Almost Never**	**Some-times**	**Often**	**Almost Always**
1. Getting out of breath while doing sports activity or exercise	0	1	2	3	4
2. Chest pain or tightness while doing sports activity or exercise	0	1	2	3	4
3. Catching colds easily	0	1	2	3	4
4. Fast heartbeat	0	1	2	3	4
5. His/her lips turning blue when running	0	1	2	3	4
6. Waking up at night with trouble breathing	0	1	2	3	4
7. Having to rest more than his/her friends	0	1	2	3	4

If your child is currently taking heart medicine, please answer the following…
Otherwise, please skip to "Perceived Physical Appearance".

**TREATMENT II** ***(problems with…)***	**Never**	**Almost Never**	**Some-times**	**Often**	**Almost Always**
1. Refusing to take heart medicine	0	1	2	3	4
2. Difficulty taking heart medicine	0	1	2	3	4
3. Forgetting to take heart medicine	0	1	2	3	4
4. Heart medicine making him/her feel sick	0	1	2	3	4
5. Worry about side effects from his/her medicine	0	1	2	3	4

**PERCEIVED PHYSICAL APPEARANCE** ***(problems with…)***	**Never**	**Almost Never**	**Some-times**	**Often**	**Almost Always**
1. Feeling that he/she is not good looking	0	1	2	3	4
2. Not liking other people to see his/her scars	0	1	2	3	4
3. Being embarrassed about others seeing his/her body	0	1	2	3	4

**TREATMENT ANXIETY** ***(problems with…)***	**Never**	**Almost Never**	**Some-times**	**Often**	**Almost Always**
1. Getting anxious when waiting to see the doctor	0	1	2	3	4
2. Getting anxious about going to the doctor	0	1	2	3	4
3. Getting anxious about going to the hospital	0	1	2	3	4
4. Getting anxious when he/she has to have medical treatments	0	1	2	3	4

Not to be reproduced without permission
Copyright © 1998 JW Varni, Ph.D.
All rights reserved

*In the past **ONE month,** how much of a **problem** has your child had with …*

COGNITIVE PROBLEMS *(problems with…)*	Never	Almost Never	Some-times	Often	Almost Always
1. Figuring out what to do when something bothers him/her	0	1	2	3	4
2. Trouble solving math problems	0	1	2	3	4
3. Trouble writing school papers or reports	0	1	2	3	4
4. Difficulty paying attention to things	0	1	2	3	4
5. Remembering what he/she reads	0	1	2	3	4

COMMUNICATION *(problems with…)*	Never	Almost Never	Some-times	Often	Almost Always
1. Telling the doctors and nurses how he/she feels	0	1	2	3	4
2. Asking the doctors or nurses questions	0	1	2	3	4
3. Explaining his/her heart problem to other people	0	1	2	3	4

Not to be reproduced without permission
Copyright © 1998 JW Varni, Ph.D.
All rights reserved

ID# ___________________________

Date:__________________________

PedsQL™

Cardiac Module

Version 3.0

YOUNG CHILD REPORT (ages **5-7**)

Instructions for interviewer:

I am going to ask you some questions about things that might be a problem for some children. I want to know how much of a problem any of these things might be for you.

Show the child the template and point to the responses as you read**.**

If it is <u>not at all</u> a problem for you, point to the smiling face

If it is <u>sometimes</u> a problem for you, point to the middle face

If it is a problem for you <u>a lot</u>, point to the frowning face

I will read each question. Point to the pictures to show me how much of a problem it is for you. Let's try a practice one first.

	Not at all	Sometimes	A lot
**Is it hard for you to snap your fingers**	☺	😐	☹

Ask the child to demonstrate snapping his or her fingers to determine whether or not the question was answered correctly. Repeat the question if the child demonstrates a response that is different from his or her action.

Not to be reproduced without permission

Copyright © 1998 JW Varni, Ph.D.
All rights reserved

Think about how you have been doing for the last few weeks. Please listen carefully to each sentence and tell me how much of a problem this is for you.

After reading the item, gesture to the template. If the child hesitates or does not seem to understand how to answer, read the response options while pointing at the faces.

**HEART PROBLEMS AND TREATMENT** ***(problems with…)***	**Not at all**	**Some-times**	**A lot**
1. Is it hard for you to breathe when you do sports activity or exercise	0	2	4
2. Does your chest hurt when you do sports activity or exercise	0	2	4
3. Do you catch colds more than other kids	0	2	4
4. Do you feel your heart beating fast	0	2	4
5. Do others tell you that your lips turn blue when you run	0	2	4
6. Do you wake up at night with trouble breathing	0	2	4
7. Do you have to rest more than your friends	0	2	4

If you are currently taking heart medicine, please answer the following…
Otherwise, please skip to "Perceived Physical Appearance".

**TREATMENT II** ***(problems with…)***	**Not at all**	**Some-times**	**A lot**
1. Do you say no to taking your heart medicine	0	2	4
2. Do you have a hard time taking your heart medicine	0	2	4
3. Does your heart medicine make you feel sick	0	2	4

**PERCEIVED PHYSICAL APPEARANCE** ***(problems with…)***	**Not at all**	**Some-times**	**A lot**
1. Do you feel you don't look good	0	2	4
2. Do you not like other people to see your scars	0	2	4
3. Do other kids tease you when they see your scars	0	2	4

**TREATMENT ANXIETY** ***(problems with…)***	**Not at all**	**Some-times**	**A lot**
1. Do you get scared when you are waiting to see the doctor	0	2	4
2. Do you get scared when you have to go to the doctor	0	2	4
3. Do you get scared when you have to go to the hospital	0	2	4
4. Do you get scared when you have to have heart tests	0	2	4

Not to be reproduced without permission
Copyright © 1998 JW Varni, Ph.D.
All rights reserved

Think about how you have been doing for the last few weeks. Please listen carefully to each sentence and tell me how much of a problem this is for you.

**COGNITIVE PROBLEMS *(problems with…)***	**Not at all**	**Some-times**	**A lot**
1. Is it hard for you to know what to do when something bothers you	0	2	4
2. Do you have trouble with numbers or math worksheets	0	2	4
3. Do you have trouble writing letters or words	0	2	4
4. Is it hard for you to listen to the teacher	0	2	4
5. Is it hard for you to remember what is read to you	0	2	4

**COMMUNICATION *(problems with…)***	**Not at all**	**Some-times**	**A lot**
1. Is it hard for you to tell the doctors and nurses how you feel	0	2	4
2. Is it hard for you to ask the doctors and nurses questions	0	2	4
3. Is it hard for you to explain your heart problem to other people	0	2	4

Not to be reproduced without permission
Copyright © 1998 JW Varni, Ph.D.
All rights reserved

How much of a problem is this for you?

Not at all	Sometimes	A lot
☺	😐	☹

Not to be reproduced without permission
Copyright © 1998 JW Varni, Ph.D. All rights reserved

ID#___________________________

Date:__________________________

PedsQL™
Cardiac Module

Version 3.0

PARENT REPORT for **YOUNG CHILDREN** (ages **5-7**)

DIRECTIONS

Children with heart conditions sometimes have special problems. On the following page is a list of things that might be a problem for **your child**. Please tell us **how much of a problem** each one has been for **your child** during the **past ONE month** by circling:

0 if it is **never** a problem
1 if it is **almost never** a problem
2 if it is **sometimes** a problem
3 if it is **often** a problem
4 if it is **almost always** a problem

There are no right or wrong answers.
If you do not understand a question, please ask for help.

Not to be reproduced without permission

Copyright © 1998 JW Varni, Ph.D
All rights reserved

*In the past **ONE month,** how much of a **problem** has your child had with …*

**HEART PROBLEMS AND TREATMENT** *(problems with…)*	Never	Almost Never	Some-times	Often	Almost Always
1. Getting out of breath while doing sports activity or exercise	0	1	2	3	4
2. Chest pain or tightness while doing sports activity or exercise	0	1	2	3	4
3. Catching colds easily	0	1	2	3	4
4. Fast heartbeat	0	1	2	3	4
5. His/her lips turning blue when running	0	1	2	3	4
6. Waking up at night with trouble breathing	0	1	2	3	4
7. Having to rest more than his/her friends	0	1	2	3	4

If your child is currently taking heart medicine, please answer the following…
Otherwise, please skip to "Perceived Physical Appearance".

**TREATMENT II** *(problems with…)*	Never	Almost Never	Some-times	Often	Almost Always
1. Refusing to take heart medicine	0	1	2	3	4
2. Difficulty taking heart medicine	0	1	2	3	4
3. Heart medicine making him/her feel sick	0	1	2	3	4

**PERCEIVED PHYSICAL APPEARANCE** *(problems with…)*	Never	Almost Never	Some-times	Often	Almost Always
1. Feeling that he/she is not good looking	0	1	2	3	4
2. Not liking other people to see his/her scars	0	1	2	3	4
3. Getting teased when other kids see his/her scars	0	1	2	3	4

**TREATMENT ANXIETY** *(problems with…)*	Never	Almost Never	Some-times	Often	Almost Always
1. Getting anxious when waiting to see the doctor	0	1	2	3	4
2. Getting anxious about going to the doctor	0	1	2	3	4
3. Getting anxious about going to the hospital	0	1	2	3	4
4. Getting anxious when he/she has to have medical treatments	0	1	2	3	4

Not to be reproduced without permission
Copyright © 1998 JW Varni, Ph.D.
All rights reserved

*In the past **ONE month,** how much of a **problem** has your child had with …*

**COGNITIVE PROBLEMS *(problems with…)***	**Never**	**Almost Never**	**Some-times**	**Often**	**Almost Always**
1. Figuring out what to do when something bothers him/her	0	1	2	3	4
2. Trouble with numbers or math worksheets	0	1	2	3	4
3. Trouble writing letters or words	0	1	2	3	4
4. Difficulty paying attention to the teacher	0	1	2	3	4
5. Remembering what is read to him/her	0	1	2	3	4

**COMMUNICATION *(problems with…)***	**Never**	**Almost Never**	**Some-times**	**Often**	**Almost Always**
1. Telling the doctors and nurses how he/she feels	0	1	2	3	4
2. Asking the doctors or nurses questions	0	1	2	3	4
3. Explaining his/her heart problem to other people	0	1	2	3	4

Not to be reproduced without permission

Copyright © 1998 JW Varni, Ph.D.
All rights reserved

ID#______________________

Date:______________________

PedsQL™

Cardiac Module

Version 3.0

PARENT REPORT for **TODDLERS** (ages **2-4**)

DIRECTIONS

Children with heart conditions sometimes have special problems. On the following page is a list of things that might be a problem for **your child**. Please tell us **how much of a problem** each one has been for **your child** during the **past ONE month** by circling:

0 if it is **never** a problem
1 if it is **almost never** a problem
2 if it is **sometimes** a problem
3 if it is **often** a problem
4 if it is **almost always** a problem

There are no right or wrong answers.
If you do not understand a question, please ask for help.

Not to be reproduced without permission
Copyright © 1998 JW Varni, Ph.D.
All rights reserved

*In the past **ONE month,** how much of a **problem** has your child had with …*

HEART PROBLEMS AND TREATMENT (problems with…)	Never	Almost Never	Some-times	Often	Almost Always
1. Getting out of breath while doing active play or exercise	0	1	2	3	4
2. Chest pain or tightness while doing active play or exercise	0	1	2	3	4
3. Catching colds easily	0	1	2	3	4
4. Fast heartbeat	0	1	2	3	4
5. His/her lips turning blue when running	0	1	2	3	4
6. Waking up at night with trouble breathing	0	1	2	3	4
7. Having to rest more than his/her friends	0	1	2	3	4

If your child is currently taking heart medicine, please answer the following…
Otherwise, please skip to "Perceived Physical Appearance".

TREATMENT II *(problems with…)*	Never	Almost Never	Some-times	Often	Almost Always
1. Refusing to take heart medicine	0	1	2	3	4
2. Difficulty taking heart medicine	0	1	2	3	4
3. Heart medicine making him/her feel sick	0	1	2	3	4

PERCEIVED PHYSICAL APPEARANCE *(problems with…)*	Never	Almost Never	Some-times	Often	Almost Always
1. Feeling that he/she is not good looking	0	1	2	3	4
2. Not liking other people to see his/her scars	0	1	2	3	4
3. Being embarrassed about others seeing his/her body	0	1	2	3	4

TREATMENT ANXIETY *(problems with…)*	Never	Almost Never	Some-times	Often	Almost Always
1. Getting anxious when waiting to see the doctor	0	1	2	3	4
2. Getting anxious about going to the doctor	0	1	2	3	4
3. Getting anxious about going to the hospital	0	1	2	3	4
4. Getting anxious when he/she has to have medical treatments	0	1	2	3	4

*In the past **ONE month,** how much of a **problem** has your child had with …*

Not to be reproduced without permission
Copyright © 1998 JW Varni, Ph.D.
All rights reserved

COGNITIVE PROBLEMS *(problems with…)*	Never	Almost Never	Some-times	Often	Almost Always
1. Figuring out what to do when something bothers him/her	0	1	2	3	4
2. Difficulty paying attention to things	0	1	2	3	4
3. Remembering what is read to him/her	0	1	2	3	4

COMMUNICATION *(problems with…)*	Never	Almost Never	Some-times	Often	Almost Always
1. Telling the doctors and nurses how he/she feels	0	1	2	3	4
2. Asking the doctors or nurses questions	0	1	2	3	4
3. Explaining his/her heart problem to other people	0	1	2	3	4

Not to be reproduced without permission
Copyright © 1998 JW Varni, Ph.D.
All rights reserved
